# The Impact of Multidisciplinary Transitional Care Interventions for Complex Care Needs: A Systematic Review and Meta-Analysis

**DOI:** 10.1093/geront/gnaf088

**Published:** 2025-03-05

**Authors:** Romain Collet, Juul van Grootel, Johanna van Dongen, Suzanne Wiertsema, Raymond Ostelo, Marike van der Schaaf, Elena Lazzari, Edwin Geleijn, Mel Major, Marike van der Leeden

**Affiliations:** Faculty of Science, Department of Health Sciences, Vrije University Amsterdam, Amsterdam Movement Sciences Research Institute, Musculoskeletal Health, Amsterdam, The Netherlands; Department of Epidemiology and Data Science, Amsterdam UMC, Vrije Universiteit Amsterdam, Amsterdam, The Netherlands; Amsterdam Movement Sciences, Ageing, and Vitality, Amsterdam, The Netherlands; Department of Rehabilitation Medicine, Amsterdam UMC Location University of Amsterdam, Amsterdam, The Netherlands; Faculty of Science, Department of Health Sciences, Vrije University Amsterdam, Amsterdam Movement Sciences Research Institute, Musculoskeletal Health, Amsterdam, The Netherlands; Department of Rehabilitation Medicine, Amsterdam UMC Location University of Amsterdam, Amsterdam, The Netherlands; Faculty of Science, Department of Health Sciences, Vrije University Amsterdam, Amsterdam Movement Sciences Research Institute, Musculoskeletal Health, Amsterdam, The Netherlands; Department of Epidemiology and Data Science, Amsterdam UMC, Vrije Universiteit Amsterdam, Amsterdam, The Netherlands; Amsterdam Movement Sciences, Ageing, and Vitality, Amsterdam, The Netherlands; Department of Rehabilitation Medicine, Amsterdam UMC Location University of Amsterdam, Amsterdam, The Netherlands; Faculty of Behavioural and Movement Sciences, Department of Human Movement Sciences, Vrije University Amsterdam, Amsterdam, The Netherlands; Department of Rehabilitation Medicine, Amsterdam UMC Location Vrije Universiteit Amsterdam, Amsterdam, The Netherlands; Faculty of Health, Department of Physical Therapy, Amsterdam University of Applied Sciences, Amsterdam, The Netherlands; Faculty of Health, Center of Expertise Urban Vitality, Amsterdam University of Applied Sciences, Amsterdam, The Netherlands; Amsterdam Movement Sciences, Ageing, and Vitality, Amsterdam, The Netherlands; Department of Rehabilitation Medicine, Amsterdam UMC Location Vrije Universiteit Amsterdam, Amsterdam, The Netherlands

**Keywords:** Continuity of patient care, Meta-analysis, Multimorbidity, Quality of life, Transitional care

## Abstract

**Background and Objectives:**

Multidisciplinary transitional care interventions (MTCIs) ensure care coordination and continuity after hospital discharge while addressing (older) patients’ complex care needs related to their physical, nutritional, cognitive, and/or psychological status. This study aimed to identify, critically appraise, and synthesize the current body of evidence investigating the effectiveness of such interventions.

**Research Design and Methods:**

Medline, Embase, CINAHL, and CENTRAL were searched for randomized controlled trials assessing MTCIs’ impact on readmissions, mortality, and health-related outcomes from inception to July 2024. Risk of bias was evaluated with the Risk of Bias-2 tool. Subgroup analyses assessed whether different intervention types affected outcomes differently. The certainty of the evidence was assessed with the Grading of Recommendations Assessment, Development, and Evaluation approach and the credibility of subgroup analyses with the Instrument to evaluate the Credibility of Effect Modification Analyses.

**Results:**

Forty-nine trials involving 25,566 patients were included. There was low certainty that MTCIs reduced readmissions (relative risk [RR] = 0.88; 95% confidence intervals [95% CI] = 0.80 to 0.96) and high certainty for reduced mortality (RR = 0.92; 95% CI = 0.84 to 1.01). There was low to moderate certainty that MTCIs improved physical quality of life (standardized mean difference [SMD] = 0.54; 95% CI = −0.06 to 1.15), mental quality of life (SMD = 0.44; 95% CI = −0.08 to 0.96), patient satisfaction (SMD = 0.49; 95% CI = −0.14 to 1.12), and physical performance (SMD = 0.49; 95% CI = −0.11 to 1.10). Subgroup analysis revealed a larger and statistically significant effect on physical performance in more complex interventions (SMD = 0.83; 95% CI = 0.02 to 1.65).

**Discussion and Implications:**

These findings suggest that MTCIs can reduce readmissions and mortality while improving quality of life and physical performance. Further investigations should focus on tailoring MTCIs to specific contexts to maximize their impact.

## Graphical Abstract

**Graphical Abstract:**
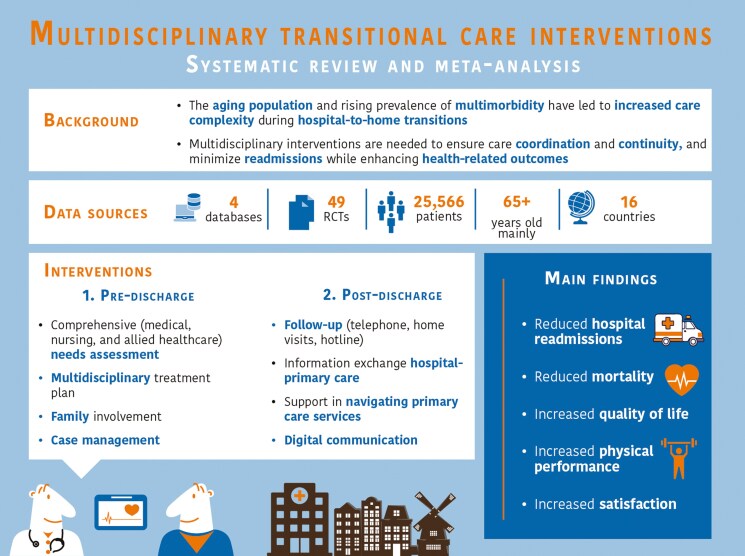

## Background and Objectives

Over the past decades, the complexity of patient care during hospitalization and after discharge has increased, primarily due to the aging population and the rising prevalence of multimorbidity (Chowdhury et al., 2023; Pearson-Stuttard et al., 2019). In 2021, the global prevalence of multimorbidity was 37.2%, and recent research shows that multimorbidity is associated with aging (Tazzeo et al., 2023). Older patients and/or patients with multimorbidity typically have more *complex care needs*—which include medical, nursing, and allied healthcare needs related to physical, nutritional, cognitive, and/or psychosocial status—because of interactions between many factors in their socioecological environment and inherited biology (e.g., lack of social support, psychological or emotional dysregulation, inappropriate utilization of healthcare services; Sturmberg et al., 2021).

Research indicates that hospitalized patients, especially those with multimorbidity, often experience care fragmentation after discharge, which refers to limited, noncontinuous, episodic, and disorganized care across multiple healthcare practitioners and settings (Cohen-Mekelburg et al., 2019; Joo, 2023; Prior et al., 2023). Fragmented care may result in unplanned readmissions, emergency department visits, or mortality, as patients, particularly those with complex care needs, may not have received the necessary follow-up care in the community after hospital discharge (Joo, 2023; Prior et al., 2023). Evidence suggests, for example, that patients with complex care needs require more support, preferably tailored, to navigate their way through community care services (van Grootel, Collet, Major, et al., 2024). However, current practices regarding hospital-to-home transitions seem to be suboptimal. Patients, family members, and healthcare professionals often experience a lack of coordination and continuity of care after discharge, partially attributed to rare or inefficient communication between healthcare professionals across settings (van Grootel, Collet, van Dongen, et al., 2024). Therefore, there is a need for effective transitional care interventions aimed at ensuring coordination and continuity of care across various locations or levels of care (Goodwin, 2021).

Several systematic reviews investigating the effects of transitional care interventions suggest that they reduce hospital readmission rates and enhance health-related outcomes (Li, Li, et al., 2021; Rasmussen et al., 2021). Historically, these interventions were often monodisciplinary and mainly focused on ensuring medication adherence or medical care needs. However, with the increased complexity of patient follow-up care, a more comprehensive approach might be needed to encompass medical care, nursing care, and care provided by allied health professionals in the community. In recent years, such interventions have been developed and evaluated and are referred to as multidisciplinary transitional care interventions in this systematic review (Evangelista et al., 2023; Jepma et al., 2021; Liu et al., 2023; Markle-Reid et al., 2023).

Multidisciplinary transitional care interventions involve a range of hospital and community care professionals, including physicians, nurse practitioners, physical therapists, occupational therapists, dieticians, social workers, and speech and language therapists. However, the specific content of such intervention can differ widely, ranging from a discharge summary provided to patients leaving the hospital to interventions that employ dedicated multidisciplinary care teams that coordinate and provide tailored recovery trajectories, both in-hospital and after discharge. A recent systematic review by Tyler et al. (2023) investigated if the number of components (e.g., a follow-up phone call or a medication review) of a transitional care intervention influenced its effectiveness on healthcare utilization. It revealed that, compared to usual care, interventions with fewer components were more effective at reducing healthcare utilization, whereas more complex interventions increased patient satisfaction. However, Tyler et al. (2023) did not differentiate multidisciplinary interventions targeting patients’ complex care needs from those solely addressing medical needs, nor did they assess the impact of such interventions on health-related outcomes, such as physical, cognitive, and psychological status. Hence, there remains a lack of conclusive evidence regarding the effectiveness of interventions that employ a multidisciplinary approach and simultaneously address patients’ complex care needs, that is, a combination of medical, nursing, and/or allied healthcare needs.

Therefore, this systematic review and meta-analysis aimed to identify, critically appraise, and synthesize the current body of randomized controlled trials (RCTs) investigating the effectiveness of multidisciplinary transitional care interventions on hospital readmissions and health-related outcomes. A secondary aim of this review was to assess whether different types of interventions have different effects on readmissions and health outcomes. This secondary aim was added to account for the relatively wide variability in the content of transitional care interventions and to provide insight into the most effective intervention types that can serve in the future (re)design of optimal multidisciplinary transitional care interventions.

## Research Design and Methods

This systematic review adhered to the Preferred Reporting Items for Systematic Reviews and Meta-Analyses (PRISMA) and was registered in PROSPERO (CRD42023421423; Page et al., 2021). The PROSPERO protocol describes a mixed-method systematic review investigating facilitators and barriers to the implementation, as well as the (cost-)effectiveness, of multidisciplinary transitional care interventions. Because of the large number of studies and the richness of the data identified, the current review focuses on RCTs evaluating the effectiveness of multidisciplinary transitional care interventions on readmissions and health-related outcomes. A systematic review on the facilitators and barriers to the implementation and the (cost-)effectiveness will be published separately (Collet et al., 2025).

### Data Sources and Searches

Searches were conducted in Medline, Embase, CINAHL, and CENTRAL by an experienced medical information specialist from inception until July 2024. A combination of several groups of index terms and respective keywords was used: transitional care, continuity of patient care, patient readmission, multimorbidity, etc. Additional records were identified by screening the reference lists of the included papers and the snowball method using Google Scholar (Wohlin et al., 2022). The complete search strategy for each database is available in Supplementary Appendix 1.

### Study Selection

Randomized controlled trials or cluster RCTs were eligible for inclusion if they: (1) included adult patients admitted to a hospital regardless of their conditions, (2) evaluated multidisciplinary transitional care interventions for hospital-to-home transitions (i.e., delivered by at least two healthcare professionals of distinct expertise, e.g., a nurse practitioner and a physical therapist) addressing medical, nursing, and allied healthcare needs (i.e., needs related to physical, cognitive, nutritional, and/or psychosocial status), (3) compared the intervention to usual care, and (4) reported on unplanned readmissions, mortality, emergency department visits, quality of life, physical status, cognitive status, psychological distress, and/or patient satisfaction with the intervention. Studies focusing on populations suffering from mental illnesses or requiring palliative care were excluded. Retrieved records were imported into Rayyan (http://rayyan.qcri.org). After duplicate removal, titles and abstracts were screened by all authors in pairs of two. Next, pairs of reviewers (R. Collet, J. v. Grootel, and E. Lazzari) independently assessed full texts for eligibility. Disagreements between reviewers were resolved during consensus meetings.

### Outcome Measures

The primary outcomes were all-cause unplanned hospital readmission (number of individuals readmitted), mortality (number of individuals who died), and emergency department visit (number of individuals who visited the emergency department) rates, as well as quality of life (self-reported questionnaires). Secondary outcomes included physical performance (self-reported questionnaires on difficulties in activities of daily living), physical capacity (measured, e.g., with submaximal exercise tests), cognitive status (self-reported questionnaires), psychological distress (self-reported questionnaires on anxiety and depression), and patient satisfaction (self-reported questionnaires).

### Data Extraction

For each study, we extracted the following information: author’s name, publication year, country, study design, description of the main components of the intervention, the types of hospital and community care professionals delivering the intervention, patient characteristics (i.e., disease or condition, sample size, mean age, and gender distribution), the type of control group, outcome measures, and outcome measurement instruments. One reviewer (R. Collet) extracted data, which were then checked for consistency by a second reviewer (J. v. Grootel).

### Risk of Bias Assessment

Two reviewers (R. Collet and J. v. Grootel) independently evaluated the risk of bias of each article using the Risk of Bias, version 2 (RoB2) or RoB2 for cluster randomized trials tools (Crocker et al., 2023; Sterne et al., 2019). These tools provide a comprehensive framework for assessing five key domains: the randomization process, deviations from the intended interventions, missing outcome data, measurement of the outcome, and selection of the reported results. Each domain within the RoB2 tool was scored as “low risk,” “some concerns,” or “high risk” based on the identified issues. With the help of the algorithm provided in the Cochrane guidance document (Sterne et al., 2019), a final judgment was made for each paper, which was classified as having a “low,” “some concerns,” or “high” risk of bias.

### Statistical Analysis, Synthesis, and GRADE Assessment

All analyses were conducted in R, using the meta package (Balduzzi et al., 2019). To pool effect sizes based on dichotomous data (i.e., readmissions, mortality, and emergency department visits), we used the Mantel–Haenszel method and calculated risk ratios (Mantel & Haenszel, 1959). To pool effect sizes of continuous outcomes measured with different instruments or tests, we calculated standardized mean differences and reported 95% confidence intervals. When medians and interquartile ranges were reported, we converted them into means and standard deviations if the interquartile range was symmetrical around the median. We then performed meta-analyses if postintervention outcomes for intervention and control groups were reported in two studies or more. We assessed heterogeneity by visually examining forest plots and assessing the *I*² statistic. When there was an indication of high heterogeneity (i.e., when *I*² > 50%), we presented the results of the random effects meta-analyses. Otherwise, we presented the results of the fixed effects meta-analyses. We conducted a narrative synthesis when a meta-analysis was inappropriate (e.g., when results were reported in a graph where we could not precisely retrieve outcome values or when only *p* values were reported).

For interpreting the results of the individual comparisons, we followed the Grading of Recommendations, Assessment, Development, and Evaluation (GRADE) methodology (Guyatt et al., 2013). Instead of solely looking at the statistical significance of comparisons, the GRADE approach “downgrades” the level of evidence of a comparison based on five factors to provide a more solid foundation for clinical decision-making (Schünemann, 2022):

1. *Overall risk of bias:* downgrading the certainty of the evidence when the methodological quality of the body of evidence was a threat (i.e., if >50% of the trials were assessed as high risk).
2. *Consistency*: downgrading the certainty of the evidence when effects were inconsistent across studies (i.e., if *I*² was >50%).
3. *Precision:* downgrading the certainty of the evidence when confidence intervals were wide, hence when the uncertainty around the pooled effect size was considered too large (i.e., if one or both boundaries of the confidence interval suggested inferences appreciably different from the point estimate). When the confidence interval was very wide, we downgraded two levels (Zeng et al., 2022).
4. *Directness*: downgrading the certainty of the evidence when evidence was obtained from different populations than the population studied.
5. *Publication bias*: downgrading the certainty of the evidence when there was evidence of selective reporting of outcomes based on the direction or strength of the effect or their statistical significance (i.e., if the Egger’s test *p* value was <.05 or examination of funnel plots indicated publication bias).

In line with the GRADE approach, we refer to the level of evidence as “certainty.”

### Subgroup Analysis

To assess whether different intervention types had different effects on the primary and secondary outcomes, we categorized interventions into three distinct types before conducting any analysis. This categorization was based on discussions among our research team members, including many allied health professionals.

- Type 1 interventions consisted of setting up a transitional care plan in-hospital and referring patients to relevant community care (allied health) professionals;
- Type 2 interventions were similar to type 1 interventions. However, they comprised a case manager who coordinated hospital-to-home transitions by facilitating interactions between the hospital, patients and their families, and community care settings.
- Type 3 interventions were the most complex as they comprised a comprehensive recovery trajectory delivered by dedicated multidisciplinary teams from the hospital who took care of the entire postdischarge rehabilitation process through outpatient rehabilitation, home visits, and phone calls.

We hypothesized that more complex interventions (types 2 and 3) would affect readmissions and health-related outcomes more than simple interventions. Moreover, we performed sensitivity analyses excluding studies with a high risk of bias. The sensitivity analyses were not stratified for intervention type because the number of studies became too small to interpret the results.

To interpret the results of the subgroup and sensitivity analyses, we followed the GRADE guidance group’s latest recommendations (Guyatt et al., 2023). Briefly, we performed *Q* tests to check for possible subgroup effects (i.e., the relevance of conducting a subgroup analysis per intervention type or per risk of bias). If the *p* value for the *Q* test was .1 or higher, no further investigation of subgroup effects was conducted. When the *p* value was smaller than .1, we used the Instrument to assess the Credibility of Effect Modification Analyses (ICEMAN) to determine the credibility of the subgroup analysis. The ICEMAN instrument can be used to assess whether an effect modification exists with the help of nine criteria to judge the internal consistency (i.e., whether the results of the subgroup analysis are internally consistent with the overall study findings), the statistical power (i.e., whether the subgroup analysis has adequate statistical power to detect meaningful differences, considering the number of studies in each subgroup), and the methodological rigor of subgroup analyses (i.e., how the subgroups were defined, the appropriateness of statistical methods used, and how potential biases were addressed; Schandelmaier et al., 2020). For subgroup analyses, we use the term “credibility,” which refers to the degree of confidence that the observed effect modification reflects a true difference in effects across subgroups, rather than being the result of random variations or bias. ICEMAN’s final rating of the credibility of the subgroup effect ranges from “very low” to “high.” When the credibility of a subgroup effect was “very low” or “low,” we only reported the overall estimate and hence the subgroup effects were not reported. When the credibility of the subgroup analysis was “moderate” or “high,” we did report the subgroup effects from random effect meta-analyses using the GRADE approach.

## Results

**Figure 1.**
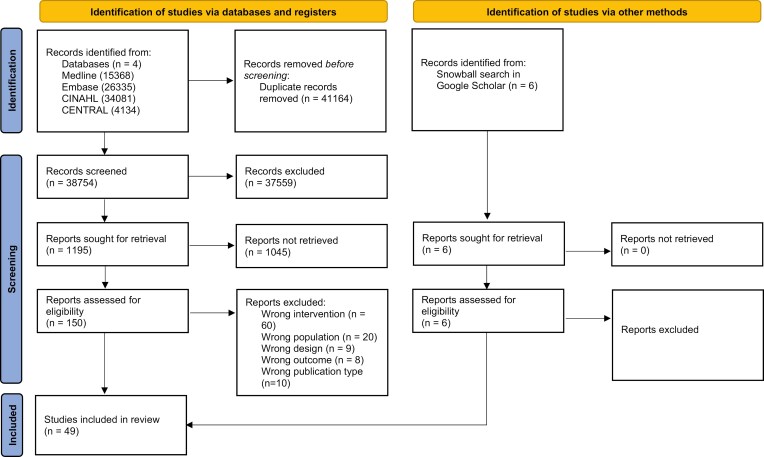
PRISMA flowchart.

After duplicate removal, titles and abstracts of the remaining records (*n* = 38,754) were screened for eligibility, leaving 158 studies meeting the inclusion criteria (Figure 1 PRISMA flowchart). Hundred-sixteen records were excluded based on full-text screening, and 43 records remained. Six additional papers were identified through a snowball search.

Forty-nine articles were included and critically appraised, of which 45 were RCTs (92%) and four cluster RCTs (8%). The characteristics of the included studies are detailed in Table 1, and summarized descriptions of the interventions per study are provided in Supplementary Appendix 2. The full references of the included studies are provided in Supplementary Appendix 6.

**Table 1.** Study Characteristics

First author, year^a^	Reference number	Country	Sample size	Age (mean, *SD*)	Gender F/M	Patient population/type of disease or condition	Average length of stay of index admission (mean, *SD* or median IQR^b^)	Community care professionals involved	Hospital professionals involved	Start and duration of the intervention	Outcomes measured	Type of intervention^c^
Allen, 2002	1	USA	93	I = 69.0 (12.0); C = 72.0 (10.0)	I = 27/20; C = 25/21	Neurological	NA	Physician	Nurse practitioner, physician, pharmacist, dietitian, social worker	3 to 7 days after discharge and up to 3 months	Physical performance and capacity, depression, health-related quality of life, readmission rate, mortality rate	2
Altfeld, 2013	2	USA	740	I = 74.1 (6.9); C = 75.0 (6.9); Overall = 74.5 (6.9)	NA	Older patients, not further specified	Overall = 4.9 (6.2); I = 5.0 (4.5); C = 4.9 (7.5)	Home health providers, not further specified, and physician	Social worker	Within 2 days after discharge and up to 1 month	Readmission rate, mortality rate	2
Atwood, 2022	3	Canada	3,710	I = 71.2 (10.5); C = 73.8 (11.7)	I = 313/291; C = 1,530/1,576	Pulmonary	I = 6.0 (4.0)^b^; C = 5.0 (5.0)^b^	Not specified	Nurse practitioner, respiratory therapist, physician, pharmacist, and allied health providers, not further specified	During hospitalization and up to 10 days after discharge	Readmission rate, emergency department visit rate	2
Baghaei, 2021	4	Iran	120	I = 57.3 (9.6); C = 55.4 (9.2)	I = 11/49; C = 18/42	Cardiovascular	NA	None	Nurse practitioner, physician	During hospitalization and up to 3 months after discharge	Anxiety, readmission rate	3
Balaban, 2008	5	USA	196	I = 58.0 (NA); C = 54.1 (NA); Historical C = 61.1 (NA)	I = 27/20; C = 30/19; Historical C = 60/40	Older patients, cardiovascular, pulmonary	I = 3.3 (NA); C = 3.1 (NA)	Primary care providers, not further specified	Nurse practitioner	During hospitalization and up to 1 month after discharge	Readmission rate, emergency department visit rate	2
Balaban, 2015	6	USA	1,510	I = 66.4 (15.5); C = 63.7 (16.7)	I = 323/262; C = 548/377	Older patients, cardiovascular, pulmonary	I = 3.0 (2.4); C = 3.5 (3.8)	Nurse practitioner, community services, not further specified	Patient navigator, not further specified	During hospitalization and up to 1 month after discharge	Readmission rate, emergency department visit rate	2
Balaban, 2017	7	USA	1,921	Age 60+ years: I = 74.5 (9.8); C = 75.1 (9.4) Age <60 years: I = 46.2 (9.6); C = 45.7 (10.8)	Age 60+ years: I = 315/230; C = 483/305 Age <60 years: I = 95/99; C = 205/189	Older patients, cardiovascular, pulmonary	I = 3.1 (2.5); C = 3.6 (4)	Primary care providers, not further specified	Patient navigator, not further specified	During hospitalization and up to 1 month after discharge	Readmission rate	2
Casas, 2006	8	Spain	155	I = 70.0 (9.0); C = 72.0 (9.0)	I = 15/50; C = 11/79	Pulmonary	I = 8.5 (5.5); C = 7.8 (4.2)	Physician, nurse practitioner, social worker	Nurse practitioner	During hospitalization and up to 9 months after discharge	Readmission rate, mortality rate	2
Coskun, 2022	9	Turkey	64	NA	I = 9/23; C = 5/27	Cardiovascular	NA	None	Physician, nurse practitioner, physiotherapist, dietitian	During hospitalization and up to 6 months after discharge	Physical performance, physical and mental quality of life, readmission rate, emergency department visit rate	3
Courtney, 2009	10	Australia	122	I = 78.1 (6.3); C = 79.4 (7.3); Overall = 78.8 (6.9)	I = 36/22; C = 40/24	Older patients, cardiovascular, pulmonary, gastrointestinal, trauma, and comorbidities	Overall = 4.6 (2.9); I = 4.6 (2.7); C = 4.7 (3.3)	None	Nurse practitioner, physiotherapist	During hospitalization and up to 6 months after discharge	Physical and mental quality of life, readmission rate	2
Davidson, 2010	11	Australia	105	I = 71.6 (NA); C = 73.9 (NA)	I = 20/33; C = 21/31	Cardiovascular	NA	None	Nurse practitioner, physiotherapist	At discharge and up to 3 months after discharge	Physical capacity, readmission rate, mortality rate	3
Del Sindaco, 2007	12	Italy	173	I = 77.4 (5.9); C = 77.5 (5.7)	I = 42/44; C = 41/46	Cardiovascular	NA	Physician	Physician, nurse practitioner	During hospitalization and up to 6 months after discharge	Readmission rate, mortality rate, health-related quality of life, physical capacity	2
Deng, 2021	13	China	84	I = 45.7 (13.9); C = 48.2 (7.3); Overall = 47.9 (12.6)	I = 12/30; C = 10/32	Limb disabilities	NA	Physician, physiotherapist, nurse practitioner, social worker	Physician	At discharge and up to 6 months after discharge	Physical performance, physical and mental quality of life	3
Dhalla, 2014	14	Canada	1,932	I = 71.2 (16.1); C = 71.3 (16.0)	I = 472/491; C = 465/495	Cardiovascular and other conditions not further specified	NA	Physician, community services (e.g., occupational therapy)	Physician, pharmacist, nurse practitioner, clerical assistant, coordinator	During hospitalization and up to 1 year after discharge	Physical performance, readmission rate, mortality rate, emergency department visit rate	2
Donnelly, 2004	15	UK	113	Median age: I = 68.0 C = 71.0	NA	Neurological	I = 42.0 (NA); C = 50.0 (NA)	Occupational therapist, physiotherapist, speech and language therapist, rehabilitation assistants	Nurse practitioner, coordinator	During hospitalization and up to 1 year after discharge	Health-related quality of life, physical and mental quality of life, patient satisfaction	3
Evangelista, 2023	16	Italy	802	I = 75 (13.6); C = 74.4 (13.9); Overall = 74.7 (13.7)	I = 175/213; C = 221/193	Cardiovascular, pulmonary, gastrointestinal, urological, musculoskeletal, endocrine, neoplasm, neurological	NA	Not specified	Multidisciplinary team, not further specified	During hospitalization only	Readmission rate, mortality rate, emergency department visit rate	1
Evans, 1993	17	USA	835	NA	I = 16/401; C = 25/393	Medical (mainly cardiovascular, pulmonary, gastrointestinal, musculoskeletal, neoplasm), and neurological	NA	Home healthcare and community agencies, not further specified	Social worker, physician, nurse practitioner	During hospitalization only	Readmission rate	1
Finkelstein, 2020	18	USA	782	NA	NA	Medically and socially complex patients, not further specified	NA	Community health workers, not further specified	Nurse practitioner, social worker	During hospitalization and up to 3 months after discharge	Readmission rate	2
Finlayson, 2018	19	Australia	222	Exercise I = 77.6 (6.5); Nurse I = 77.8 (6.2); Exercise + Nurse I = 77.1 (7.64); C = 77.9 (6.2)	Exercise I = 42/14; Nurse I = 37/17; Exercise + Nurse I = 46/11; C = 37/18	Older patients with cardiovascular, pulmonary, urological, and other nonspecified conditions	Overall = 5.0 (1.0–47.0)^b^; Exercise I = 6.8 (6.3); Nurse I = 5.1 (3.8); Exercise + Nurse I = 5.0 (1.0–13.0)^b^ C = 5.0 (1.0–34.0)	None	Exercise physiologist, nurse practitioner	During hospitalization and up to 6 months after discharge	Readmission rate	3
Fjaertoft, 2003	20	Norway	320	I = 74.0 (NA); C = 73.8 (NA)	I = 74/86; C = 90/70	Neurological	I = 18.6 (NA); C = 31.1 (NA)	In cooperation with the primary healthcare system, not further specified	Physiotherapist, occupational therapist, nurse practitioner, physician	During hospitalization and up to 1 month after discharge	Physical performance	3
Fjaertoft, 2011	21	Norway	320	I = 74.0 (NA); C = 73.8 (NA)	I = 74/86; C = 90/70	Neurological	NA	In cooperation with the primary healthcare system, not further specified	Physiotherapist, occupational therapist, nurse practitioner, physician	During hospitalization and up to 1 month after discharge	Physical performance, mortality	3
Garcia-Aymerich, 2007	22	Spain	62	I = 72.0 (10.0); C = 73.0 (9.0)	I = 4/17; C = 4/37	Pulmonary	NA	Physician, social worker, primary care team, not further specified	Nurse practitioner	During hospitalization and up to 9 months after discharge	Health-related quality of life	2
Hofstad, 2014	23	Norway	306	Group 1: I = 70.6 (NA); Group 2: I = 72.0 (NA); C = 74.2 (NA)	Group 1: I = 47/56 Group 2: I = 61/43 C = 47/52	Neurological	NA	Nurse practitioner, physiotherapist, occupational therapist	Nurse practitioner, physiotherapist, occupational therapist, physician	During hospitalization and up to 6 months after discharge	Physical performance and capacity, patient satisfaction	3
Hu, 2020	24	China	220	I = 33.0 (7.7); C = 31.8 (9.2)	I = 30/70; C = 40/58	Urological	NA	None	Nurse practitioner and chronic disease management specialist, not further specified	During hospitalization and up to 1 month after discharge	Readmission rate, patient satisfaction, emergency department visit rate	2
Hung, 2023	25	Taiwan	96	I = 43.4 (12.9); C = 41.1 (14.1)	I = 22/21; C = 24/18	Trauma	I = 7.2 (4.6); C = 8.1 (5.6)	None	Nurse practitioner, physician, rehabilitation therapist	During hospitalization and up to 14 days after discharge	Health-related quality of life	2
Jack, 200	26	USA	749	I = 50.1 (15.1); C = 49.6 (15.3)	I = 178/195; C = 200/176	General medical, not further specified	I = 2.8 (3.4); C = 2.6 (3.0)	Physicians	Nurse practitioner, pharmacist	During hospitalization and up to 1 month after discharge	Readmission rate, emergency department visit rate	2
Jackson, 2012	27	USA	15	NA	I = 5/2; C = 5/3	Intensive care	I = 11.0 (6.2–13.0)^b^; C = 11.5 (9.2–14.4)^b^	None	Psychology technician, psychologist, exercise physiologist, social worker, occupational therapist	During hospitalization and up to 3 months after discharge	Physical capacity and performance, cognitive functioning	3
Jepma, 2021	28	Netherlands	306	I = 82.5 (6.1); C = 82.3 (6.5)	I = 83/70; C = 67/86	Older cardiovascular	I = 7.0 (4.0–10.0)^b^; C = 7.0 (4.5–10.0^b^	Nurse practitioner, physiotherapist	Nurse practitioner, physician, other disciplines not further specified	During hospitalization and up to 6 weeks after discharge	Readmission rate, mortality rate	3
Ko, 2017	29	China	180	I = 74.9 (7.9); C = 74.6 (8.6)	I = 5/85; C = 3/87	Pulmonary	NA	None	Nurse practitioner, physiotherapist, physician	During hospitalization and up to 1 year after discharge	Physical capacity, readmission rate	3
Lainscak, 2013	30	Slovenia	253	I = 71.0 (9.0); C = 71.0 (9.0)	I = 37/81; C = 34/101	Pulmonary	NA	Nurse practitioner, physician, physiotherapist, social worker, and other providers not further specified	Discharge coordinator	During hospitalization and up to 10 days after discharge	Readmission rate, mortality rate	2
Lanzeta, 2016	31	Spain	140	Overall = 78.2 (NA)	I = 20/50; C = 25/45	Older patients with multimorbidity, not further specified	NA	Physician, nurse practitioner	Physician, nurse practitioner	During hospitalization and up to 1 year after discharge	Readmission rate, emergency department visit rate	2
Latour, 2006	32	Netherlands	147	I = 65.3 (15.7); C = 62.3 (17.5)	I = 39/39; C = 33/36	Cardiovascular, pulmonary, gastrointestinal, endocrine, infectious diseases, and other conditions not specified	I = 10.1 (7.0); C = 13.3 (15.99)	Physician, allied health professionals	Nurse practitioner, physician	Within 5 days after discharge and up to 6 months	Physical and mental quality of life, readmission rate	2
Lim, 2003	33	Australia	598	I = 76.5 (7.0); C = 76.8 (6.0)	I = 187/124; C = 164/123	Older patients (medical and surgical, not further specified)	I = 10.0 (8.8–11.1)^b^; C = 10.4 (9.5–11.4)^b^	Community services, not further specified	Nurse practitioner, allied health professionals not further specified	During hospitalization and after discharge, not further specified	Readmission rate, emergency department visit rate	2
Linden, 2014	34	USA	512	I = 65.8 (12.1); C = 67.7 (11.8)	I = 142/111; C = 153/106	Cardiovascular and pulmonary	I = 5.1 (4.2); C = 5.4 (3.9)	Physician	Nurse practitioner	During hospitalization and up to 3 months after discharge	Readmission rate, mortality rate	2
Liu, 2023	35	China	100	I = 81.6 (6.9); C = 81.9 (7.4)	I = 26/24; C = 19/31	Older patients with comorbidities, not further specified	NA	None	Nurse practitioner, physician, pharmacist, psychologist, dietitian, rehabilitation therapist	During hospitalization and up to 6 months after discharge	Physical performance, readmission rate	2
Markle-Reid, 2021	36	Canada	99	NA	I = 29/18; C = 33/19	Older patients with multimorbidity, not further specified	NA	Primary care health and social services providers, not further specified	Nurse practitioner	During hospitalization and up to 6 months after discharge	Depression, anxiety, physical, and mental quality of life	2
Markle-Reid, 2023	37	Canada	90	I = 68.7 (9.5); C = 70.9 (9.3)	I = 19/25; C = 17/29	Older patients with stroke and multimorbidity	NA	None	Nurse practitioner, physiotherapist, occupational therapist	During hospitalization and up to 6 months after discharge	Depression, physical and mental quality of life, readmission rate	3
McCorkle, 2000	38	USA	375	NA	I = 92/98; C = 103/82	Surgical oncology	I = 10.8 (10.4); C = 9.6 (9.0)	Healthcare settings and providers, not further specified	Nurse practitioner	Within 1 day after discharge and up to 1 month	Physical performance, depression, mortality rate	2
Meyer, 2022	39	Germany	104	NA	I = 30/24; C = 29/21	Older patients with multimorbidity, not further specified	I = 20.2 (13.6); C = 19.4 (10.5)	Social worker	Physician, nurse practitioner, dietitian, psychologist, therapist, not further specified	During hospitalization and up to 6 months after discharge	Depression, readmission rate, mortality rate	1
Naylor, 2004	40	USA	239	I = 76.4 (6.9); C = 75.6 (6.5)	I = 71/47; C = 66/55	Older cardiovascular	NA	Physician and other providers, not further specified	Physician, nurse practitioner, pharmacist, dietitian, social worker, physiotherapist	During hospitalization and up to 3 months after discharge	Health-related quality of life, physical and mental quality of life, readmission rate, patient satisfaction	2
Preen, 2005	41	Australia	189	I = 74.8 (6.7); C = 75.4 (7.9); Overall = 75.1 (10.9)	I = 57/34; C = 58/40	Pulmonary	I = 11.6 (5.7); C = 2.4 (7.4)	Physician, community service providers, not further specified	Nurse practitioner	During hospitalization and up to 1 week after discharge	Physical and mental quality of life, patient satisfaction	1
Rich, 1993	42	USA	98	I = 80.0 (6.3); C = 77.3 (6.1)	I = 38/25; C = 20/15	Older cardiovascular	I = 10.5 (7.3); C = 9.6 (5.8)	None	Nurse practitioner, dietitian, physician, social worker	During hospitalization and up to 1 week after discharge	Readmission rate	2
Santana, 2017	43	Canada	1,399	I = 58.8 (17.9); C = 59.1 (17.9)	I = 324/377; C = 325/373	Pulmonary, infections, urological, cardiovascular, gastrointestinal, rheumatology, poison, metabolic, and other, not further specified	NA	Community-based providers not further specified	Physician, multidisciplinary team not further specified	During hospitalization for an unspecified time period	Readmission rate, mortality rate	1
Schnipper, 2021	44	USA	1,657	NA	I = 540/438; C = 364/315	Medical and surgical patients, not further specified	NA	Nurse practitioner	Nurse practitioner, physician assistant, pharmacist	During hospitalization and up to 1 month after discharge	Readmission rate	2
Thorsen, 2005	45	Sweden	54	I = 71.0 (NA); C = 71.0 (NA)	I = 15/15; C = 10/14	Neurological	I = 14.0 (NA); C = 30 (NA)	None	Occupational therapist, physiotherapist, speech and language therapist	After discharge and up to 2 weeks	Physical capacity, cognitive functioning	3
Thygesen, 2015	46	Denmark	531	I = 77.8 (7.9); C = 77.7 (7.2)	I = 131/139; C = 124/137	Older patients with comorbidities, not further specified	Overall = 5.0 (3.0–11.0); I = 5.0 (3.0–10.0)^b^; C = 6.0 (3.0–12.0)^b^	Nurse practitioner, physician	Physician	During hospitalization and up to 2 months after discharge	Readmission rate	2
Van Spall, 2019	47	Canada	2,494	I = 77.8 (12.4); C = 77.6 (11.9)	I = 544/560; C = 714/676	Cardiovascular	I = 6.0 (4.0–10.0)^b^; C = 6.0 (4.0–10.0)^b^	Nurse practitioner, physician, multidisciplinary referrals, not further specified	Nurse practitioner	During hospitalization and up to 6 weeks after discharge	Readmission rate, mortality rate, emergency department visit rate	2
Visperas, 2021	48	USA	514	I = 66.0 (9.0); C = 65.0 (10.0)	I = 102/93; C = 113/91	Orthopedic	I = 1.5 (0.8); C = 1.4 (0.8)	None	Physician assistant, nurse practitioner, not further specified	During hospitalization and up to 3 months after discharge	Readmission rate, patient satisfaction	2
Zimmerman, 2021	49	USA	40	NA	I = 10/9; C = 8/13	Neurological	I = 5.9 (4.0–9.6)^b^; C = 4.0 (2.0–10.4)^b^	Not specified	Nurse practitioner, physician, physiotherapist, occupational therapist, speech and language therapist	During hospitalization	Patient satisfaction, emergency department visit rate, patient satisfaction	2

*Notes*: C = control group; F = female; I = intervention group; IQR = interquartile range; M = male; NA = not available; *SD* = standard deviation; USA = United States of America; UK = United Kingdom.

^a^The full references of the included studies are provided in Supplementary Appendix 6.

^b^Estimate reported as median and interquartile range.

^c^Interventions were organized into three categories according to their content. 1: Development of a care plan and referral to community care providers; 2: Similar interventions to category 1 with, in addition, a case manager coordinating patient care within and between settings; 3: Entire transitional care provided by a dedicated multidisciplinary team in-hospital and post-discharge.

### Study Characteristics

The included studies were published between 1993 and 2023 and were conducted in 16 countries, the majority of which were located in Northern America (*n* = 22, 45%), Europe (*n* = 15, 31%), and Oceania (*n* = 5, 10%). The total sample contained 25,566 participants, of whom 46% (*n* = 11,662) were female, 48% (*n* = 12,269) were male, and 6% (*n* = 1,635) were unspecified. Patients were generally 65 years and older (32 studies), and their primary diagnoses during admission varied, the majority being related to cardiovascular (18 studies), pulmonary (16 studies), and neurological (10 studies) conditions (Table 1).

**Figure 2.**
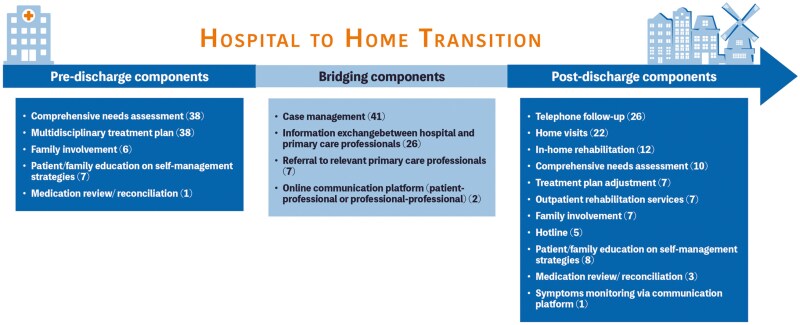
Components of the transitional care interventions per discharge phase.

Regarding the types of interventions, five studies (10%) assessed type 1 interventions (consisting of a care plan and referrals to relevant community care professionals), 30 studies (61%) assessed type 2 interventions (comprising a case manager coordinating care within and between settings), and 14 studies (29%) assessed type 3 interventions (offering an entire recovery trajectory delivered by dedicated multidisciplinary teams). Most interventions were coordinated by hospital nurse practitioners (23 studies, 47%) or by a hospital multidisciplinary team comprising medical, nursing, and allied health professionals (11 studies, 22%; Table 1; Figure 2; Supplementary Appendix 2).

### Risk of Bias and Certainty of the Evidence

**Figure 3.**
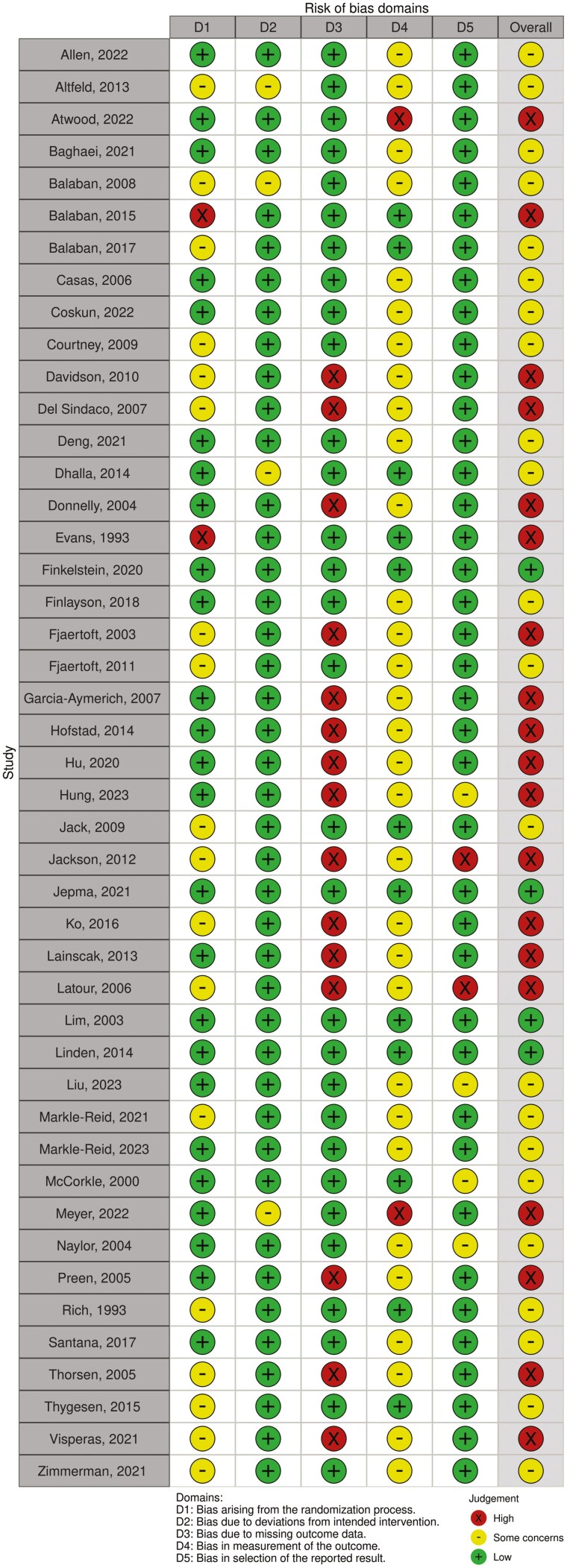
Risk of bias assessment with the Cochrane tool (RoB2)—randomized controlled trials.

**Figure 4.**
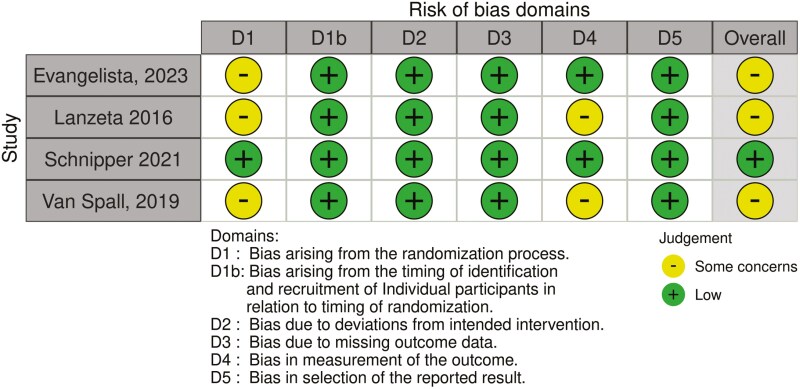
Risk of bias assessment with the Cochrane tool (RoB2)—cluster randomized controlled trials.

Four RCTs and one cluster RCT (10%) were assessed as “low risk,” 22 RCTs and three cluster RCTs (51%) as “some concerns,” and 19 RCTs (39%) as “high risk.” Generally, studies were assessed as “high risk” because outcome data were missing for more than 5% of the participants, and/or they did not report appropriate statistical or sensitivity analyses to correct for bias introduced by these missing data. In addition, due to the nature of the interventions and the outcomes measured (patient-reported outcomes), which are difficult to blind to patients, assessors, and people delivering the interventions, many studies were assessed as “some concerns” with regards to the second domain of the RoB2 tool, leading to a low number of papers assessed as low risk of bias (Figures 3 and 4). The certainty of evidence across all comparisons was graded as very low to high. Reasons for downgrading the certainty of evidence scores included studies rated as high risk of bias, high heterogeneity observed, wide confidence intervals, and potential for publication bias (Table 2).

**Table 2.** Evidence Quality (GRADE and ICEMAN Assessment)

Outcome	# Studies	*N*	Effect (95% CI)	Heterogeneity (*I*^2^)	Reason(s) for downgrading	Certainty of the evidence	*p*_subgroup_ ^$^	ICEMAN subgroup credibility^**^
Hospital readmission rates								
Overall	34	20,954	RR = 0.88 (0.80; 0.96)^*^	56%	Serious inconsistency, publication bias	Low		
Subgroup risk of bias							.564	
Subgroup type							.062^$^	Low^**^
Mortality rates								
Overall	15	9,773	RR = 0.92 (0.84; 1.01)	27%	—	High		
Subgroup risk of bias							.582	
Subgroup type							.376	
Emergency department visit rates								
Overall	11	11,853	RR = 0.93 (0.57; 1.51)	98%	Serious inconsistency, serious imprecision	Very low		
Subgroup risk of bias							.320	
Subgroup type							.244	
Health-related quality of life								
Overall	3	355	SMD = 0.21 (−0.49; 0.92)	99%	Serious inconsistency, very serious imprecision	Very low		
Subgroup risk of bias							.000^$^	Low^**^
Subgroup type							.000^$^	Low^**^
Physical quality of life								
Overall	9	1,050	SMD = 0.54 (−0.06; 1.15)	100%	Serious inconsistency	Moderate		
Subgroup risk of bias							.008^$^	Low^**^
Subgroup type							.798	
Mental quality of life								
Overall	9	1,050	SMD = 0.44 (−0.08; 0.96)	100%	Serious inconsistency	Moderate		
Subgroup risk of bias							.073^$^	Low^**^
Subgroup type							.961	
Depression								
Overall	5	693	SMD = −0.06 (−0.25; 0.13)	99%	Serious inconsistency, very serious imprecision	Very low		
Subgroup risk of bias							.000^$^	Low^**^
Subgroup type							.000^$^	Low^**^
Anxiety								
Overall	2	219	SMD = 0.43 (−2.06; 2.92)	100%	Serious inconsistency, very serious imprecision	Very low		
Physical performance								
Overall	8	1,163	SMD = 0.49 (−0.11; 1.10)	89%	Serious inconsistency, publication bias	Low		
Subgroup risk of bias							.741	
Subgroup type							.046^$^	Moderate
Type 2 interventions	3	581	SMD = −0.08 (−0.63; 0.47)	43%	Very serious imprecision, publication bias	Very low		
Type 3 interventions	5	582	SMD = 0.83 (0.02; 1.65)^*^	85%	Serious inconsistency, publication bias	Low		
Physical capacity								
Overall	4	329	SMD = 0.59 (−0.06; 1.24)	71%	Serious inconsistency, serious risk of bias	Low		
Subgroup risk of bias	All studies were assessed as high risk							
Subgroup type	All studies were of type 3							
Patient satisfaction								
Overall	5	1,171	SMD = 0.49 (−0.14; 1.12)	92%	Serious inconsistency, serious risk of bias	Low		
Subgroup risk of bias							.980	
Subgroup type							.621	

*Notes*: CI = confidence interval; GRADE = XXX; ICEMAN = XXX; *N* = number of participants; RR = relative risk; SMD = standardized mean difference.

^*^Statistically significant.

^**^If the credibility of the subgroup effect was very low or low, only the overall estimate was reported.

^$^If the *p* value of the interaction test (*Q* test) was .1 or higher, no further exploration of subgroup effect was conducted.

### Results of the Meta-Analyses and Narrative Syntheses

Supplementary Appendix 3 provides all forest and funnel plots for each comparison, including subgroup analyses. Supplementary Appendix 4 provides the complete ICEMAN assessments of subgroup effects credibility.

#### Readmission rate

Thirty-four studies involving 20,954 participants assessed readmission rate as an outcome (references 1, 2, 3, 4, 5, 6, 8, 9, 10, 11, 12, 14, 16, 17, 18, 19, 24, 26, 28, 29, 30, 31, 32, 33, 35, 37, 39, 40, 42, 43, 44, 46, 47, 48 in Table 1). There was “low” certainty that multidisciplinary transitional care interventions decreased the risk of all-cause hospital readmissions (relative risk [RR] = 0.88; 95% confidence intervals [95% CI] = 0.80 to 0.96) compared with usual care (Table 2) due to *inconsistency* (*I*² = 56%) and possible *publication bias* (Egger *p* = .001, Supplementary Appendix 3). Because the heterogeneity test suggested moderate heterogeneity across studies, the results presented are those of the random effect model.

One study could not be pooled for meta-analysis (reference 34 in Table 1). This study found no difference in readmission rate between intervention and usual care.

#### Mortality rate

Across 15 studies (references 1, 2, 8, 11, 12, 14, 16, 21, 28, 30, 34, 38, 39, 43, 47 in Table 1), including 9,773 participants, there was “high” certainty that multidisciplinary transitional care interventions were associated with a mortality reduction (RR = 0.92; 95% CI = 0.84 to 1.01; *I*² = 27%; Table 2 and Supplementary Appendix 3). There was no indication of *publication bias* (Egger *p* = .101, Supplementary Appendix 3).

#### Emergency department visit rate

Pooling data from 11 studies (references 3, 5, 6, 9, 14, 24, 26, 31, 33, 47, 48 in Table 1) including 11,853 participants, we found “very low” certainty that multidisciplinary transitional care interventions were associated with an emergency department visits reduction (RR = 0.93; 95% CI = 0.57 to 1.51; *I*² = 98%) compared to usual care due to *inconsistency* very serious *imprecision* (Table 2 and Supplementary Appendix 3). There was no indication of *publication bias* (Egger *p* = .419, Supplementary Appendix 3).

One study could not be pooled for meta-analysis (reference 34 in Table 1). This study found no difference in emergency department visit rate between intervention and usual care.

#### Health-related quality of life

Health-related quality of life measurements obtained with several self-reported questionnaires were pooled from three studies (references 1, 15, 40 in Table 1) including 355 participants. All self-reported questionnaires used in the studies are reported in Supplementary Appendix 5.

There was “very low” certainty that multidisciplinary transitional care interventions increased health-related quality of life due to serious *inconsistency* and very serious *imprecision* (standardized mean difference [SMD] = 0.21; 95% CI = −0.49 to 0.92, *I*² = 99%; Table 2 and Supplementary Appendix 3). There was no publication bias (Egger *p* = .911, Supplementary Appendix 3).

Three studies (references 12, 22, 25 in Table 1) could not be pooled for meta-analysis. One of these studies reported significant improvements regarding health-related quality of life in the intervention group compared to usual care (reference 12 in Table 1). In contrast, the other two studies reported no difference (references 22, 25 in Table 1).

#### Physical and mental quality of life

Physical and mental quality of life were measured postintervention in nine studies (references 9, 10, 13, 15, 32, 36, 37, 40, 41 in Table 1), involving 1,050 participants, through self-reported questionnaires (Supplementary Appendix 5).

There was “moderate” certainty that multidisciplinary transitional care interventions increased physical quality of life due to *inconsistency* and *imprecision* (SMD = 0.54; 95% CI = −0.06 to 1.15, *I*² = 100%; Table 2 and Supplementary Appendix 3). There was no publication bias (Egger *p* = .285, Supplementary Appendix 3).

There was “moderate” certainty that multidisciplinary transitional care interventions substantially increased mental quality of life due to *inconsistency* (SMD = 0.44; 95% CI = −0.08 to 0.96, *I*² = 100%; Table 2 and Supplementary Appendix 3). There was no publication bias (Egger *p* = .497, Supplementary Appendix 3).

#### Psychological distress

Five studies (references 1, 36, 37, 38, 39 in Table 1), including 693 participants, provided “very low” certainty evidence that multidisciplinary transitional care interventions were associated with a higher depression score compared with usual care (SMD = −0.06; 95% CI = −0.25 to 0.13, *I*² = 99%) due to *inconsistency* and very serious *imprecision* (Table 2 and Supplementary Appendix 3). There was no publication bias (Egger *p* = .485, Supplementary Appendix 3).

Two studies (references 4, 36 in Table 1), including 219 participants, provided “very low” certainty evidence that multidisciplinary transitional care interventions increased anxiety due to *inconsistency* and very serious *imprecision* (SMD = 0.43; 95% CI = −2.06 to 2.92, *I*² = 99%; Table 2 and Supplementary Appendix 3).

#### Physical performance and capacity

Physical performance was measured postintervention in eight studies (references 9, 13, 15, 23, 27, 35, 38, 39 in Table 1), involving 1,163 participants, through self-reported questionnaires (Supplementary Appendix 5). Physical capacity was measured in four studies (references 11, 27, 29, 45 in Table 1), involving 423 participants.

There was “low” certainty that multidisciplinary transitional care interventions increased physical performance (SMD = 0.49; 95% CI = −0.11 to 1.10, *I*² = 89%) due to *inconsistency* and *publication bias* (Egger *p* = .029, Table 2, and Supplementary Appendix 3). Furthermore, there was “low” certainty that multidisciplinary transitional care interventions increased physical capacity (SMD = 0.59; 95% CI = −0.06 to 1.24, *I*² = 71%) due to *inconsistency* and *risk of bias* (all studies were assessed as high risk; Table 2 and Supplementary Appendix 3).

Five studies could not be pooled for meta-analysis (references 1, 12, 20, 21, 23 in Table 1). These studies found an effect favoring the intervention group compared to usual care.

#### Cognitive functioning

Cognitive functioning was measured in two studies (references 27, 45 in Table 1), but could not be pooled for meta-analysis. Of them, Jackson et al. (reference 27 in Table 1) reported improvements at follow-up, whereas similar median follow-up measurements were observed by Thorsen et al. (reference 45 in Table 1) in the intervention group compared to usual care.

#### Patient satisfaction

Pooling data from five studies (references 15, 23, 24, 40, 48 in Table 1) involving 1,171 participants, there was “low” certainty that multidisciplinary transitional care interventions increased patient satisfaction due to *inconsistency* and *risk of bias* (SMD = 0.49; 95% CI = −0.14 to 1.12, *I*² = 92%; Table 2 and Supplementary Appendix 3).

The results of two studies (references 41, 49 in Table 1) could not be pooled for meta-analysis. Both studies found greater satisfaction with the discharge process in the intervention group compared to usual care.

### Sensitivity and Subgroup Analyses

For each outcome, we performed a sensitivity analysis by excluding high-risk-of-bias studies. Additionally, we performed subgroup analyses to investigate whether different intervention types had different effects on readmissions and health-related outcomes. These intervention types were defined as follows: (1) transitional care plans with referrals to relevant primary care professionals, (2) case-managed interventions overseeing the entire transition process, and (3) more complex interventions led by a multidisciplinary team, including comprehensive recovery pathways, outpatient rehabilitation, and home visits.

We do not report the sensitivity and subgroup analysis results for readmission rate, mortality rate, emergency department visit rate, health-related quality of life, physical and mental quality of life, psychological distress, physical capacity, or patient satisfaction. This decision was based on GRADE recommendations: i.e., sensitivity and subgroup analyses ought not to be performed if either the *Q* test *p* values exceed .1, indicating no significant differences between subgroups and overall effects, or the ICEMAN credibility rating for the subgroup analyses was “low” or “very low.” Please note that a “low” or “very low” credibility rating was generally assigned when at least one subgroup included fewer than three studies, a threshold considered insufficient for drawing reliable conclusions.

#### Physical performance

The ICEMAN credibility of the subgroup analysis per intervention type for physical performance was “moderate” (Supplementary Appendix 4), and hence a subgroup analysis was performed. This analysis revealed “low” certainty (GRADE)—due to inconsistency and potential publication bias—that the magnitude of the effect was larger than the overall effect and statistically significant only for type 3 interventions compared with usual care (SMD = 0.83; 95% CI = 0.02 to 1.65, *I*² = 85%; Table 2 and Supplementary Appendix 3).

## Discussion and Implications

This systematic review and meta-analysis aimed to identify, critically appraise, and synthesize the current body of RCTs investigating the impact of multidisciplinary transitional care interventions addressing patients’ complex care needs during hospital-to-home transitions on hospital readmissions and health-related outcomes. We found that multidisciplinary transitional care interventions were associated with a reduction in hospital readmissions. However, the certainty of the evidence was low due to inconsistency across studies and possible publication bias. Furthermore, there was “high” certainty of an overall relative reduction in mortality, and multidisciplinary transitional care interventions were associated with increased physical (“moderate” certainty) and mental quality of life (“moderate” certainty). There was also evidence of improved physical performance, particularly with type 3 interventions, and there was an overall trend toward improved physical capacity and patient satisfaction.

Several systematic reviews examined the effects of mono- and multidisciplinary transitional care interventions aimed at improving communication between healthcare professionals and (older) patients with cardiovascular or general medical conditions or post-surgery (Doss & Popejoy, 2023; Facchinetti et al., 2020; Li, Li, et al., 2021; Li, Fu, et al., 2021). These studies showed relative risk reductions for mortality and readmissions of up to 18% and 22%, respectively, consistent with our findings, although our results led to somewhat smaller effect magnitudes. Moreover, their results regarding emergency department visits were—similar to our results—inconclusive. These studies, however, did not assess other outcomes, such as physical, cognitive, and psychosocial status. Furthermore, the interventions evaluated in these reviews were often monodisciplinary, consisting only of a nurse practitioner in charge of patient follow-up via telephone, digital applications, home visits, or outpatient clinic visits. This makes their findings difficult to compare with ours because we focused on multidisciplinary interventions specifically.

Tyler et al. (2023) recently conducted a comprehensive review of mono- and multidisciplinary transitional care interventions involving 97,408 participants with a wide range of conditions or diseases from 126 RCTs conducted worldwide. They investigated whether the effectiveness of transitional care interventions differed depending on the number of components they contained. They found that interventions with fewer components were associated with relative reductions in hospital readmissions between 18% and 55% compared with usual care, while this was less pronounced in more complex interventions. An explanation for this might be that interventions with fewer components are easier to implement and execute. On the other hand, interventions with more components were better for improving patient satisfaction. However, unlike in our meta-analysis, the interventions they evaluated did not always address patients’ physical, cognitive, and/or psychosocial needs but often only focused on improving medication adherence. All interventions included in our analysis were complex by nature, as they involved multidisciplinary approaches. Thus, we did not perform subgroup analyses based on complexity; instead, we categorized interventions by content to explore whether different types of interventions had different effects on readmissions and health-related outcomes. For most comparisons, we found no significant differences between intervention types. However, for physical performance, type 3 interventions (i.e., led by a multidisciplinary team and including comprehensive recovery pathways, outpatient rehabilitation, and home visits) appeared more effective than type 2 interventions (i.e., case-managed interventions overseeing the entire transition process), though the certainty of the evidence was “low” due to inconsistency and potential publication bias. This finding might be explained by differences in patient populations: type 3 interventions may have been more frequently provided to patients with more complex care needs, who thus had greater potential for improvement in physical performance than those receiving type 2 interventions. In this respect, our findings build on those of Tyler et al. (2023) by providing a more specific understanding of complex, multidisciplinary transitional care interventions.

Despite the positive outlook of our findings regarding readmissions and mortality reduction and improving health-related outcomes, we observed severe heterogeneity in the effect sizes across studies, suggesting that some multidisciplinary transitional care interventions are more effective than others. This heterogeneity can be partially explained by the variability in the interventions’ content and how the interventions were provided, which we could not assess because of a lack of statistical power. In a recent comprehensive meta-synthesis, including 53 qualitative studies conducted globally, we examined the experiences and needs of patients, family members, and healthcare professionals during hospital-to-home transitions (van Grootel, Collet, van Dongen, et al., 2024). We found that experiences and needs vary widely from one patient to another and that there is a crucial need for more tailored support and information provision. Future research should clarify how to tailor multidisciplinary transitional care interventions to specific environments, settings, and populations to optimize their impact on healthcare utilization and health outcomes.

### Practical Implications

From a practical perspective, our findings suggest that practitioners should not hesitate to implement multidisciplinary transitional care interventions despite the “low” certainty of evidence in some comparisons. This recommendation is supported by the overall trend across studies, which consistently shows that these interventions lead to improved patient outcomes, including reduced hospital readmissions and mortality, as well as enhanced quality of life, physical performance, and patient satisfaction. However, given the heterogeneity in effect sizes, it is crucial for practitioners and healthcare organizations to adopt a strategic approach to implementation. We recommend tailoring multidisciplinary transitional care interventions to the specific needs of the patient population, healthcare setting, and available resources to maximize their impact. The current evidence does not identify a universally optimal intervention type for all settings. For example, the most complex interventions (type 3), which involve intensive follow-up care, may be particularly effective in improving health-related outcomes in regions with well-established primary care networks. Conversely, their effectiveness may be limited in regions where such networks are underdeveloped, necessitating alternative strategies to ensure successful implementation and sustainability.

In another systematic review, we identified key factors that facilitate or hinder the successful implementation of multidisciplinary transitional care interventions (Collet et al., 2025). The main barriers included inadequate communication between stakeholders, workforce shortages, and financial constraints. Careful consideration of these factors is essential when designing and implementing multidisciplinary transitional care interventions. For example, if communication gaps between stakeholders are a barrier, standardized protocols or digital health solutions—such as shared electronic medical records and telehealth platforms—could enhance coordination between hospital and primary care providers. If financial constraints pose challenges, alternative financing models—such as bundled payments or value-based reimbursement—might be solutions worth exploring further. Future research should investigate the effectiveness of these tailored approaches to ensure that multidisciplinary transitional care interventions are not only successfully implemented but also sustainably integrated across healthcare settings.

### Strengths and Limitations

This systematic review contributes to the current knowledge about the effectiveness of multidisciplinary transitional care interventions on hospital readmission and health-related outcomes. It included an extensive systematic search, a thorough meta-analysis, and we identified many studies, increasing the reliability of the findings. However, some limitations to our study can be identified. First, due to the large number of researchers participating in the selection process, uniformity in article selection may have been negatively affected. To minimize this selection bias, we used sample checks and held meetings during the screening phase to monitor and guide the process. Second, substantial heterogeneity was observed across studies, possibly due to the variability of the interventions provided by participants. We minimized this issue by grouping interventions into three types and performing subgroup analyses. The duration of the interventions also varied widely, from a few days up to a year after discharge, making it difficult to compare the interventions. Despite this variability in intervention content, we pooled the data to allow for broader trend identification, and we employed random effects models to generate more generalizable conclusions. Furthermore, multidisciplinary transitional care interventions may affect more vulnerable populations, such as frail older people, differently than younger populations. The subgroup analysis we performed according to patient populations was inconclusive, as only a few trials investigated these populations. Finally, while the GRADE approach is a widely accepted framework for evaluating evidence certainty, it was originally designed for medical evidence synthesis—particularly for clinical guidelines, drug trials, and therapeutic interventions—rather than for assessing complex, multidisciplinary interventions implemented in real-world healthcare settings. As a result, the certainty ratings in our review may have been systematically downgraded due to high heterogeneity, without fully accounting for the inherent complexity of such interventions. Prior research suggests that meta-analytic studies for medical evidence synthesis typically exhibit lower methodological heterogeneity, resulting in less variable outcomes (Kepes et al., 2024). This raises concerns that heterogeneity assessment methodologies developed for medical clinical trials may not fully translate to complex interventions involving diverse healthcare professionals, patient populations, and healthcare settings. Future research should consider contextual moderators—such as healthcare system characteristics and variations in clinical practice (e.g., the presence of well-established primary care networks or differences in discharge planning model)—to better account for and mitigate heterogeneity. Additionally, the development or adaptation of evidence grading tools tailored specifically for complex interventions could improve assessment accuracy and enhance the applicability of findings across diverse healthcare contexts (Kepes et al., 2024).

## Conclusions

The findings of this systematic review and meta-analysis suggest that multidisciplinary transitional care interventions addressing patients’ complex care needs offer a viable solution to improve their quality of life and physical performance while reducing hospital readmissions and mortality. Given the varying effects observed across studies, we recommend tailoring strategies to implement such interventions to the environments, settings, and populations in which they are implemented to maximize their impact on health-related outcomes. A systematic and thorough needs assessment before implementing a transitional care intervention might improve its chances of success. However, further research is needed to determine how to tailor strategies to implement these interventions successfully.

## Supplementary Material

gnaf088_suppl_Supplementary_Appendixs

## Data Availability

All data supporting the findings of this study are available within the article and its supplementary material. The protocol was registered in PROSPERO (CRD42023421423).
