# Supplementary material for: The Impact of Multidisciplinary Transitional Care Interventions for Complex Care Needs: A Systematic Review and Meta-Analysis
Source: Gerontologist. 2025 Mar 5;65(6):gnaf088. doi: 10.1093/geront/gnaf088 (PMC12086065; doi:10.1093/geront/gnaf088)
Supplement: gnaf088_suppl_Supplementary_Appendixs [file gnaf088_suppl_supplementary_appendixs.docx]

Table of contents

[Appendix 1: Search strategy 3](#_Toc182173829)

[1. Search strings 3](#_Toc182173830)

[2. Excluded articles and reasons for exclusion 12](#_Toc182173831)

[Wrong intervention (e.g., non-multidisciplinary intervention) 12](#_Toc182173832)

[Wrong outcome (e.g., costs) 16](#_Toc182173833)

[Wrong population (e.g., patients visiting the emergency department and discharged home without hospital stay, patients discharged to a lonmg-term care facility) 17](#_Toc182173834)

[Wrong study design (e.g., protocol) 19](#_Toc182173835)

[Wrong publication type (e.g., conference abstract) 20](#_Toc182173836)

[Appendix 2: Summarized descriptions of the interventions per study 21](#_Toc182173837)

[Appendix 3: Meta-analyses per outcome: Forest plots, funnel plots, and subgroup analyses 29](#_Toc182173838)

[3. Hospital readmission rates (up to 2 years after hospital discharge) 29](#_Toc182173839)

[Forest plot overall analysis 29](#_Toc182173840)

[Funnel plot 30](#_Toc182173841)

[Subgroup analysis excluding high-risk-of-bias studies 31](#_Toc182173842)

[Subgroup analysis per intervention type 32](#_Toc182173843)

[4. Mortality rates (up to 44 months after hospital discharge) 33](#_Toc182173844)

[Forest plot overall analysis 33](#_Toc182173845)

[Funnel plot 34](#_Toc182173846)

[Subgroup analysis excluding high-risk-of-bias studies 35](#_Toc182173847)

[Subgroup analysis per intervention type 36](#_Toc182173848)

[5. Emergency department visit rates (up to 1 year after hospital discharge) 37](#_Toc182173849)

[Forest plot overall analysis 37](#_Toc182173850)

[Funnel plot 38](#_Toc182173851)

[Subgroup analysis excluding high-risk-of-bias studies 39](#_Toc182173852)

[Subgroup analysis per intervention type 40](#_Toc182173853)

[6. Health-related quality of life 41](#_Toc182173854)

[Forest plot overall analysis 41](#_Toc182173855)

[Funnel plot 41](#_Toc182173856)

[Subgroup analysis excluding high-risk-of-bias studies 42](#_Toc182173857)

[Subgroup analysis per intervention type: 42](#_Toc182173858)

[7. Physical quality of Life 43](#_Toc182173859)

[Forest plot overall analysis 43](#_Toc182173860)

[Funnel plot 43](#_Toc182173861)

[Subgroup analysis excluding high-risk-of-bias studies 44](#_Toc182173862)

[Subgroup analysis per intervention types 44](#_Toc182173863)

[8. Mental quality of life 45](#_Toc182173864)

[Forest plot overall analysis 45](#_Toc182173865)

[Funnel plot 45](#_Toc182173866)

[Subgroup analysis excluding high-risk-of-bias studies 46](#_Toc182173867)

[Subgroup analysis per intervention type 46](#_Toc182173868)

[9. Depression 47](#_Toc182173869)

[Forest plot overall analysis 47](#_Toc182173870)

[Funnel plot 47](#_Toc182173871)

[Subgroup analysis excluding high-risk-of-bias studies 48](#_Toc182173872)

[Subgroup analysis per intervention type 48](#_Toc182173873)

[10. Anxiety 49](#_Toc182173874)

[Forest plot overall analysis 49](#_Toc182173875)

[11. Physical performance 50](#_Toc182173876)

[Forest plot overall analysis 50](#_Toc182173877)

[Funnel plot 50](#_Toc182173878)

[Subgroup analysis excluding high-risk-of-bias studies 51](#_Toc182173879)

[Subgroup analysis per intervention type 51](#_Toc182173880)

[12. Physical capacity 52](#_Toc182173881)

[Forest plot overall analysis 52](#_Toc182173882)

[Funnel plot 52](#_Toc182173883)

[*13.* Patient satisfaction 53](#_Toc182173884)

[Forest plot overall analysis 53](#_Toc182173885)

[Funnel plot 53](#_Toc182173886)

[Subgroup analysis excluding high-risk-of-bias studies 54](#_Toc182173887)

[Subgroup analysis per intervention type 54](#_Toc182173888)

[Appendix 4: Subgroup effects credibility assessment (ICEMAN) 55](#_Toc182173889)

[Readmissions: Subgroup analysis per intervention type 55](#_Toc182173890)

[Physical quality of life: Subgroup analysis per risk of bias 57](#_Toc182173891)

[Depression: Subgroup analysis per risk of bias and per intervention type 59](#_Toc182173892)

[Physical performance: Subgroup analysis per intervention type 61](#_Toc182173893)

[Health-related quality of life: Subgroup analysis per intervention type 63](#_Toc182173894)

[Health-related quality of life: Subgroup analysis per risk of bias 65](#_Toc182173895)

[Appendix 5: Measurement instruments used in the studies 67](#_Toc182173896)

[Health-related quality of life 67](#_Toc182173897)

[Physical and mental quality of life 67](#_Toc182173898)

[Physical performance 67](#_Toc182173899)

[Physical capacity 67](#_Toc182173900)

[Appendix 6: References of the included studies 68](#_Toc182173901)

[References 72](#_Toc182173902)

# Appendix 1: Search strategy

## Search strings

Database: Embase Classic+Embase 1947 to 2024 June 19

| # | Searches | Results |
| --- | --- | --- |
| 1 | exp long term care/ or exp comorbidity/ or exp multiple chronic conditions/ | 2675948 |
| 2 | ((complex or "long term" or chronic or multidisciplinary) adj3 (care or treat* or therapy)).ti,ab,kf. | 422641 |
| 3 | ((complex or "high risk") adj3 (patient* or medically or adult*)).ti,ab,kf. | 211859 |
| 4 | (multiple adj3 (chronic adj3 (condition* or disease* or disorder* or ill* or patholog* or "health problem*"))).ti,ab,kf. | 4933 |
| 5 | (comorbidit* or "co morbidit*" or multimorbidit* or (("intercurrent" or concurrent) adj2 (illness* or disorder* or disease* or condition* or patholog* or "health problem*"))).ti,ab,kf. | 417983 |
| 6 | (polymorbidit* or Plurimorbidit* or ((multiple or polypathic) adj2 (condition* or disease* or disorder* or illness* or patholog* or "health problem*"))).ti,ab,kf. | 59410 |
| 7 | or/1-6 | 3314669 |
| 8 | exp clinical handover/ or exp patient care planning/ or exp case management/ | 54652 |
| 9 | ((transition* or discharge or postdischarge or handover or "follow up" or stepdown or "step down" or multidisciplinary) adj3 (care or treat* or therapy or coordinat*)).ti,ab,kf. | 165886 |
| 10 | ((plan* or admission or discharge or postdischarge) adj3 (coordinat* or contin* or manag* or process*)).ti,ab,kf. | 90116 |
| 11 | ((handover or handoff* or "hand over" or "hand off*" or signout* or "sign out*" or signover or "sign over") adj3 (patient* or plan* or coordinat* or manag* or program*)).ti,ab,kf. | 2080 |
| 12 | (care adj3 (coordinat* or plan* or manag* or goal* or contin* or process*)).ti,ab,kf. | 205192 |
| 13 | (case adj3 (manag* or plan* or coordinat* or program*)).ti,ab,kf. | 47902 |
| 14 | or/8-13 | 509018 |
| 15 | exp implementation science/ or exp system analysis/ or exp program evaluation/ or exp evaluation study/ | 122193 |
| 16 | ((system* or program* or process*) adj3 (analy* or integrat* or evaluat* or implement*)).ti,ab,kf. | 512795 |
| 17 | (implement* or evaluat* or coordinat* or strateg* or barrier* or block* or obstacle* or hinder* or constrain* or facilitat* or incentiv* or challenge* or enabler*).ti,ab,kf. | 11354020 |
| 18 | (Health adj3 (Knowledge or attitude* or practice*)).ti,ab,kf. | 66642 |
| 19 | or/15-18 | 11573529 |
| 20 | exp hospital readmission/ or exp mortality/ or exp "quality of life"/ or exp "cost"/ or exp self concept/ or exp evaluation study/ or exp outcome assessment/ or exp health care quality/ or exp questionnaire/ or *hospitalization/ | 6759382 |
| 21 | (rehospitalization or hospitali* or ((patient* or hospital* or "30 day" or "thirty day" or unplanned) adj3 (readmission* or re admission))).ti,ab,kf. | 610055 |
| 22 | (mortal* or ((death or fatality) adj2 (rate* or frequenc*))).ti,ab,kf. | 1570962 |
| 23 | (Self adj2 (Efficacy or concept)).ti,ab,kf. | 56565 |
| 24 | (Outcome adj3 (assess* or patient* or clinical* or "health Care")).ti,ab,kf. | 442769 |
| 25 | (Survey* or questionnaire* or cost*).ti,ab,kf. | 2860560 |
| 26 | (QOL or hrql or hrqol or (Quality adj2 (Healthcare or care or life))).ti,ab,kf. | 735130 |
| 27 | or/20-26 | 9030839 |
| 28 | exp adult/ or exp adult/ or exp geriatrics/ | 11342675 |
| 29 | (adult* or senior* or aged or elder* or geriatri* or ((old* or mature*) adj2 (people* or subject* or patient* or age* or men or male* or wom?n or female* or population* or cohort* or person*))).ti,ab,kf. | 4317440 |
| 30 | or/28-29 | 12799925 |
| 31 | 7 and 14 and 19 and 27 and 30 | 41247 |
| 32 | exp "review"/ | 3219946 |
| 33 | exp case report/ | 3015570 |
| 34 | ((exp animals/ or exp veterinary medicine/ or animal*.jw.) not exp humans/) or (experiment* model* or animal* or monkey* or sheep or ?ovine or lamb* or goat* or pig* or swine or porcine or pup* or dog* or canine or bitch* or beagle* or feline or rodent* or rabbit* or rat or rats or mouse or murine or mice).ti,kf. | 6720433 |
| 35 | ((Palliative or "end of life") adj3 care).ti,kf. | 51857 |
| 36 | exp conference abstract/ | 1929041 |
| 37 | or/32-36 | 14172981 |
| 38 | 31 not 37 | 25116 |

| Database(s): Ovid MEDLINE(R) ALL 1946 to June 19, 2024 | |  |
| --- | --- | --- |
|  | |  |
| # | Searches | Results |
| 1 | exp long term care/ or exp comorbidity/ or exp multimorbidity/ | 155159 |
| 2 | ((complex or "long term" or chronic or multidisciplinary) adj3 (care or treat* or therapy)).ti,ab,kf. | 286659 |
| 3 | ((complex or "high risk") adj3 (patient* or medically or adult*)).ti,ab,kf. | 123289 |
| 4 | (multiple adj3 (chronic adj3 (condition* or disease* or disorder* or ill* or patholog* or "health problem*"))).ti,ab,kf. | 3723 |
| 5 | (comorbidit* or "co morbidit*" or multimorbidit* or (("intercurrent" or concurrent) adj2 (illness* or disorder* or disease* or condition* or patholog* or "health problem*"))).ti,ab,kf. | 225274 |
| 6 | (polymorbidit* or Plurimorbidit* or ((multiple or polypathic) adj2 (condition* or disease* or disorder* or illness* or patholog* or "health problem*"))).ti,ab,kf. | 40223 |
| 7 | or/1-6 | 740161 |
| 8 | transitional care/ or exp "Continuity of Patient Care"/ or exp Patient Care Planning/ or exp Case Management/ | 351172 |
| 9 | ((transition* or discharge or postdischarge or handover or "follow up" or stepdown or "step down" or multidisciplinary) adj3 (care or treat* or therapy or coordinat*)).ti,ab,kf. | 99713 |
| 10 | ((plan* or admission or discharge or postdischarge) adj3 (coordinat* or contin* or manag* or process*)).ti,ab,kf. | 67126 |
| 11 | ((handover or handoff* or "hand over" or "hand off*" or signout* or "sign out*" or signover or "sign over") adj3 (patient* or plan* or coordinat* or manag* or program*)).ti,ab,kf. | 1233 |
| 12 | (care adj3 (coordinat* or plan* or manag* or goal* or contin* or process*)).ti,ab,kf. | 142285 |
| 13 | (case adj3 (manag* or plan* or coordinat* or program*)).ti,ab,kf. | 31803 |
| 14 | or/8-13 | 629459 |
| 15 | exp implementation science/ or exp Systems Analysis/ or exp Program Evaluation/ or exp Evaluation Study/ or exp Health Knowledge, Attitudes, Practice/ | 556392 |
| 16 | ((system* or program* or process*) adj3 (analy* or integrat* or evaluat* or implement*)).ti,ab,kf. | 390610 |
| 17 | (implement* or evaluat* or coordinat* or strateg* or barrier* or block* or obstacle* or hinder* or constrain* or facilitat* or incentiv* or challenge* or enabler*).ti,ab,kf. | 8429710 |
| 18 | (Health adj3 (Knowledge or attitude* or practice*)).ti,ab,kf. | 54539 |
| 19 | or/15-18 | 8838946 |
| 20 | exp Patient Readmission/ or exp Mortality/ or exp "Quality of Life"/ or exp "Costs and Cost Analysis"/ or exp Self Efficacy/ or exp Outcome Assessment, Health Care/ or exp "Quality of Health Care"/ or exp Quality Indicators, Health Care/ or exp "Surveys and Questionnaires"/ or *Hospitalization/ | 8459096 |
| 21 | (rehospitalization or hospitali* or ((patient* or hospital* or "30 day" or "thirty day" or unplanned) adj3 (readmission* or re admission))).ti,ab,kf. | 357319 |
| 22 | (mortal* or ((death or fatality) adj2 (rate* or frequenc*))).ti,ab,kf. | 1015372 |
| 23 | (Self adj2 (Efficacy or concept)).ti,ab,kf. | 47445 |
| 24 | (Outcome adj3 (assess* or patient* or clinical* or "health Care")).ti,ab,kf. | 268268 |
| 25 | (Survey* or questionnaire* or cost*).ti,ab,kf. | 2066841 |
| 26 | (QOL or hrql or hrqol or (Quality adj2 (Healthcare or care or life))).ti,ab,kf. | 464302 |
| 27 | or/20-25 | 9872105 |
| 28 | exp Adult/ or exp Aged/ or exp Geriatrics/ | 7956410 |
| 29 | (adult* or senior* or aged or elder* or geriatri* or ((old* or mature*) adj2 (people* or subject* or patient* or age* or men or male* or wom?n or female* or population* or cohort* or person*))).ti,ab,kf. | 2976839 |
| 30 | or/28-29 | 9256491 |
| 31 | 7 and 14 and 19 and 27 and 30 | 16860 |
| 32 | review.pt. | 3167658 |
| 33 | case reports.pt. | 2341453 |
| 34 | ((exp animals/ or exp veterinary medicine/ or animal*.jw.) not exp humans/) or (experiment* model* or animal* or monkey* or sheep or ?ovine or lamb* or goat* or pig* or swine or porcine or pup* or dog* or canine or bitch* or beagle* or feline or rodent* or rabbit* or rat or rats or mouse or murine or mice).ti,kf. | 5707397 |
| 35 | ((Palliative or "end of life") adj3 care).ti,kf. | 34095 |
| 36 | or/32-35 | 10840215 |
| 37 | 31 not 36 | 14616 |

Database - CINAHL Plus with Full Text June 20, 2024

| # | Query | Results |
| --- | --- | --- |
| S31 | (S28 or S29) AND (S27 AND S30) | 3,132 |
| S30 | S28 or S29 | 877,705 |
| S29 | TI ( (adult* or senior* or aged or elder* or geriatri* or ((old* or mature*) N2 (people* or subject* or patient* or age* or men or male* or wom?n or female* or population* or cohort* or person*))) ) OR AB ( (adult* or senior* or aged or elder* or geriatri* or ((old* or mature*) N2 (people* or subject* or patient* or age* or men or male* or wom?n or female* or population* or cohort* or person*))) ) | 875,267 |
| S28 | (MM "Adult") or (MM "Aged") or (MM "Geriatrics") | 7,346 |
| S27 | (S20 or S21 or S22 or S23 or S24 or S25) AND (S7 AND S14 AND S19 AND S26) | 9,912 |
| S26 | S20 or S21 or S22 or S23 or S24 or S25 | 1,373,938 |
| S25 | TI ( (QOL or hrql or hrqol or (Quality N2 (Healthcare or care or life))) ) OR AB ( (QOL or hrql or hrqol or (Quality N2 (Healthcare or care or life))) ) | 227,059 |
| S24 | TI ( (Survey* or questionnaire* or cost*) ) OR AB ( (Survey* or questionnaire* or cost*) ) | 758,189 |
| S23 | TI ( (Outcome N3 (assess* or patient* or clinical* or "health Care")) ) OR AB ( (Outcome N3 (assess* or patient* or clinical* or "health Care")) ) | 257,48 |
| S22 | TI ( (Self N2 (Efficacy or concept)) ) OR AB ( (Self N2 (Efficacy or concept)) ) | 34,305 |
| S21 | TI ( (mortal* or ((death or fatality) N2 (rate* or frequenc*))) ) OR AB ( (mortal* or ((death or fatality) N2 (rate* or frequenc*))) ) | 257,162 |
| S20 | (MM "Readmission") or (MM "Mortality") or (MM "Quality of Life") or (MM "Costs and Cost Analysis") or (MM "Self-Efficacy") or (MM "Outcomes (Health Care)") or (MM "Quality of Health Care") or (MM "Survey Research") or (MM "Hospitalization") | 194,824 |
| S19 | S15 or S16 or S17 or S18 | 1,921,799 |
| S18 | TI ( (Health N3 (Knowledge or attitude* or practice*)) ) OR AB ( (Health N3 (Knowledge or attitude* or practice*)) ) | 47,148 |
| S17 | TI ( (implement* or evaluat* or coordinat* or strateg* or barrier* or block* or obstacle* or hinder* or constrain* or facilitat* or incentiv* or challenge* or enabler*) ) OR AB ( (implement* or evaluat* or coordinat* or strateg* or barrier* or block* or obstacle* or hinder* or constrain* or facilitat* or incentiv* or challenge* or enabler*) ) | 1,827,748 |
| S16 | TI ( ((system* or program* or process*) N3 (analy* or integrat* or evaluat* or implement*)) ) OR AB ( ((system* or program* or process*) N3 (analy* or integrat* or evaluat* or implement*)) ) | 195,38 |
| S15 | (MM "Implementation Science") or (MM "Systems Analysis") or (MM "Program Evaluation") or (MM "Evaluation Research") | 22,79 |
| S14 | S8 or S9 or S10 or S11 or S12 or S13 | 203,599 |
| S13 | TI ( (case N3 (manag* or plan* or coordinat* or program*)) ) OR AB ( (case N3 (manag* or plan* or coordinat* or program*)) ) | 26,262 |
| S12 | TI ( (care N3 (coordinat* or plan* or manag* or goal* or contin* or process*)) ) OR AB ( (care N3 (coordinat* or plan* or manag* or goal* or contin* or process*)) ) | 111,143 |
| S11 | TI ( ((handover or handoff* or "hand over" or "hand off*" or signout* or "sign out*" or signover or "sign over") N3 (patient* or plan* or coordinat* or manag* or program*)) ) OR AB ( ((handover or handoff* or "hand over" or "hand off*" or signout* or "sign out*" or signover or "sign over") N3 (patient* or plan* or coordinat* or manag* or program*)) ) | 1,161 |
| S10 | TI ( ((plan* or admission or discharge or postdischarge) N3 (coordinat* or contin* or manag* or process*)) ) OR AB ( ((plan* or admission or discharge or postdischarge) N3 (coordinat* or contin* or manag* or process*)) ) | 23,354 |
| S9 | TI ( ((transition* or discharge or postdischarge or handover or "follow up" or stepdown or "step down" or multidisciplinary) N3 (care or treat* or therapy or coordinat*)) ) OR AB ( ((transition* or discharge or postdischarge or handover or "follow up" or stepdown or "step down" or multidisciplinary) N3 (care or treat* or therapy or coordinat*)) ) | 51,066 |
| S8 | (MM "Transitional Care") or (MM "Continuity of Patient Care") OR ( (MM "Patient Care Plans") or (MM "Case Management") | 25,466 |
| S7 | S1 or S2 or S3 or S4 or S5 or S6 | 246,311 |
| S6 | TI ( (polymorbidit* or Plurimorbidit* or ((multiple or polypathic) N2 (condition* or disease* or disorder* or illness* or patholog* or "health problem*"))) ) OR AB ( (polymorbidit* or Plurimorbidit* or ((multiple or polypathic) N2 (condition* or disease* or disorder* or illness* or patholog* or "health problem*"))) ) | 12,148 |
| S5 | TI ( (comorbidit* or "co morbidit*" or multimorbidit* or (("intercurrent" or concurrent) N2 (illness* or disorder* or disease* or condition* or patholog* or "health problem*"))) ) OR AB ( (comorbidit* or "co morbidit*" or multimorbidit* or (("intercurrent" or concurrent) N2 (illness* or disorder* or disease* or condition* or patholog* or "health problem*"))) ) | 77,149 |
| S4 | TI ( (multiple N3 (chronic N3 (condition* or disease* or disorder* or ill* or patholog* or "health problem*"))) ) OR AB ( (multiple N3 (chronic N3 (condition* or disease* or disorder* or ill* or patholog* or "health problem*"))) ) | 2,622 |
| S3 | TI ( ((complex or "high risk") N3 (patient* or medically or adult*)) ) OR AB ( ((complex or "high risk") N3 (patient* or medically or adult*)) ) | 41,742 |
| S2 | TI ( ((complex or "long term" or chronic or multidisciplinary) N3 (care or treat* or therapy)) ) OR AB ( ((complex or "long term" or chronic or multidisciplinary) N3 (care or treat* or therapy)) ) | 108,715 |
| S1 | (MM "Long Term Care") OR (MM "Comorbidity") | 34,081 |

Database - CENTRAL July 16, 2024

| ID | Search | Hits |
| --- | --- | --- |
| #1 | MeSH descriptor: [Long-Term Care] explode all trees | 1534 |
| #2 | MeSH descriptor: [Comorbidity] explode all trees | 5289 |
| #3 | MeSH descriptor: [Multimorbidity] explode all trees | 156 |
| #4 | ((complex or "long term" or chronic or multidisciplinary) near/3 (care or treat* or therapy)):ti,ab,kw | 70444 |
| #5 | ((complex or "high risk") near/3 (patient* or medically or adult*)):ti,ab,kw | 24001 |
| #6 | (multiple near/3 (chronic near/3 (condition* or disease* or disorder* or ill* or patholog* or "health problem*"))):ti,ab,kw | 807 |
| #7 | (comorbidit* or "co morbidit*" or multimorbidit* or (("intercurrent" or concurrent) near/2 (illness* or disorder* or disease* or condition* or patholog* or "health problem*"))):ti,ab,kw | 28693 |
| #8 | (polymorbidit* or Plurimorbidit* or ((multiple or polypathic) near/2 (condition* or disease* or disorder* or illness* or patholog* or "health problem*"))):ti,ab,kw | 4483 |
| #9 | {or #1-#8} | 122357 |
| #10 | MeSH descriptor: [Transitional Care] explode all trees | 146 |
| #11 | MeSH descriptor: [Continuity of Patient Care] explode all trees | 39633 |
| #12 | MeSH descriptor: [Patient Care Planning] explode all trees | 2514 |
| #13 | MeSH descriptor: [Case Management] explode all trees | 931 |
| #14 | ((transition* or discharge or postdischarge or handover or "follow up" or stepdown or "step down" or multidisciplinary) near/3 (care or treat* or therapy or coordinat*)):ti,ab,kw | 8846 |
| #15 | ((plan* or admission or discharge or postdischarge) near/3 (coordinat* or contin* or manag* or process*)):ti,ab,kw | 5090 |
| #16 | ((handover or handoff* or "hand over" or "hand off*" or signout* or "sign out*" or signover or "sign over") near/3 (patient* or plan* or coordinat* or manag* or program*)):ti,ab,kw | 143 |
| #17 | (care near/3 (coordinat* or plan* or manag* or goal* or contin* or process*)):ti,ab,kw | 24535 |
| #18 | (case near/3 (manag* or plan* or coordinat* or program*)):ti,ab,kw | 4006 |
| #19 | {or #10-#18} | 75542 |
| #20 | MeSH descriptor: [Implementation Science] explode all trees | 98 |
| #21 | MeSH descriptor: [Systems Analysis] explode all trees | 2749 |
| #22 | MeSH descriptor: [Program Evaluation] explode all trees | 7884 |
| #23 | MeSH descriptor: [Evaluation Study] explode all trees | 1 |
| #24 | MeSH descriptor: [Health Knowledge, Attitudes, Practice] explode all trees | 8280 |
| #25 | ((system* or program* or process*) near/3 (analy* or integrat* or evaluat* or implement*)):ti,ab,kw | 41263 |
| #26 | (implement* or evaluat* or coordinat* or strateg* or barrier* or block* or obstacle* or hinder* or constrain* or facilitat* or incentiv* or challenge* or enabler*):ti,ab,kw | 870361 |
| #27 | (Health near/3 (Knowledge or attitude* or practice*)):ti,ab,kw | 20936 |
| #28 |  | 885038 |
| #29 | MeSH descriptor: [Patient Readmission] explode all trees | 1628 |
| #30 | MeSH descriptor: [Mortality] explode all trees | 18908 |
| #31 | MeSH descriptor: [Quality of Life] explode all trees | 44306 |
| #32 | MeSH descriptor: [Costs and Cost Analysis] explode all trees | 16567 |
| #33 | MeSH descriptor: [Self Efficacy] explode all trees | 4374 |
| #34 | MeSH descriptor: [Outcome Assessment, Health Care] explode all trees | 214716 |
| #35 | MeSH descriptor: [Quality of Health Care] explode all trees | 658458 |
| #36 | MeSH descriptor: [Quality Indicators, Health Care] explode all trees | 1016 |
| #37 | MeSH descriptor: [Surveys and Questionnaires] explode all trees | 78081 |
| #38 | MeSH descriptor: [Hospitalization] explode all trees | 20485 |
| #39 | (rehospitalization or hospitali* or ((patient* or hospital* or "30 day" or "thirty day" or unplanned) near/3 (readmission* or re admission))):ti,ab,kw | 94577 |
| #40 | (mortal* or ((death or fatality) near/2 (rate* or frequenc*))):ti,ab,kw | 124974 |
| #41 | (Self near/2 (efficacy or concept)):ti,ab,kw | 25611 |
| #42 | (Outcome near/3 (assess* or patient* or clinical* or "health Care")):ti,ab,kw | 186295 |
| #43 | (Survey* or questionnaire* or cost*):ti,ab,kw | 306875 |
| #44 | (QOL or hrql or hrqol or (Quality near/2 (Healthcare or care or life))):ti,ab,kw | 185646 |
| #45 | (Husereau et al., -#44) | 1077293 |
| #46 | #9 and #19 and #28 and #45 | 5657 |
| #47 | MeSH descriptor: [Adult] explode all trees | 618401 |
| #48 | MeSH descriptor: [Aged] explode all trees | 276556 |
| #49 | MeSH descriptor: [Geriatrics] explode all trees | 300 |
| #50 | (adult* or senior* or aged or elder* or geriatri* or ((old* or mature*) near/2 (people* or subject* or patient* or age* or men or male* or wom?n or female* or population* or cohort* or person*))):ti,ab,kw | 1135402 |
| #51 | {or #47-#50} | 1135402 |
| #52 | #46 and #51 | 4317 |
| #53 | ((Palliative or "end of life") near/3 care):ti,kw | 4037 |
| #54 | (experiment* model* or animal* or monkey* or sheep or ?ovine or lamb* or goat* or pig* or swine or porcine or pup* or dog* or canine or bitch* or beagle* or feline or rodent* or rabbit* or rat or rats or mouse or murine or mice):ti,kw | 40722 |
| #55 |  | 44729 |
| #56 | #52 not #55 | 4187 |
|  |  | 4134 trials |

## Excluded articles and reasons for exclusion

### Wrong intervention (e.g., non-multidisciplinary intervention)

1. Aboumatar H, Naqibuddin M, Chung S, Chaudhry H, Kim SW, Saunders J, Bone L, Gurses AP, Knowlton A, Pronovost P, Putcha N. Effect of a hospital-initiated program combining transitional care and long-term self-management support on outcomes of patients hospitalized with chronic obstructive pulmonary disease: a randomized clinical trial. Jama. 2019 Oct 8;322(14):1371-80.
2. Askim T, Mørkved S, Engen A, Roos K, Aas T, Indredavik B. Effects of a community-based intensive motor training program combined with early supported discharge after treatment in a comprehensive stroke unit: a randomized, controlled trial. Stroke. 2010 Aug 1;41(8):1697-703.
3. Barnason S, Miller JN, Schuelke S, Miller JJ, Kupzyk K. Self‐management intervention for patients following hospitalization for acute exacerbation of chronic obstructive pulmonary disease (AECOPD): A pilot randomized controlled trial. Nursing & Health Sciences. 2024 Jun;26(2):e13114.
4. Baxter R, Murray J, Cockayne S, Baird K, Mandefield L, Mills T, Lawton R, Hewitt C, Richardson G, Sheard L, O’Hara JK. Improving the safety and experience of transitions from hospital to home: a cluster randomised controlled feasibility trial of the'Your Care Needs You'intervention versus usual care. Pilot and Feasibility Studies. 2022 Oct 1;8(1):222.
5. Bell KR, Temkin NR, Esselman PC, Doctor JN, Bombardier CH, Fraser RT, Hoffman JM, Powell JM, Dikmen S. The effect of a scheduled telephone intervention on outcome after moderate to severe traumatic brain injury: a randomized trial. Archives of physical medicine and rehabilitation. 2005 May 1;86(5):851-6.
6. Beney J, Devine EB, Chow V, Ignoffo RJ, Mitsunaga L, Shahkarami M, McMillan A, Bero LA. Effect of telephone follow‐up on the physical well‐being dimension of quality of life in patients with cancer. Pharmacotherapy: The Journal of Human Pharmacology and Drug Therapy. 2002 Oct;22(10):1301-11.
7. Boden-Albala B, Goldmann E, Parikh NS, Carman H, Roberts ET, Lord AS, Torrico V, Appleton N, Birkemeier J, Parides M, Quarles L. Efficacy of a discharge educational strategy vs standard discharge care on reduction of vascular risk in patients with stroke and transient ischemic attack: the DESERVE randomized clinical trial. JAMA neurology. 2019 Jan 1;76(1):20-7.
8. Costi S, Pellegrini M, Braglia L, Cavuto S, Fugazzaro S. Occupational therapy improves social participation of complex patients discharged from hospital: results of a powered randomized controlled trial. Disability and Rehabilitation. 2024 May 21;46(11):2223-33.
9. Davis KM, Dawson D, Kelly S, Red S, Penek S, Lynch J, Collins S, Lynch B, Porrazzo M, Bass M, Taylor KL. Monitoring of health-related quality of life and symptoms in prostate cancer survivors: a randomized trial. J Support Oncol. 2013 Dec 1;11(4):174-82.
10. Dawes HA, Docherty T, Traynor I, Gilmore DH, Jardine AG, Knill-Jones R. Specialist nurse supported discharge in gynaecology: A randomised comparison and economic evaluation. European Journal of Obstetrics & Gynecology and Reproductive Biology. 2007 Feb 1;130(2):262-70.
11. de Souza EN, Rohde LE, Ruschel KB, Mussi CM, Beck‐da‐Silva L, Biolo A, Clausell N, Rabelo‐Silva ER. A nurse‐based strategy reduces heart failure morbidity in patients admitted for acute decompensated heart failure in Brazil: the HELEN‐II clinical trial. European Journal of Heart Failure. 2014 Sep;16(9):1002-8.
12. Avci YD, Gözüm S. Effects of Transitional Care Model–Based Interventions for Stroke Patients and Caregivers on Caregivers' Competence and Patient Outcomes: Randomized Controlled Trial. CIN: Computers, Informatics, Nursing. 2023 Oct 1;41(10):805-14.
13. Donzé J, John G, Genné D, Mancinetti M, Gouveia A, Méan M, Bütikofer L, Aujesky D, Schnipper J. Effects of a multimodal transitional care intervention in patients at high risk of readmission: the TARGET-READ randomized clinical trial. JAMA Internal Medicine. 2023 Jul 1;183(7):658-68.
14. Edmans J, Bradshaw L, Franklin M, Gladman J, Conroy S. Specialist geriatric medical assessment for patients discharged from hospital acute assessment units: randomised controlled trial. Bmj. 2013 Oct 8;347.
15. Fabrés Martín C, Ventura Parellada C, Herrero Antón de Vez H, Ordoñez Urgiles CE, Alonso-Rodriguez Piedra J, Mora Guix JM. Telemedicine approach for patient follow-up after total knee and reverse total shoulder arthroplasty: a pilot study. International Journal of Computer Assisted Radiology and Surgery. 2023 Mar;18(3):595-602.
16. Faithfull S, Corner J, Meyer L, Huddart R, Dearnaley D. Evaluation of nurse-led follow up for patients undergoing pelvic radiotherapy. British journal of cancer. 2001 Dec;85(12):1853-64.
17. Fitzgerald JF, Smith DM, Martin DK, Freedman JA, Katz BP. A case manager intervention to reduce readmissions. Archives of internal medicine. 1994 Aug 8;154(15):1721-9.
18. Guo J, Zhao X, Xu C. Effects of a continuous nursing care model on elderly patients with total hip arthroplasty: a randomized controlled trial. Aging Clinical and Experimental Research. 2022 Jul;34(7):1603-11.
19. Hernandez-Quiles C, Bernabeu-Wittel M, Barón-Franco B, Palacios AA, Garcia-Serrano MR, Lopez-Jimeno W, Antonio Perez-de-Leon-Serrano J, Gómez-Barranco JM, Ruiz-Cantero A, Quero-Haro M, Cubiles-Montero E. A randomized clinical trial of home telemonitoring in patients with advanced heart and lung diseases. Journal of Telemedicine and Telecare. 2024 Feb;30(2):356-64.
20. Heydarikhayat N, Ashktorab T, Rohani C, Zayeri F. Effect of post-hospital discharge follow-up on health status in patients with burn injuries: a randomized clinical trial. International journal of community based nursing and midwifery. 2018 Oct;6(4):293.
21. Higgins HC, Hayes RL, McKenna KT. Rehabilitation outcomes following percutaneous coronary interventions (PCI). Patient education and counseling. 2001 Jun 1;43(3):219-30.
22. Hoffmann T, Ownsworth T, Eames S, Shum D. Evaluation of brief interventions for managing depression and anxiety symptoms during early discharge period after stroke: a pilot randomized controlled trial. Topics in stroke rehabilitation. 2015 Apr 1;22(2):116-26.
23. Iraurgui BA, Muñiz J, Rodríguez-Fernández JA, Vidán-Martínez L, Silva-César M, Lamelo-Alfonsín F, Díaz-Díaz JL, Ramos-Polledo V, Castro-Beiras A. Randomized controlled clinical trial of a home care unit intervention to reduce readmission and death rates in patients discharged from hospital following admission for heart failure. Revista española de cardiología. 2007 Sep 1;60(9):914.
24. Kampan P. Effects of counseling and implementation of clinical pathway on diabetic patients hospitalized with hypoglycemia. Journal-Medical Association of Thailand. 2006 May 1;89(5):619.
25. Kimchi A, Aronow HU, Ni YM, Ong MK, Mirocha J, Black JT, Auerbach AD, Ganiats TG, Greenfield S, Romano PS, Kedan I. Postdischarge Noninvasive Telemonitoring and Nurse Telephone Coaching Improve Outcomes in Heart Failure Patients With High Burden of Comorbidity. Journal of cardiac failure. 2023 May 1;29(5):774-83.
26. Kvale EA, Huang CH, Meneses KM, Demark‐Wahnefried W, Bae S, Azuero CB, Rocque GB, Bevis KS, Ritchie CS. Patient‐centered support in the survivorship care transition: outcomes from the patient‐owned survivorship care plan intervention. Cancer. 2016 Oct 15;122(20):3232-42.
27. Kowalkowski M, Chou SH, McWilliams A, Lashley C, Murphy S, Rossman W, Papali A, Heffner A, Russo M, Burke L, Gibbs M. Structured, proactive care coordination versus usual care for Improving Morbidity during Post-Acute Care Transitions for Sepsis (IMPACTS): a pragmatic, randomized controlled trial. Trials. 2019 Dec;20:1-3.
28. Lindley RI, Anderson CS, Billot L, Forster A, Hackett ML, Harvey LA, Jan S, Li Q, Liu H, Langhorne P, Maulik PK. Family-led rehabilitation after stroke in India (ATTEND): a randomised controlled trial. The Lancet. 2017 Aug 5;390(10094):588-99.
29. Lemke M, Kappel R, McCarter R, D’Angelo L, Tuchman LK. Perceptions of health care transition care coordination in patients with chronic illness. Pediatrics. 2018 May 1;141(5).
30. Mínguez Clemente P, Pascual-Carrasco M, Mata Hernández C, Malo de Molina R, Arvelo LA, Cadavid B, López F, Sánchez-Madariaga R, Sam A, Trisan Alonso A, Valle Falcones M. Follow-up with telemedicine in early discharge for COPD exacerbations: randomized clinical trial (TELEMEDCOPD-trial). COPD: Journal of Chronic Obstructive Pulmonary Disease. 2020 Nov 29;18(1):62-9.
31. Mizukawa M, Moriyama M, Yamamoto H, Rahman MM, Naka M, Kitagawa T, Kobayashi S, Oda N, Yasunobu Y, Tomiyama M, Morishima N. Nurse-led collaborative management using telemonitoring improves quality of life and prevention of rehospitalization in patients with heart failure a pilot study. International heart journal. 2019 Nov 30;60(6):1293-302.
32. Moffet H, Tousignant M, Nadeau S, Mérette C, Boissy P, Corriveau H, Marquis F, Cabana F, Ranger P, Belzile EL, Dimentberg R. In-home telerehabilitation compared with face-to-face rehabilitation after total knee arthroplasty: a noninferiority randomized controlled trial. JBJS. 2015 Jul 15;97(14):1129-41.
33. Molazem Z, Rezaei S, Mohebbi Z, Ostovan MA, Keshavarzi S. Effect of continuous care model on lifestyle of patients with myocardial infarction. ARYA atherosclerosis. 2013 May;9(3):186.
34. Muschol J, Heinrich M, Heiss C, Hernandez AM, Knapp G, Repp H, Schneider H, Thormann U, Uhlar J, Unzeitig K, Gissel C. Economic and environmental impact of digital health app video consultations in follow-up care for patients in orthopedic and trauma surgery in Germany: randomized controlled trial. Journal of Medical Internet Research. 2022 Nov 24;24(11):e42839.
35. Muschol J, Heinrich M, Heiss C, Knapp G, Repp H, Schneider H, Thormann U, Uhlar J, Unzeitig K, Gissel C. Assessing telemedicine efficiency in follow-up care with video consultations for patients in orthopedic and trauma surgery in Germany: randomized controlled trial. Journal of Medical Internet Research. 2022 Jul 27;24(7):e36996.
36. Nathan JA, Pearce L, Field C, Dotesio-Eyres N, Sharples LD, Cafferty F, Laroche CM. A randomized controlled trial of follow-up of patients discharged from the hospital following acute asthma: best performed by specialist nurse or doctor?. Chest. 2006 Jul 1;130(1):51-7.
37. Naylor MD, Brooten D, Campbell R, Jacobsen BS, Mezey MD, Pauly MV, Schwartz JS. Comprehensive discharge planning and home follow-up of hospitalized elders: a randomized clinical trial. Jama. 1999 Feb 17;281(7):613-20.
38. Naylor MD, McCauley KM. The effects of a discharge planning and home follow-up intervention on elders hospitalized with common medical and surgical cardiac conditions. Journal of Cardiovascular Nursing. 1999 Oct 1;14(1):44-54.
39. Neelemaat F, Bosmans JE, Thijs A, Seidell JC. Post-discharge nutritional support in malnourished elderly individuals improves functional limitations. Journal of the American Medical Directors Association. 2011 May 1;12(4):295-301.
40. Ojeda A, Calvo A, Cuñat T, Mellado-Artigas R, Costas-Carrera A, Sánchez-Rodriguez MM, Comino-Trinidad O, Aliaga J, Arias M, Martínez-Pallí G, Dürsteler C. Effectiveness of a specific follow up program for the management of the mental components of post-intensive care syndrome and chronic pain after COVID-19: results from the PAIN-COVID randomized clinical trial. Revista Española de Anestesiología y Reanimación (English Edition). 2024 May 1;71(5):349-59.
41. Pagano E, Pellegrino L, Robella M, Castiglione A, Brunetti F, Giacometti L, Rolfo M, Rizzo A, Palmisano S, Meineri M, Bachini I. Implementation of an enhanced recovery after surgery protocol for colorectal cancer in a regional hospital network supported by audit and feedback: a stepped wedge, cluster randomised trial. BMJ Quality & Safety. 2024 Jun 1;33(6):363-74.
42. Patel PK, Shukla AK, Sachan V, Sharma P, Singh S, Saxena S, Makkad RS. Evaluation of the Effectiveness of Telemedicine in Postoperative Follow-Up Care After Dental Implant Surgery. A Pilot Study. Journal of Pharmacy and Bioallied Sciences. 2024 Feb 1;16(Suppl 1):S463-5.
43. Pietrantonio F, Vinci A, Rosiello F, Alessi E, Pascucci M, Rainone M, Delli Castelli M, Ciamei A, Montagnese F, D’Amico R, Valerio A. Green Line Hospital-Territory Study: A Single-Blind Randomized Clinical Trial for Evaluation of Technological Challenges of Continuous Wireless Monitoring in Internal Medicine, Preliminary Results. International journal of environmental research and public health. 2021 Sep 30;18(19):10328.
44. Polovneff A, Shah N, Janardan A, Smith E, Pasillas I, Mortensen N, Holt JM, Somai M, Sparapani R, Crotty B. Scaling Care Coordination Through Digital Engagement: Stepped-Wedge Trial Assessing Readmissions. American Journal of Managed Care. 2024 Feb 1;30(2).
45. Qian C, Zhong D, Shen Y, Du Q. Evaluation of clinical efficacy of transitional care mode for patients with strokes. Int J Clin Exp Med. 2019 Jan 1;12(1):981-8.
46. Ramsay P, Huby G, Merriweather J, Salisbury L, Rattray J, Griffith D, Walsh T. Patient and carer experience of hospital-based rehabilitation from intensive care to hospital discharge: mixed methods process evaluation of the RECOVER randomised clinical trial. BMJ open. 2016 Jul 1;6(8):e012041.
47. Renehan E, Meyer C, Elliott RA, Batchelor F, Said C, Haines T, Goeman D. Posthospital Falls Prevention Intervention: A Mixed-Methods Study. Journal of Aging & Physical Activity. 2019 Apr 1;27(2).
48. Ritchie CS, Houston TK, Richman JS, Sobko HJ, Berner ES, Taylor BB, Salanitro AH, Locher JL. The E-Coach technology-assisted care transition system: a pragmatic randomized trial. Translational behavioral medicine. 2016 Sep 1;6(3):428-37.
49. Sewell J, McDaniel CC, Harris SM, Chou C. Implementation of a pharmacist-led transitions of care program in an indigent care clinic: a randomized controlled trial. Journal of the American Pharmacists Association. 2021 May 1;61(3):276-83.
50. Seys D, Bruyneel L, Sermeus W, Lodewijckx C, Decramer M, Deneckere S, Panella M, Vanhaecht K. Teamwork and adherence to recommendations explain the effect of a care pathway on reduced 30-day readmission for patients with a COPD exacerbation. COPD: Journal of Chronic Obstructive Pulmonary Disease. 2018 Mar 4;15(2):157-64.
51. Sezgin D, Mert H, Özpelit E, Akdeniz B. The effect on patient outcomes of a nursing care and follow-up program for patients with heart failure: A randomized controlled trial. International journal of nursing studies. 2017 May 1;70:17-26.
52. Sunnerhagen KS, Danielsson A, Rafsten L, Björkdahl A, Axelsson ÅB, Nordin Å, Petersson CA, Lundgren-Nilsson Å, Fröjd K. Gothenburg very early supported discharge study (GOTVED) NCT01622205: a block randomized trial with superiority design of very early supported discharge for patients with stroke. BMC neurology. 2013 Dec;13:1-8.
53. Thompson DR, Roebuck A, Stewart S. Effects of a nurse‐led, clinic and home‐based intervention on recurrent hospital use in chronic heart failure. European Journal of Heart Failure. 2005 Mar;7(3):377-84.
54. Utriyaprasit K, Moore SM, Chaiseri P. Recovery after coronary artery bypass surgery: effect of an audiotape information programme. Journal of advanced nursing. 2010 Aug;66(8):1747-59.
55. Valeiro B, Rodríguez E, Pérez P, Gómez A, Mayer AI, Pasarín A, Ibañez J, Ferrer J, Ramon MA. Promotion of physical activity after hospitalization for COPD exacerbation: A randomized control trial. Respirology. 2023 Apr;28(4):357-65.
56. Vanhaecht K, Lodewijckx C, Sermeus W, Decramer M, Deneckere S, Leigheb F, Boto P, Kul S, Seys D, Panella M. Impact of a care pathway for COPD on adherence to guidelines and hospital readmission: a cluster randomized trial. International journal of chronic obstructive pulmonary disease. 2016 Nov 23:2897-908.
57. You J, Wang S, Li J, Luo Y. Usefulness of a nurse-led program of care for management of patients with chronic heart failure. Medical Science Monitor: International Medical Journal of Experimental and Clinical Research. 2020;26:e920469-1.
58. Young JM, Butow PN, Walsh J, Durcinoska I, Dobbins TA, Rodwell L, Harrison JD, White K, Gilmore A, Hodge B, Hicks H. Multicenter randomized trial of centralized nurse-led telephone-based care coordination to improve outcomes after surgical resection for colorectal cancer: the CONNECT intervention. Journal of Clinical Oncology. 2013 Oct 1;31(28):3585-91.
59. Zhang Y, Sun H, Zhang X, Liang J, Zheng X. Group Visits Improve Health in Patients With COPD in Post-Discharge Transition Period. Alternative Therapies in Health & Medicine. 2022 Feb 1;28(2).
60. Zimmerman L, Wilson FA, Schmaderer MS, Struwe L, Pozehl B, Paulman A, Bratzke LC, Moore K, Raetz L, George B. Cost-effectiveness of a care transition intervention among multimorbid patients. Western journal of nursing research. 2017 May;39(5):622-42.

### Wrong outcome (e.g., costs)

1. Ackermann RT, Liss DT, French DD, Cooper AJ, Aikman C, Schaeffer C. Randomized Trial Evaluating Health System Expenditures with Transitional Care Services for Adults with No Usual Source of Care at Discharge. Journal of general internal medicine. 2022 Nov;37(15):3832-8.
2. Freburger JK, Pastva AM, Coleman SW, Peter KM, Kucharska-Newton AM, Johnson AM, Psioda MA, Duncan PW, Bushnell CD, Rosamond WD, Jones SB. Skilled nursing and inpatient rehabilitation facility use by medicare fee-for-service beneficiaries discharged home after a stroke: Findings From the COMPASS trial. Archives of Physical Medicine and Rehabilitation. 2022 May 1;103(5):882-90.
3. Galbraith AA, Meyers DJ, Ross‐Degnan D, Burns ME, Vialle‐Valentin CE, Larochelle MR, Touw S, Zhang F, Rosenthal M, Balaban RB. Long‐term impact of a postdischarge community health worker intervention on health care costs in a safety‐net system. Health services research. 2017 Dec;52(6):2061-78.
4. Graves N, Courtney M, Edwards H, Chang A, Parker A, Finlayson K. Cost-effectiveness of an intervention to reduce emergency re-admissions to hospital among older patients. PLOS one. 2009 Oct 14;4(10):e7455.
5. Latour CH, Bosmans JE, van Tulder MW, de Vos R, Huyse FJ, de Jonge P, van Gemert LA, Stalman WA. Cost-effectiveness of a nurse-led case management intervention in general medical outpatients compared with usual care: an economic evaluation alongside a randomized controlled trial. Journal of psychosomatic research. 2007 Mar 1;62(3):363-70.
6. Rubin CD, Sizemore MT, Loftis PA, Adams‐Huet B, Anderson RJ. The effect of geriatric evaluation and management on Medicare reimbursement in a large public hospital: a randomized clinical trial. Journal of the American Geriatrics Society. 1992 Oct;40(10):989-95.
7. Verweij L, Petri AC, MacNeil-Vroomen JL, Jepma P, Latour CH, Peters RJ, Scholte op Reimer WJ, Buurman BM, Bosmans JE. The Cardiac Care Bridge transitional care program for the management of older high-risk cardiac patients: An economic evaluation alongside a randomized controlled trial. Plos one. 2022 Jan 27;17(1):e0263130.
8. Yan C, Round J, Akpinar I, Atwood CE, Deuchar L, Bhutani M, Leigh R, Stickland MK. Cost analysis of a transition care bundle compared with usual care for COPD patients being discharged from hospital: evaluation of a randomized controlled trial. PharmacoEconomics-Open. 2023 May;7(3):493-505.

### Wrong population (e.g., patients visiting the emergency department and discharged home without hospital stay, patients discharged to a lonmg-term care facility)

1. Andersen AL, Houlind MB, Nielsen RL, Jørgensen LM, Bengaard AK, Bornæs O, Juul-Larsen HG, Hansen NM, Brøchner LD, Hansen RG, Skovlund CA. Effectiveness of a multidisciplinary and transitional nutritional intervention compared with standard care on health-related quality of life among acutely admitted medical patients aged≥ 65 years with malnutrition or risk of malnutrition: A randomized controlled trial. Clinical Nutrition ESPEN. 2024 Jun 1;61:52-62.
2. Blakeman T, Blickem C, Kennedy A, Reeves D, Bower P, Gaffney H, Gardner C, Lee V, Jariwala P, Dawson S, Mossabir R. Effect of information and telephone-guided access to community support for people with chronic kidney disease: randomised controlled trial. PloS one. 2014 Oct 16;9(10):e109135.
3. Boockvar KS, Koufacos NS, May J, Schwartzkopf AL, Guerrero VM, Judon KM, Schubert CC, Franzosa E, Dixon BE. Effect of health information exchange plus a care transitions intervention on post-hospital outcomes among VA primary care patients: a randomized clinical trial. Journal of General Internal Medicine. 2022 Dec;37(16):4054-61.
4. Brown AF, Behforouz H, Shah A, Lewis J, Ettner S, Porter C, Majeno A, Huang DY, Vassar SD, Carson S, Kim KJ. THE CARE CONNECTIONS PROGRAM: A RANDOMIZED TRIAL OF COMMUNITY HEALTH WORKERS TO IMPROVE CARE FOR MEDICALLY AND SOCIALLY COMPLEX PATIENTS. InJOURNAL OF GENERAL INTERNAL MEDICINE 2020 Jul 1 (Vol. 35, No. SUPPL 1, pp. S288-S288). ONE NEW YORK PLAZA, SUITE 4600, NEW YORK, NY, UNITED STATES: SPRINGER.
5. Buurman BM, Parlevliet JL, Allore HG, Blok W, van Deelen BA, van Charante EP, de Haan RJ, de Rooij SE. Comprehensive geriatric assessment and transitional care in acutely hospitalized patients: the transitional care bridge randomized clinical trial. JAMA internal medicine. 2016 Mar 1;176(3):302-9.
6. Ducharme A, Doyon O, White M, Rouleau JL, Brophy JM. Impact of care at a multidisciplinary congestive heart failure clinic: a randomized trial. Cmaj. 2005 Jul 5;173(1):40-5.
7. Eklund K, Wilhelmson K, Gustafsson H, Landahl S, Dahlin-Ivanoff S. One-year outcome of frailty indicators and activities of daily living following the randomised controlled trial;“Continuum of care for frail older people”. BMC geriatrics. 2013 Dec;13:1-0.
8. Fjærtoft H, Indredavik B, Lydersen S. Stroke unit care combined with early supported discharge: long-term follow-up of a randomized controlled trial. Stroke. 2003 Nov 1;34(11):2687-91.
9. Fjærtoft H, Rohweder G, Indredavik B. Stroke unit care combined with early supported discharge improves 5-year outcome. Stroke. 2011.
10. Forster A, Young J, Chapman K, Nixon J, Patel A, Holloway I, Mellish K, Anwar S, Breen R, Knapp M, Murray J. Cluster randomized controlled trial: clinical and cost-effectiveness of a system of longer-term stroke care. Stroke. 2015 Aug;46(8):2212-9.
11. Kazawa K, Kubo T, Ohge H, Ishii S. Efficacy of care manager-led support for family caregivers of people with dementia during the COVID-19 pandemic: a randomized controlled study. BMC geriatrics. 2022 Aug 15;22(1):671.
12. Nguyen V, Ducharme A, White M, Racine N, O'Meara E, Zhang B, Rouleau JL, Brophy J. Lack of long-term benefits of a 6-month heart failure disease management program. Journal of Cardiac Failure. 2007 May 1;13(4):287-93.
13. Sharshar T, Grimaldi-Bensouda L, Siami S, Cariou A, Salah AB, Kalfon P, Sonneville R, Meunier-Beillard N, Quenot JP, Megarbane B, Gaudry S. A randomized clinical trial to evaluate the effect of post-intensive care multidisciplinary consultations on mortality and the quality of life at 1 year. Intensive Care Medicine. 2024 Apr 8:1-3.
14. Shaw M. Integrated care pathway based rehabilitation for acute stroke did not reduce length of hospital stay. Evidence-Based Nursing. 2001 Apr;4(2):53-.
15. Shuen JA, Wilson MP, Kreshak A, Mullinax S, Brennan J, Castillo EM, Hinkle C, Vilke GM. Telephoned, Texted, or Typed Out: a randomized trial of physician–patient communication after emergency department discharge. The Journal of emergency medicine. 2018 Oct 1;55(4):573-81.
16. Soto Perez De Celis E, Chavarri Guerra Y, Ramos-Lopez WA, Covarrubias-Gómez A, Navarro-Lara A, Quiroz P, Sanchez S, Alcocer N, Alcalde Castro M, Aguilar Velazco JC, Bukowski A. Randomized controlled trial (RCT) of a patient navigation (PN) intervention to increase early access to supportive care (SC) for patients with metastatic cancer in a resource-limited setting.
17. Sulch D, Evans A, Melbourn A, Kalra L. Does an integrated care pathway improve processes of care in stroke rehabilitation? A randomized controlled trial. Age and Ageing. 2002 May 1;31(3):175-9.
18. Sulch D, Perez I, Melbourn A, Kalra L. Randomized controlled trial of integrated (managed) care pathway for stroke rehabilitation. Stroke. 2000 Aug;31(8):1929-34.
19. Yu CM, Lau CP, Chau J, McGhee S, Kong SL, Cheung BM, Li LS. A short course of cardiac rehabilitation program is highly cost effective in improving long-term quality of life in patients with recent myocardial infarction or percutaneous coronary intervention. Archives of physical medicine and rehabilitation. 2004 Dec 1;85(12):1915-22.
20. Zhang YY, Zhang QX, Li JT, Wang Y, Zhuang ZH, Zhuang JY. Clinical Pathway for Enhanced Recovery in the Management of Non-Variceal Upper Gastrointestinal Bleeding: A Randomized Controlled Trial. Risk Management and Healthcare Policy. 2023 Dec 31:2579-91.

### Wrong study design (e.g., protocol)

1. Diplock G, Ward J, Stewart S, Scuffham P, Stewart P, Reeve C, Davidson L, Maguire G. The Alice Springs Hospital Readmission Prevention Project (ASHRAPP): a randomised control trial. BMC health services research. 2017 Dec;17:1-1.
2. Eichner FA, Schwarzbach CJ, Keller M, Haeusler KG, Hamann GF, Sander D, Audebert HJ, Gröschel K, Geis D, von Bandemer S, Rücker V. Trial design and pilot phase results of a cluster-randomised intervention trial to improve stroke care after hospital discharge–The structured ambulatory post-stroke care program (SANO). European Stroke Journal. 2021 Jun;6(2):213-21.
3. Griffiths P. Advanced practice nurse directed transitional care reduced readmission or death in elderly patients admitted to hospital with heart failure. Evidence-Based Nursing. 2004;7(4):116.
4. Hustey FM, Mion LC, Connor JT, Emerman CL, Campbell J, Palmer RM. A brief risk stratification tool to predict functional decline in older adults discharged from emergency departments. Journal of the American Geriatrics Society. 2007 Aug;55(8):1269-74.
5. Jones Berkeley SB, Johnson AM, Mormer ER, Ressel K, Pastva AM, Wen F, Patterson CG, Duncan PW, Bushnell CD, Zhang S, Freburger JK. Referral to Community-Based Rehabilitation Following Acute Stroke: Findings From the COMPASS Pragmatic Trial. Circulation: Cardiovascular Quality and Outcomes. 2024 Jan;17(1):e010026.
6. Judica E, Tropea P, Bouça-Machado R, Marín M, Calarota E, Cozma L, Badea R, Ahmed M, Brach M, Ferreira JJ, Corbo M. Personalized Integrated Care Promoting Quality of Life for Older People: Protocol for a Multicenter Randomized Controlled Trial. JMIR Research Protocols. 2023 Jul 24;12(1):e47916.
7. Li P, Kang T, Carrillo-Argueta S, Kassapidis V, Grohman R, Martinez MJ, Sartori DJ, Hayes R, Jervis R, Moussa M. Bridging the gap: a resident-led transitional care clinic to improve post hospital care in a safety-net academic community hospital. BMJ Open Quality. 2024 Mar 1;13(1):e002289.
8. Rahpeima E, Bijani M, Karimi S, Alkamel A, Dehghan A. Effect of the Implementation of Interdisciplinary Discharge Planning on Treatment Adherence and Readmission in Patients Undergoing Coronary Artery Angioplasty. Investigación y Educación en Enfermería. 2022 Aug;40(2).
9. Shaw, M. C. Discharge planning and home follow up by advanced practice nurses reduced hospital readmissions of elderly patients [commentary on Naylor MD, Brooten D, Campbell R, et al. Comprehensive discharge planning and home follow-up of hospitalized elders: a randomized clinical trial. JAMA 1999 Feb 17;281(7):613-20]. Evidence Based Nursing - Volume 2, Issue 4, pp. 125 - published 1999-01-01.

### Wrong publication type (e.g., conference abstract)

1. Kansagara D, McCiain M, Englander H, Peters D, Morris CD. Patient activation measure and care transitions among socioeconomically vulnerable adults. InJOURNAL OF GENERAL INTERNAL MEDICINE 2014 Apr 1 (Vol. 29, pp. S165-S165). 233 SPRING ST, NEW YORK, NY 10013 USA: SPRINGER.
2. Liss DT, Schaeffer-Pettigrew C, Finch E, Cooper AJ, Sheth A, Tejuosho AD, Teter C, Ackermann RT. A RANDOMIZED COMPARATIVE EFFECTIVENESS TRIAL OF A TRANSITIONAL CARE CLINIC: 180-DAY EFFECTS. In JOURNAL OF GENERAL INTERNAL MEDICINE 2017 Apr 1 (Vol. 32, pp. S100-S100). 233 SPRING ST, NEW YORK, NY 10013 USA: SPRINGER.
3. Mealy B, Nevin A, Lavan A, Jariol AL, Roll V, Connor SO, Donnell DO, Cummiskey AG, McNulty J, Malley MO, Briggs R. 273 ‘HOME ON TIME’: MULTIDISCIPLINARY INTERVENTION REDUCES LENGTH OF STAY AND DELAYS IN CARE TRANSFERS ON AN ACUTE GERIATRIC MEDICINE WARD. Age and Ageing. 2022 Nov;51(Supplement_3):afac218-241.
4. Rieger EY, Kushner JN, Sriram V, Klein A, Wiklund LO, Meltzer D, Tang JW. PRIMARY CARE PROVIDER INVOLVEMENT DURING HOSPITALIZATION: PERSPECTIVES OF FREQUENTLY HOSPITALIZED PATIENTS IN THE COMPREHENSIVE CARE PROGRAM VERSUS USUAL CARE. InJOURNAL OF GENERAL INTERNAL MEDICINE 2020 Jul 1 (Vol. 35, No. SUPPL 1, pp. S241-S241). ONE NEW YORK PLAZA, SUITE 4600, NEW YORK, NY, UNITED STATES: SPRINGER.
5. Rubin DJ, Watts S, Deak A, Vaz CL, Tanner S, Recco D, Tivon M, Dillard FR, Brzana E, Joyce KE, Karunakaran A. 151-LB: A pilot randomized controlled trial to reduce hospital readmission risk of patients with diabetes: 90-day outcomes. Diabetes. 2020 Jun 1;69(Supplement_1).
6. Saunders J, Maroo S, Brownson E, Boulton Jones R. P485 An Assessment Of A Guided Self-Help and Patient Initiated Review Pathway for Ulcerative Proctitis. Journal of Crohn's and Colitis. 2023 Feb 1;17(Supplement_1):i616-.
7. Taylor SP, Bray B, Samuel P, Reed N, Morley C, Noorali A, Donaldson M, Sutaria N, Kowalkowski M. Unpacking core components of an effective sepsis transition and recovery program. InB21. SUPPORTING PATIENTS AND FAMILIES THROUGH SERIOUS ILLNESS AND RECOVERY 2023 May (pp. A2834-A2834). American Thoracic Society.
8. Teressa G, Olowo G. Assessing the Impact of Multidisciplinary Care-Transition Interventions on 30-day Readmissions at a Tertiary Care Hospital. InJOURNAL OF THE AMERICAN GERIATRICS SOCIETY 2020 Apr 1 (Vol. 68, pp. S128-S128). 111 RIVER ST, HOBOKEN 07030-5774, NJ USA: WILEY.
9. Williams NH, Roberts JL, Din NU, Charles JM, Totton N, Williams M, Mawdesley K, Hawkes CA, Morrison V, Lemmey A, Edwards RT. Developing a multidisciplinary rehabilitation package following hip fracture and testing in a randomised feasibility study: Fracture in the Elderly Multidisciplinary Rehabilitation (FEMuR). Health Technology Assessment (Winchester, England). 2017 Aug;21(44):1.
10. Yu D, Sau-Fung SF, Lee DT, Stewart S, Thompson DR, Choi KC, Yu CM. The effects of a nurse-led empowerment-based disease management program on clinical outcomes, self-care and health-related quality of life among Chinese patients with heart failure. In European Journal of Heart Failure 2014 May 1 (Vol. 16, pp. 60-60). 111 RIVER ST, HOBOKEN 07030-5774, NJ USA: WILEY-BLACKWELL.

# Appendix 2: Summarized descriptions of the interventions per study

| **Author, Year** | **Pre-discharge components** | **Post-discharge components** | **Pre- and post-discharge components** | **Case manager** | **Category*** |
| --- | --- | --- | --- | --- | --- |
| Allen, 2002 |  | - Telephone follow-up - Comprehensive needs assessment - Patient/ caregiver education - Family involvement - Home visits - Multidisciplinary treatment plan - Communication between hospital and primary care providers | - Case management | Hospital nurse practitioner | 2 |
| Altfeld, 2013 | - Comprehensive needs assessment - Multidisciplinary treatment plan | - Telephone follow-up | - Case management | Social worker | 2 |
| Atwood, 2022 | - Comprehensive needs assessment - Multidisciplinary treatment plan - Communication between hospital and primary care providers - Referral to relevant primary care providers |  | - Case management | Hospital nurse practitioner or respiratory therapist | 2 |
| Baghaei, 2021 |  | - Outpatient rehabilitation services - Hotline | - Patient/ caregiver education - Family involvement | Hospital nurse practitioner | 3 |
| Balaban, 2008 | - Comprehensive needs assessment - Multidisciplinary treatment plan - Communication between hospital and primary care providers | - Case management - Comprehensive needs assessment - Telephone follow-up |  | Hospital nurse practitioner | 2 |
| Balaban, 2015 |  | - Case management - Communication between hospital and primary care providers - Telephone follow-up |  | Trained patient navigator, not further specified | 2 |
| Balaban, 2017 |  | - Case management - Communication between hospital and primary care providers - Telephone follow-up |  | Trained patient navigator, not further specified | 2 |
| Casas, 2006 | - Comprehensive needs assessment - Patient education on self-management strategies - Multidisciplinary treatment plan | - Home visits - Telephone follow-up - Hotline | - Case management - Communication between hospital and primary care providers | Hospital nurse practitioner | 2 |
| Coskun, 2021 | - Comprehensive needs assessment - Multidisciplinary treatment plan | - Home visits - In-home rehabilitation | - Case management | Hospital nurse practitioner | 3 |
| Courtney, 2009 | - Comprehensive needs assessment - Multidisciplinary treatment plan | - Home visits - Telephone follow-up | - Case management - Family involvement | Hospital nurse practitioner | 2 |
| Davidson, 2009 | - Comprehensive needs assessment - Multidisciplinary treatment plan | - Telephone follow-up - Outpatient rehabilitation services - Patient education on self-management strategies - Home visits - In-home rehabilitation | - Case management | Hospital nurse practitioner | 3 |
| Del Sindaco, 2007 | - Comprehensive needs assessment - Multidisciplinary treatment plan - Communication between hospital and primary care providers | - Outpatient rehabilitation services - Telephone follow-up - Home visits | - Case management | Hospital physician | 2 |
| Deng, 2021 | - Comprehensive needs assessment - Multidisciplinary treatment plan | - Telephone follow-up - Outpatient rehabilitation services - Home visits - In-home rehabilitation | - Case management - Communication between hospital and primary care providers | Primary care nurse practitioner | 3 |
| Dhalla, 2014 | - Comprehensive needs assessment - Multidisciplinary treatment plan | - Hotline | - Case management - Communication between hospital and primary care providers | Care coordinator, not further specified | 2 |
| Donnelly, 2004 |  | - Comprehensive needs assessment - Multidisciplinary treatment plan - Home visits - In-home rehabilitation - Family involvement | - Case management | Primary care coordinator, not further specified | 3 |
| Evangelista, 2023 | - Comprehensive needs assessment - Multidisciplinary treatment plan - Family involvement - Communication between hospital and primary care providers - Referral to relevant primary care providers |  |  | NA | 1 |
| Evans, 1993 | - Comprehensive needs assessment - Multidisciplinary treatment plan - Family involvement - Communication between hospital and primary care providers - Referral to relevant primary care providers |  |  | NA | 1 |
| Finkelstein, 2020 | - Comprehensive needs assessment - Multidisciplinary treatment plan - Communication between hospital and primary care providers | - Home visits | - Case management | Hospital multidisciplinary team | 2 |
| Finlayson, 2018 | - Comprehensive needs assessment - Multidisciplinary treatment plan | - Home visits - In-home rehabilitation - Telephone follow-up | - Case management | Hospital multidisciplinary team | 3 |
| Fjaertoft, 2003 | - Comprehensive needs assessment - Multidisciplinary treatment plan | - Outpatient rehabilitation services - Home visits - In-home rehabilitation - Communication between hospital and primary care providers | - Case management | Hospital multidisciplinary team | 3 |
| Fjaertoft, 2011 | - Comprehensive needs assessment - Multidisciplinary treatment plan | - Outpatient rehabilitation services - In-home rehabilitation - Home visits - In-home rehabilitation - Communication between hospital and primary care providers | - Case management | Hospital multidisciplinary team | 3 |
| Garcia-Aymerich, 2007 | - Communication between hospital and primary care providers - Patient education on self-management strategies | - Hotline - Home visits - Telephone follow-up | - Case management - Patient education on self-management strategies | Hospital nurse practitioner | 2 |
| Hofstad, 2014 | - Comprehensive needs assessment - Multidisciplinary treatment plan | - Home visits - In-home rehabilitation - Outpatient rehabilitation services | - Communication between hospital and primary care providers | Hospital multidisciplinary team | 3 |
| Hu, 2020 | - Comprehensive needs assessment - Multidisciplinary treatment plan | - Telephone follow-up - Online chat platform (for patient -case manager communication) | - Case management | Hospital nurse practitioner | 2 |
| Hung, 2023 | - Comprehensive needs assessment - Multidisciplinary treatment plan - Family involvement - Patient education on self-management strategies | - Telephone follow-up | - Case management | Hospital nurse practitioner | 2 |
| Jack, 2009 | - Comprehensive needs assessment - Multidisciplinary treatment plan - Communication between hospital and primary care providers | - Telephone follow-up - Medication review | - Case management | Hospital nurse practitioner | 2 |
| Jackson, 2012 | - Comprehensive needs assessment - Multidisciplinary treatment plan | - Home visits - In-home rehabilitation - Telephone/video calls follow-up - In-home rehabilitation | - Case management | Hospital multidisciplinary team | 3 |
| Jepma, 2021 | - Comprehensive needs assessment - Multidisciplinary treatment plan | - Home visits - In-home rehabilitation | - Case management | Hospital multidisciplinary team | 3 |
| Ko, 2016 | - Comprehensive needs assessment - Multidisciplinary treatment plan | - Outpatient rehabilitation services - Home visits - In-home rehabilitation | - Case management | Hospital multidisciplinary team | 3 |
| Lainscak, 2013 | - Comprehensive needs assessment - Multidisciplinary treatment plan - Communication between hospital and primary care providers | - Telephone follow-up - Home visits | - Case management - Family involvement | Discharge coordinator, not further specified | 2 |
| Lanzeta, 2016 | - Comprehensive needs assessment - Multidisciplinary treatment plan - Communication between hospital and primary care providers |  | - Case management | Hospital nurse practitioner | 2 |
| Latour, 2006 |  | - Comprehensive needs assessment - Multidisciplinary treatment plan - Communication between hospital and primary care providers - Telephone follow-up - Home visits | - Case management | Case manager, not further specified | 2 |
| Lim, 2003 | - Comprehensive needs assessment - Multidisciplinary treatment plan | - Telephone follow-up | - Case management - Communication between hospital and primary care providers | Hospital allied health professional or nurse practitioner | 2 |
| Linden, 2014 | - Patient education on self-management strategies - Multidisciplinary treatment plan - Medication review | - Telephone follow-up - Hotline - Symptom monitoring via interactive voice response - Communication between hospital and primary care providers | - Case management | Hospital nurse practitioner | 2 |
| Liu, 2023 | - Multidisciplinary treatment plan | - Telephone follow-up | - Case management - Comprehensive needs assessment | Hospital nurse practitioner | 2 |
| Markle-Reid, 2021 |  | - Case management - Telephone follow-up - Virtual multidisciplinary home visits - Comprehensive needs assessment - Multidisciplinary treatment plan - Medication review - Patient education on self-management strategies - Family involvement - Communication platform for hospital providers - Communication between hospital and primary care providers |  | Physiotherapist or occupational therapist | 2 |
| Markle-Reid, 2023 |  | - Home visits - Telephone follow-up - Comprehensive needs assessment - Multidisciplinary treatment plan - Medication review - Patient education on self-management strategies | - Case management | Hospital nurse practitioner | 3 |
| McCorkle, 2000 |  | - Home visits - Comprehensive needs assessment - Multidisciplinary treatment plan - Communication between hospital and primary care providers - Telephone follow-up | - Case management | Hospital nurse practitioner | 2 |
| Meyer, 2022 | - Comprehensive needs assessment - Multidisciplinary treatment plan - Referral to relevant primary care providers |  |  | NA | 1 |
| Naylor, 2004 | - Multidisciplinary treatment plan |  | - Case management - Comprehensive needs assessment - Communication between hospital and primary care providers - Patient education on self-management strategies - Family involvement | Hospital nurse practitioner | 2 |
| Preen, 2005 | - Comprehensive needs assessment - Multidisciplinary treatment plan - Referral to relevant primary care providers |  |  | NA | 1 |
| Rich, 1993 | - Comprehensive needs assessment - Multidisciplinary treatment plan | - Telephone follow-up - Home visits | - Case management | Hospital nurse practitioner | 2 |
| Santana, 2017 | - Comprehensive needs assessment - Multidisciplinary treatment plan - Referral to relevant primary care providers |  |  | NA | 1 |
| Schnipper, 2021 | - Comprehensive needs assessment - Multidisciplinary treatment plan |  | - Case management - Communication between hospital and primary care providers | Hospital nurse practitioner and primary care professional (e.g., nurse practitioner) | 2 |
| Thorsen, 2005 | - Comprehensive needs assessment - Multidisciplinary treatment plan | - In-home rehabilitation |  | Hospital multidisciplinary team | 3 |
| Thygesen, 2015 |  | - Case management - Home visits - Comprehensive needs assessment - Multidisciplinary treatment plan |  | Primary care physician and nurse practitioner | 2 |
| Van Spall, 2019 | - Comprehensive needs assessment - Multidisciplinary treatment plan - Communication between hospital and primary care providers - Referral to relevant primary care providers |  | - Case management | Hospital nurse practitioner | 2 |
| Visperas, 2021 |  | - Telephone follow-up | - Patient-provider internet communication platform - Patient education on self-management strategies - Symptoms monitoring via communication platform - Case management | Care team, not further specified | 2 |
| Zimmerman, 2021 | - Comprehensive needs assessment - Multidisciplinary treatment plan |  | - Case management - Communication between hospital and primary care providers | Hospital nurse practitioner | 2 |

NA: Not applicable

* Interventions were organized into 3 categories according to their content. 1: Development of a care plan and referral to primary care providers; 2: Similar interventions to category 1 with, in addition, a case manager coordinating patient care within and between settings; 3: Entire transitional care provided by a dedicated multidisciplinary team in-hospital and post-discharge

# Appendix 3: Meta-analyses per outcome: Forest plots, funnel plots, and subgroup analyses

*Statistically significant effects

## Hospital readmission rates (up to 2 years after hospital discharge)

### Forest plot overall analysis


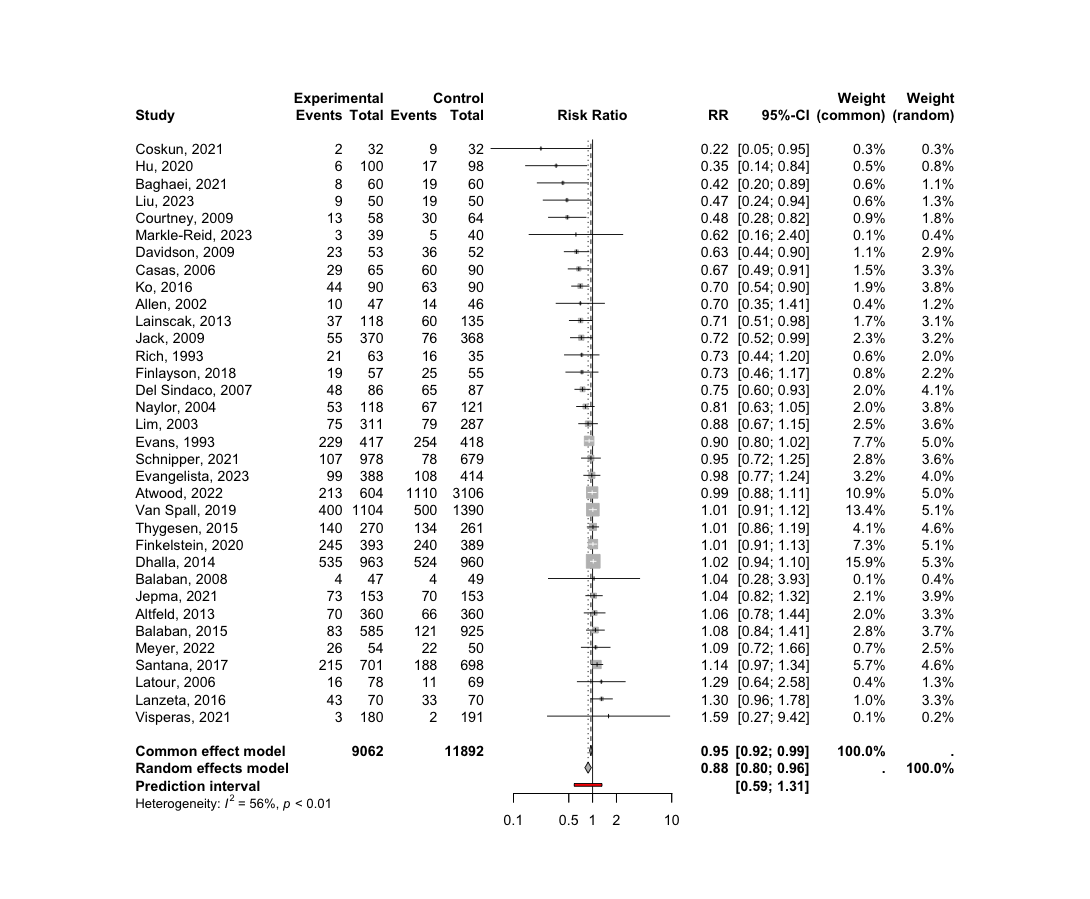


### Funnel plot


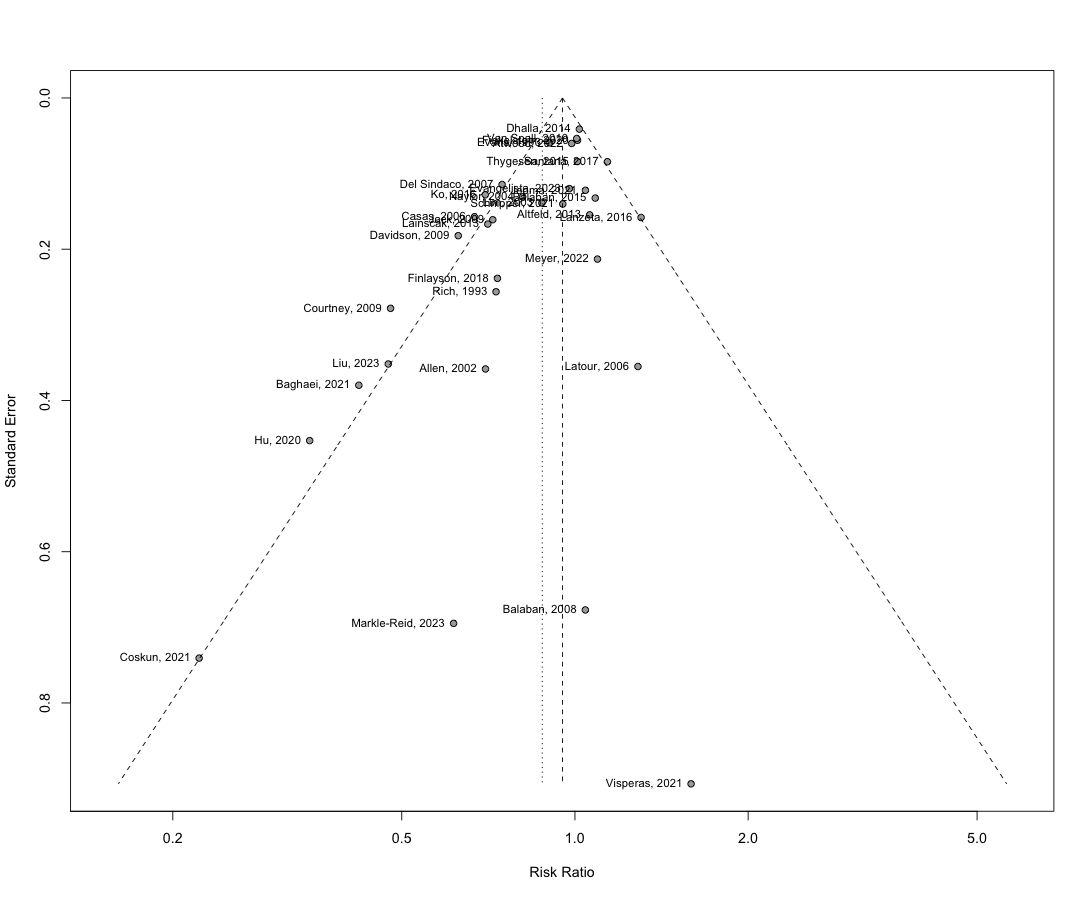


Eggers' test of the intercept

P= 0.001

Eggers' test indicates the presence of funnel plot asymmetry.

### Subgroup analysis excluding high-risk-of-bias studies


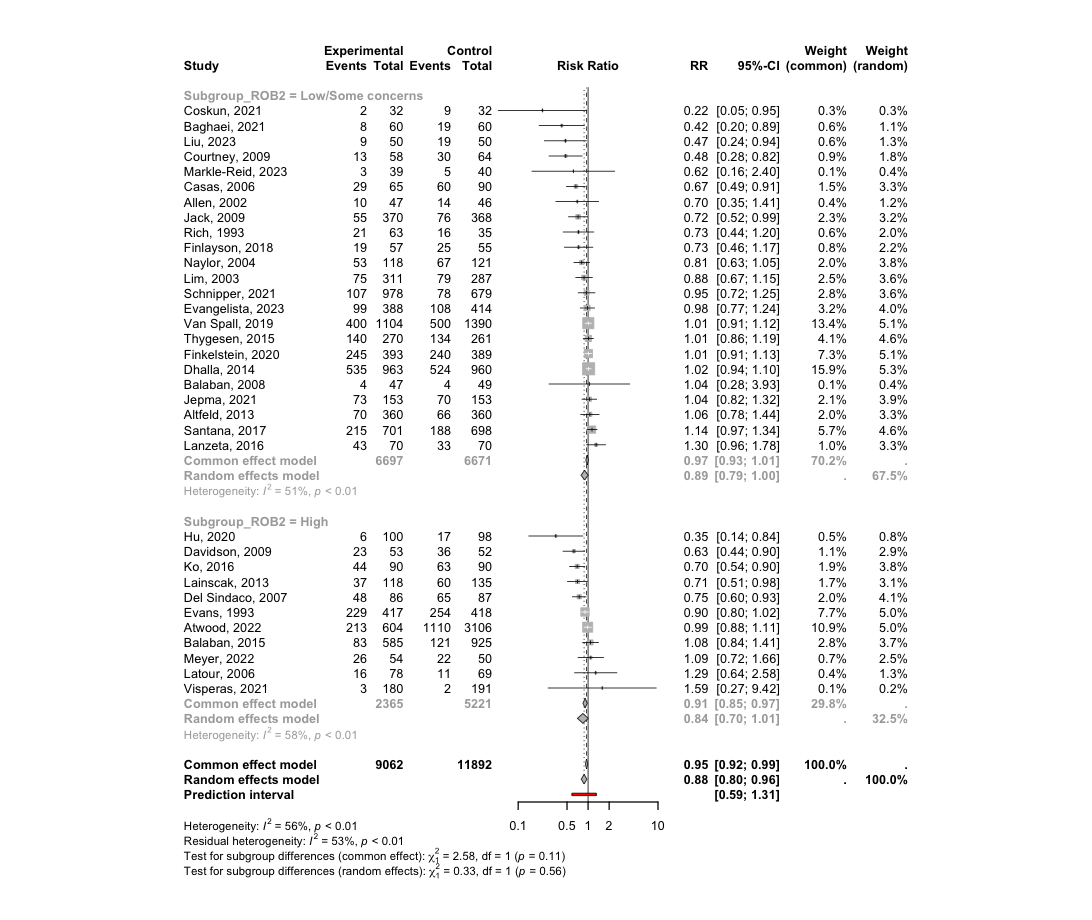


### Subgroup analysis per intervention type


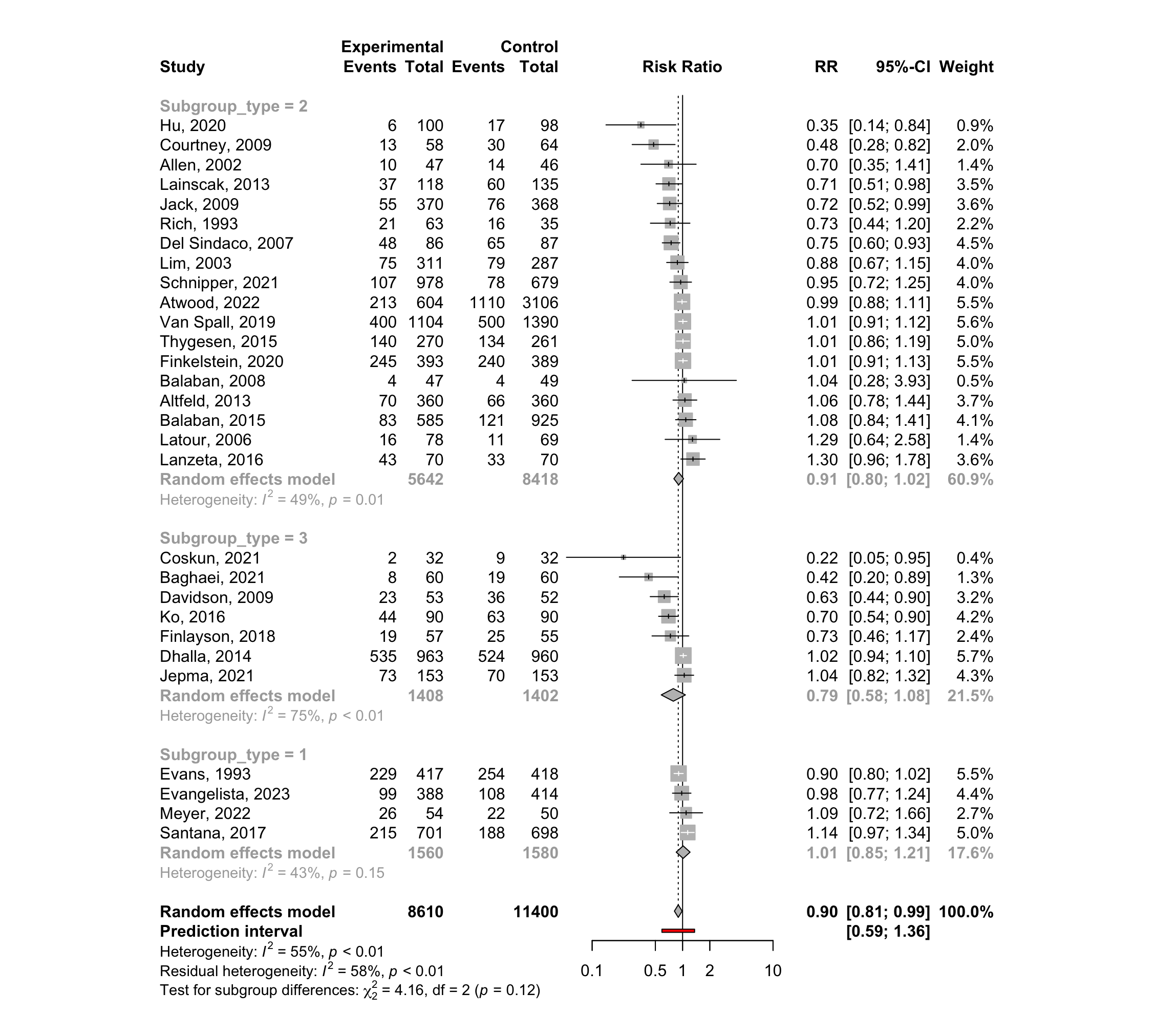


## Mortality rates (up to 44 months after hospital discharge)

### Forest plot overall analysis


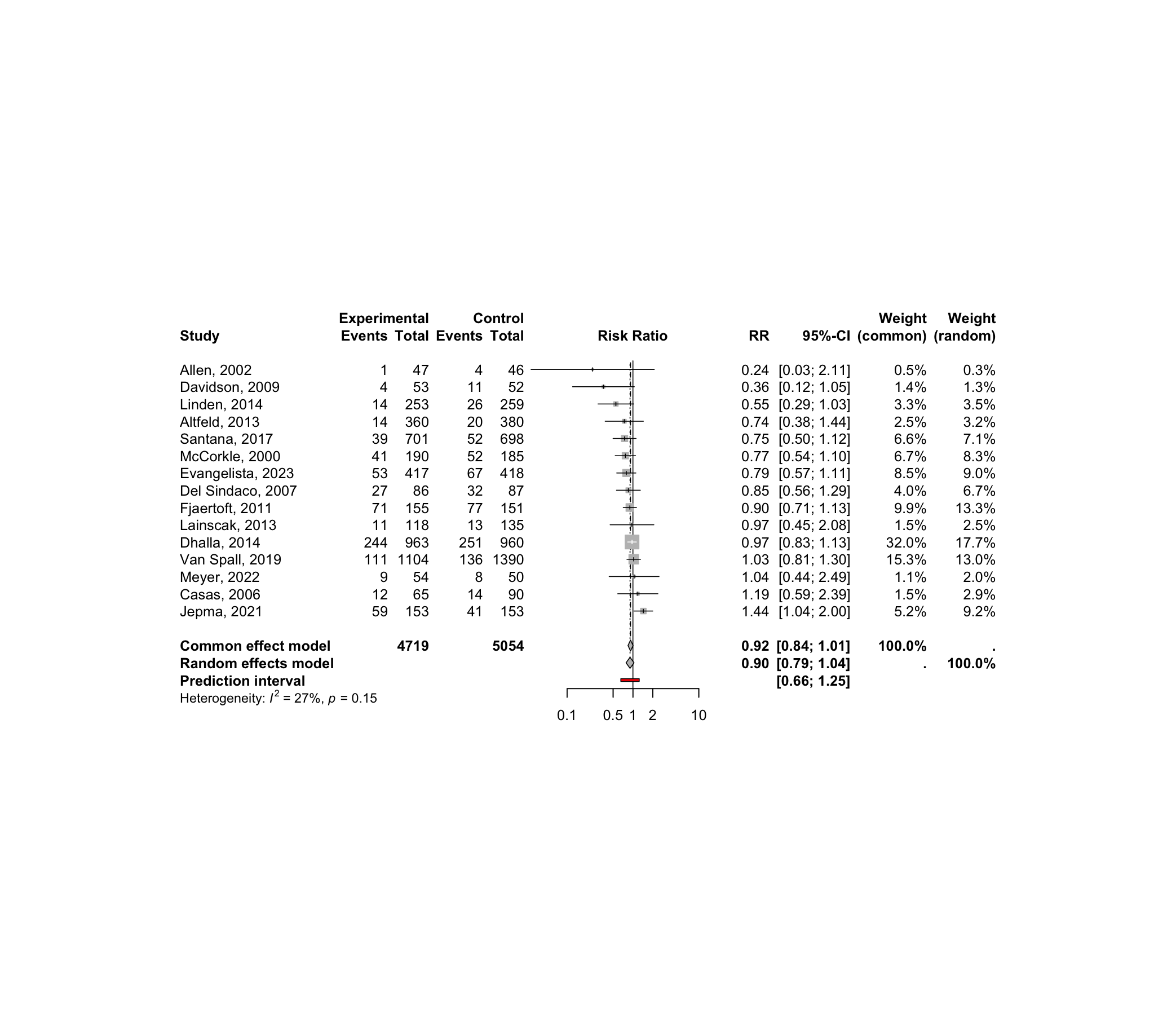


### Funnel plot


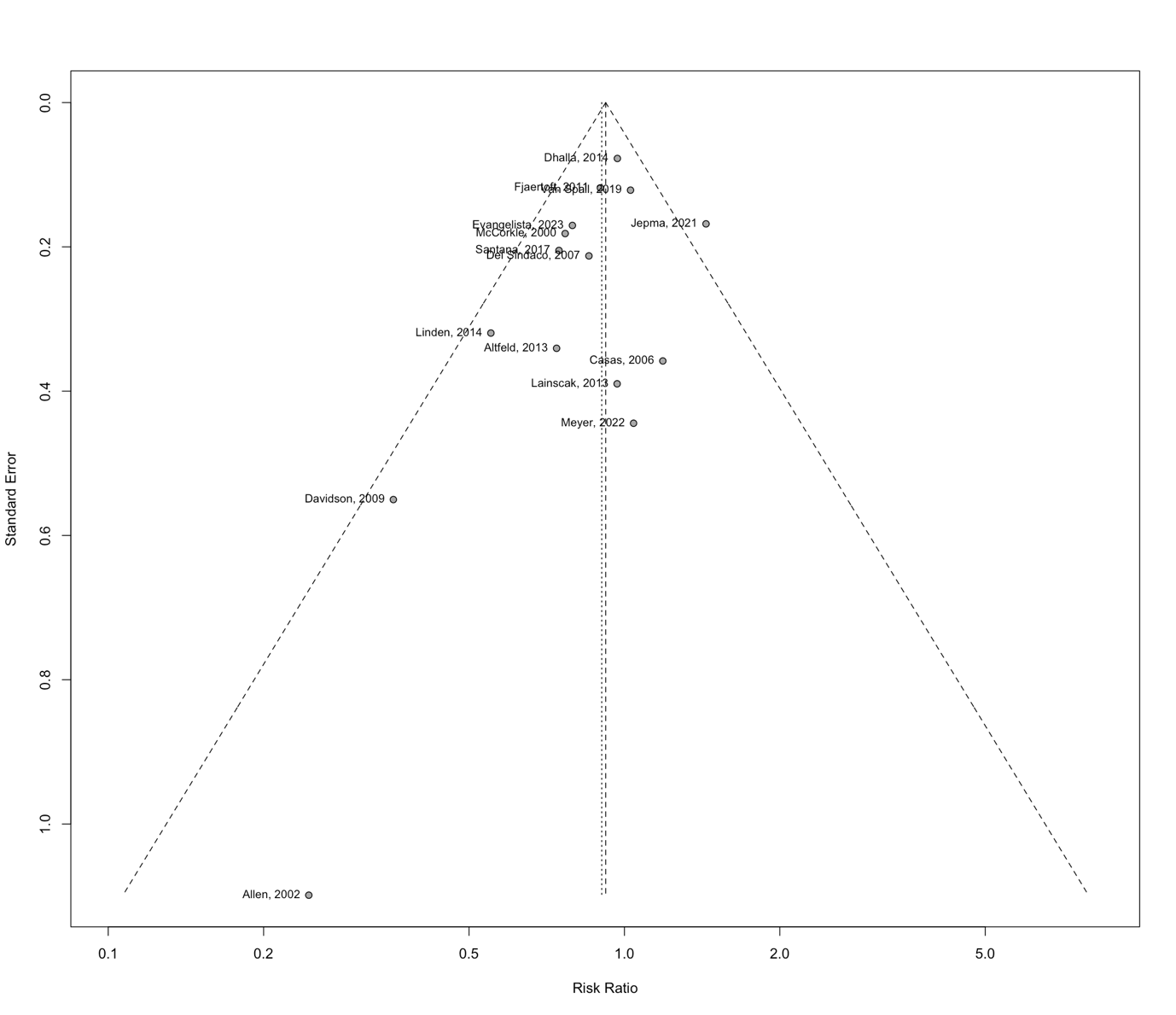


Eggers' test of the intercept

P= 0.101

Eggers' test does not indicate the presence of funnel plot asymmetry.

### Subgroup analysis excluding high-risk-of-bias studies


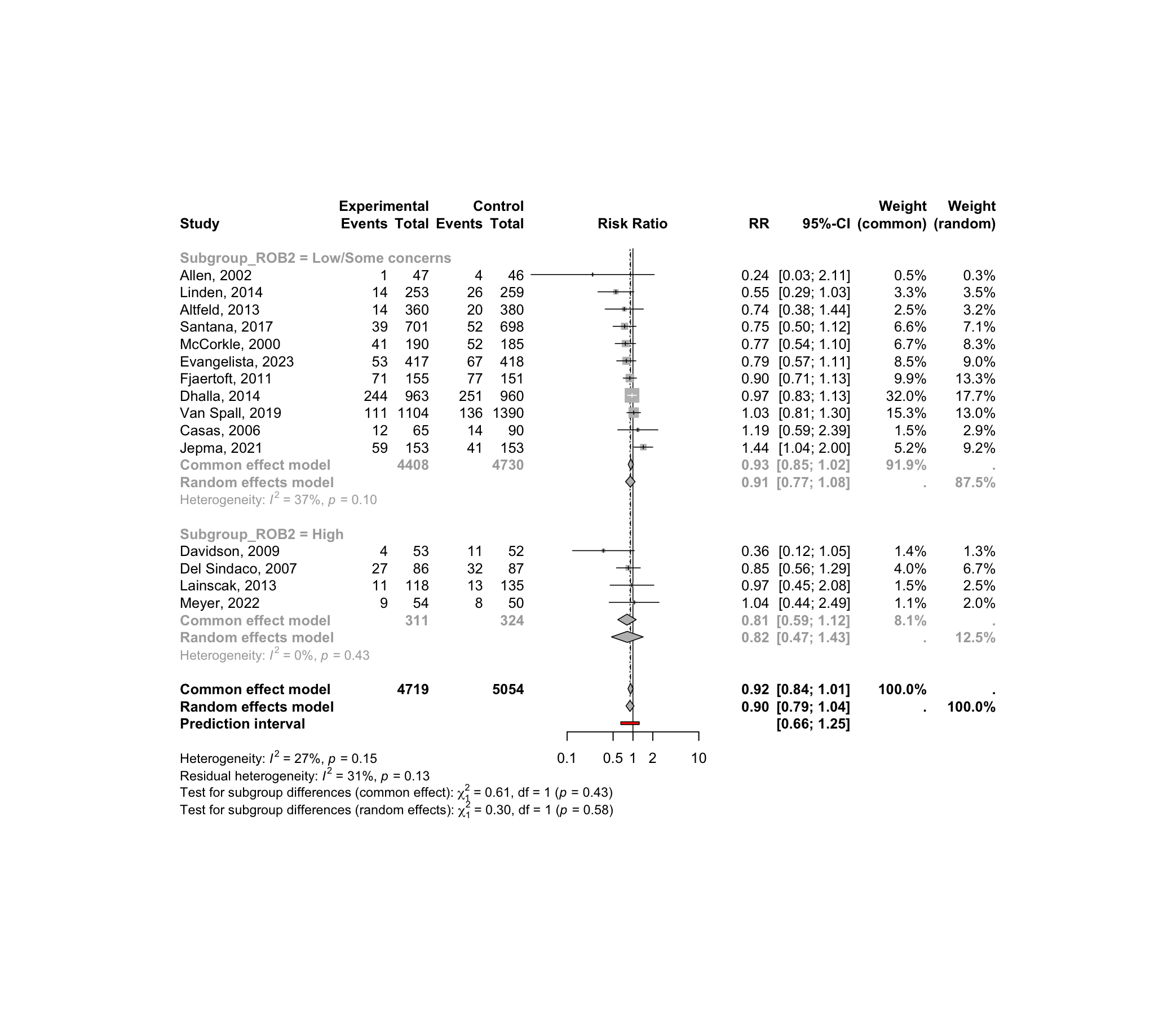


### Subgroup analysis per intervention type


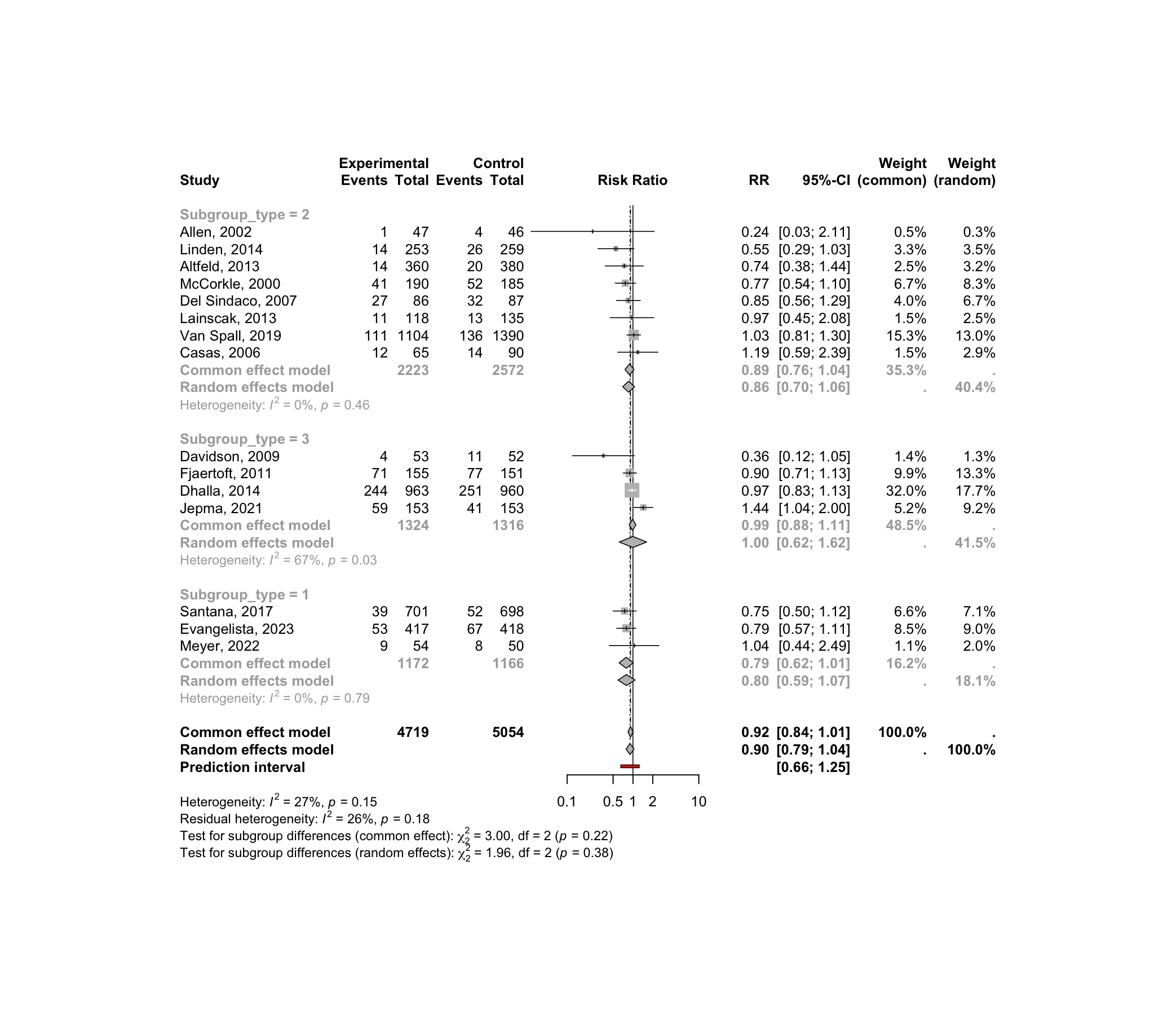


## Emergency department visit rates (up to 1 year after hospital discharge)

### Forest plot overall analysis


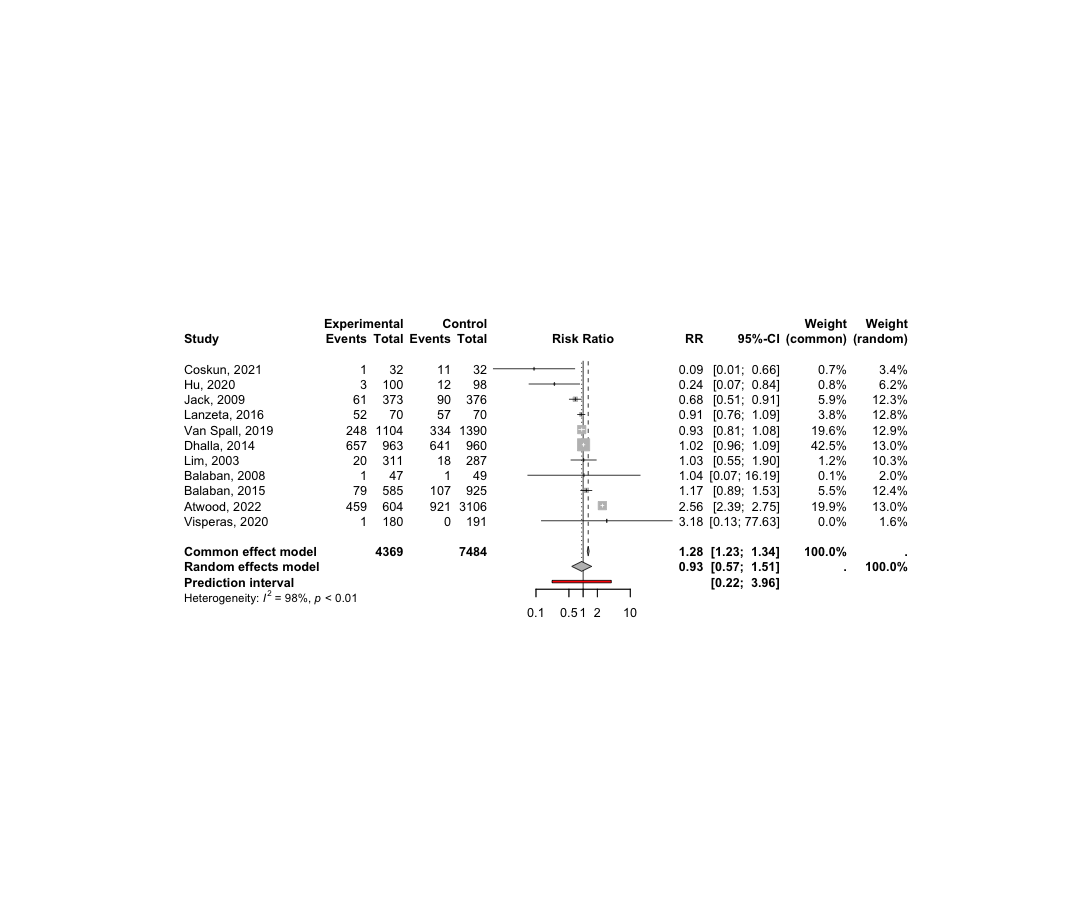


### Funnel plot


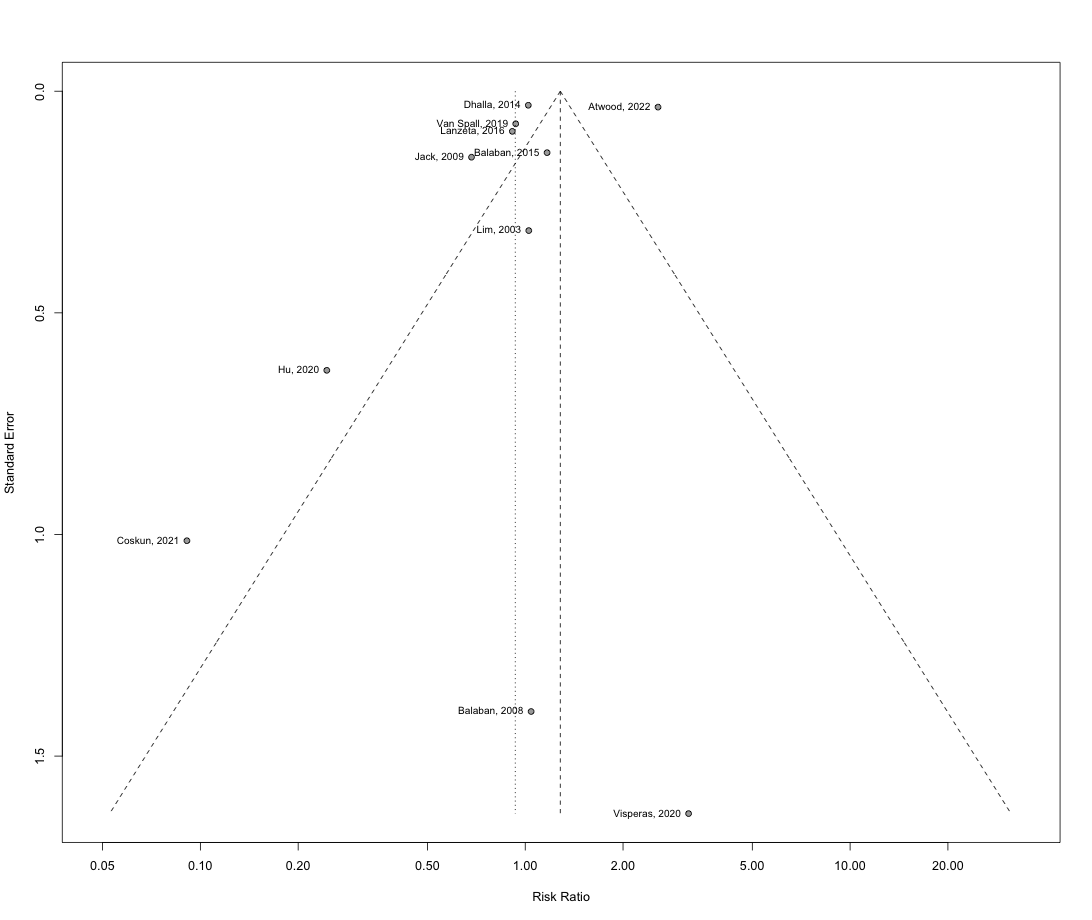


Eggers' test of the intercept

P= 0.419

Eggers' test does not indicate the presence of funnel plot asymmetry.

### Subgroup analysis excluding high-risk-of-bias studies


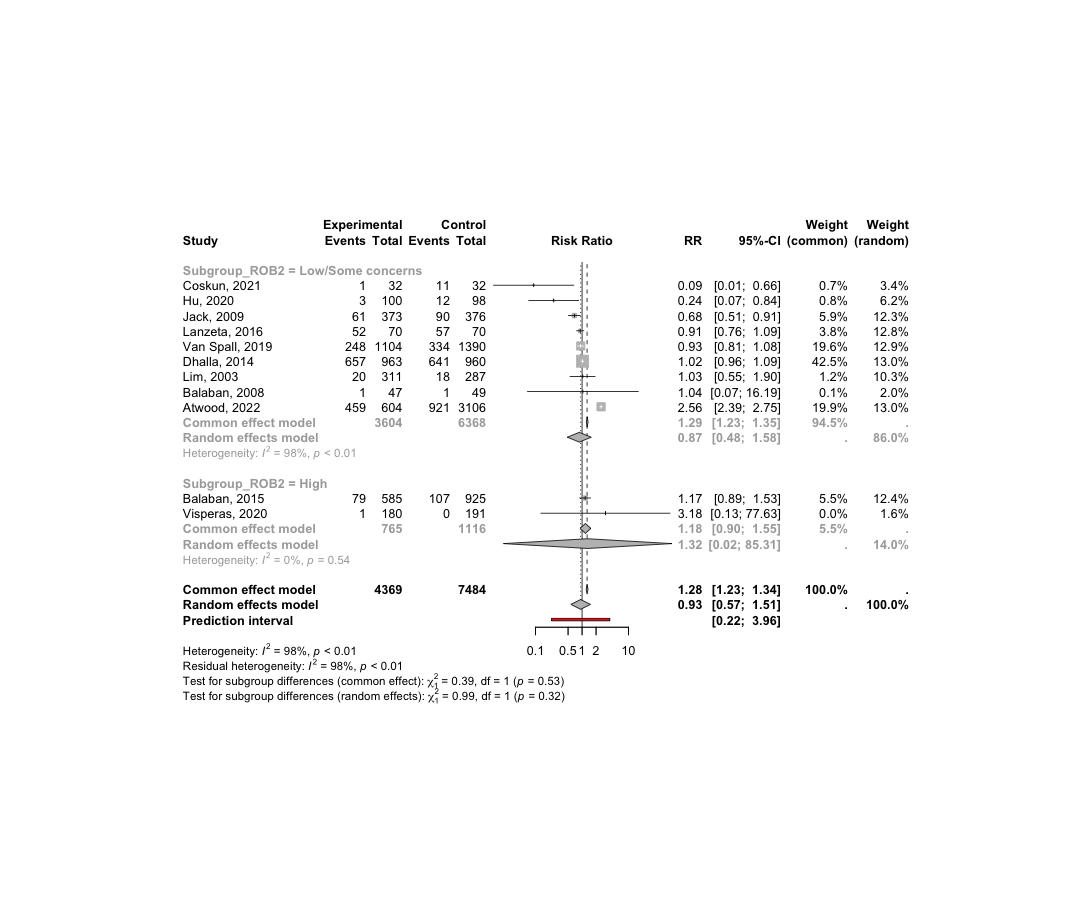


### Subgroup analysis per intervention type

**
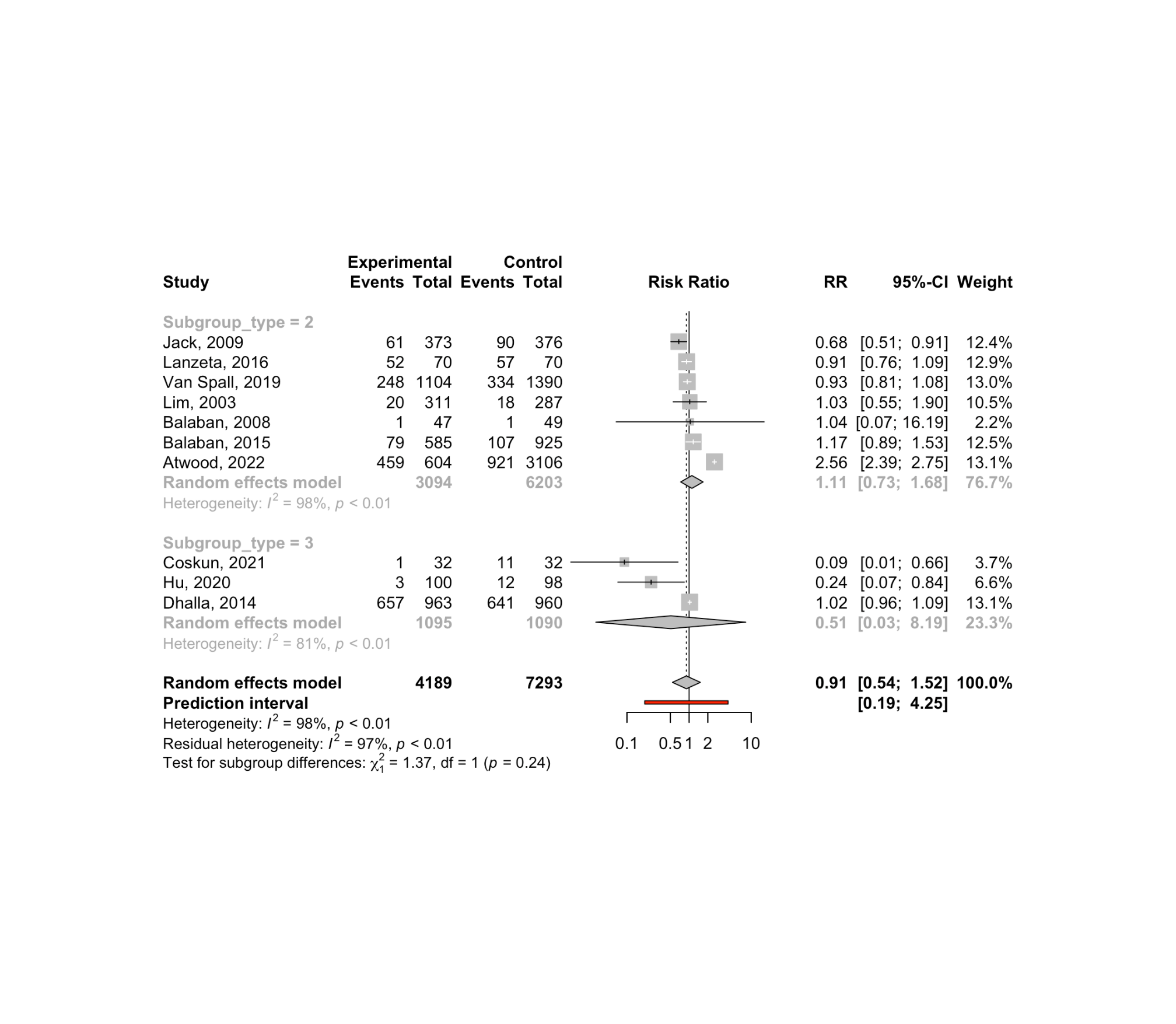
**

## Health-related quality of life

### Forest plot overall analysis

**
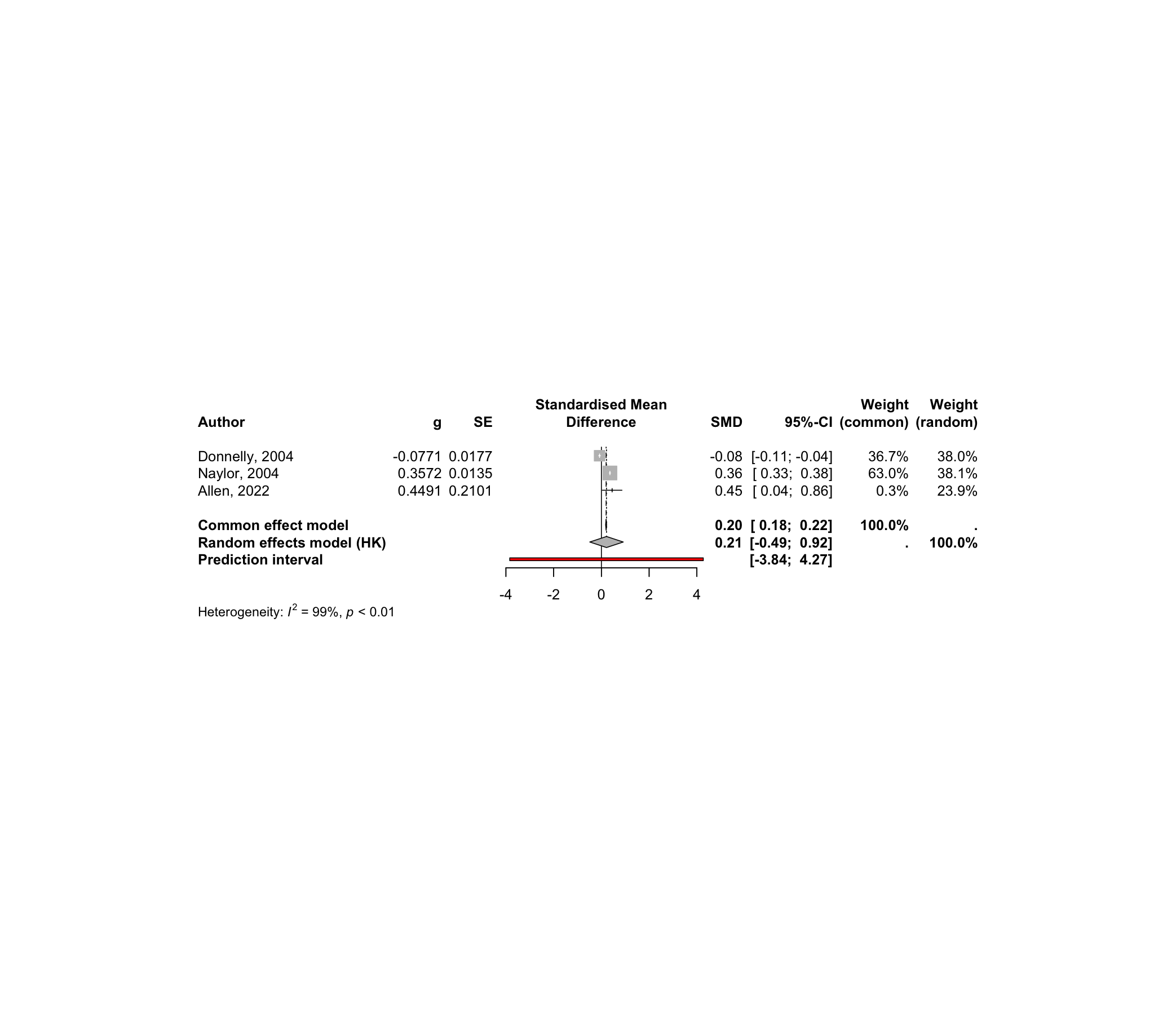
**

### Funnel plot


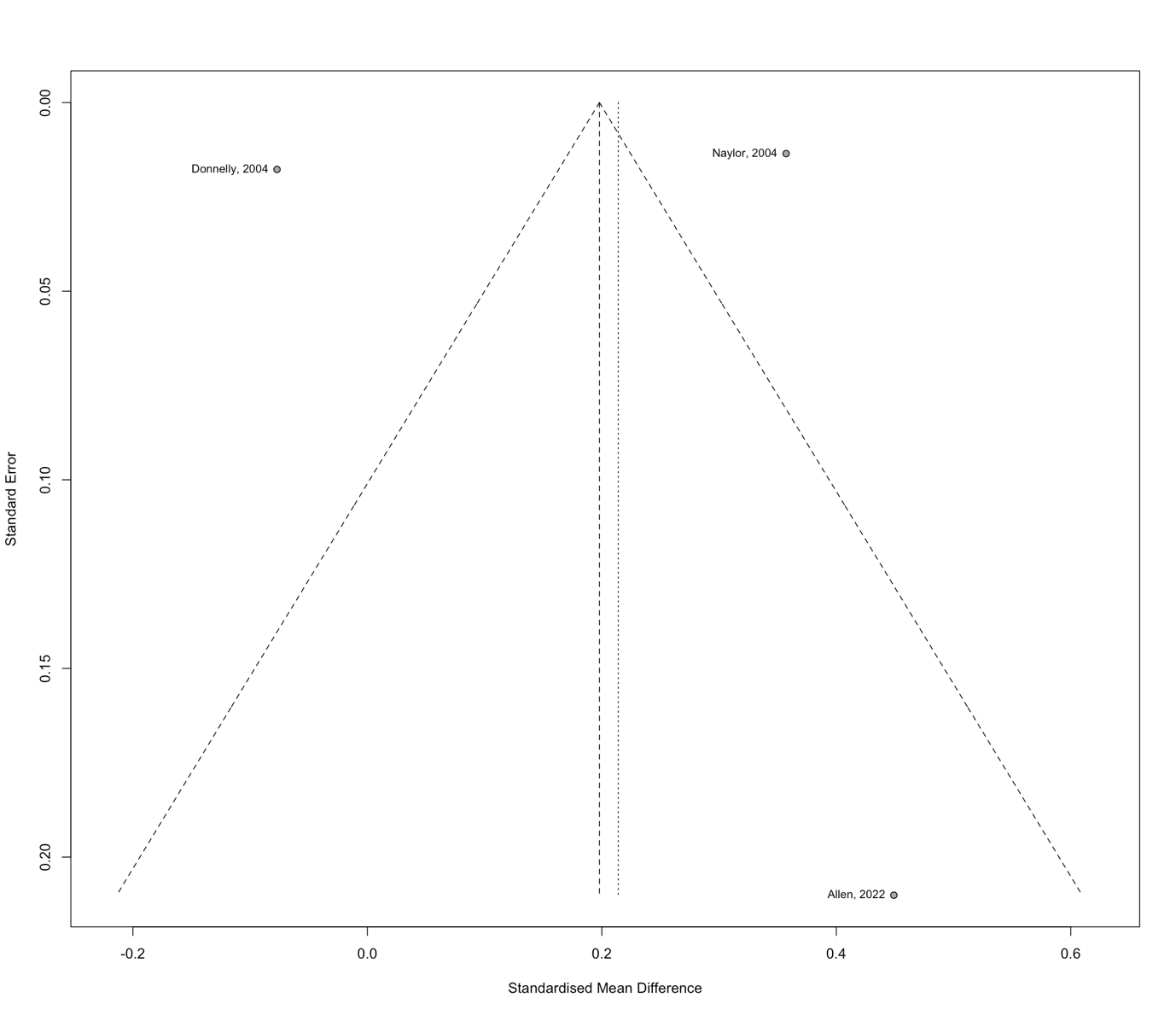


Eggers' test of the intercept

P= 0.911

Eggers' test does not indicate the presence of funnel plot asymmetry.

### Subgroup analysis excluding high-risk-of-bias studies

**
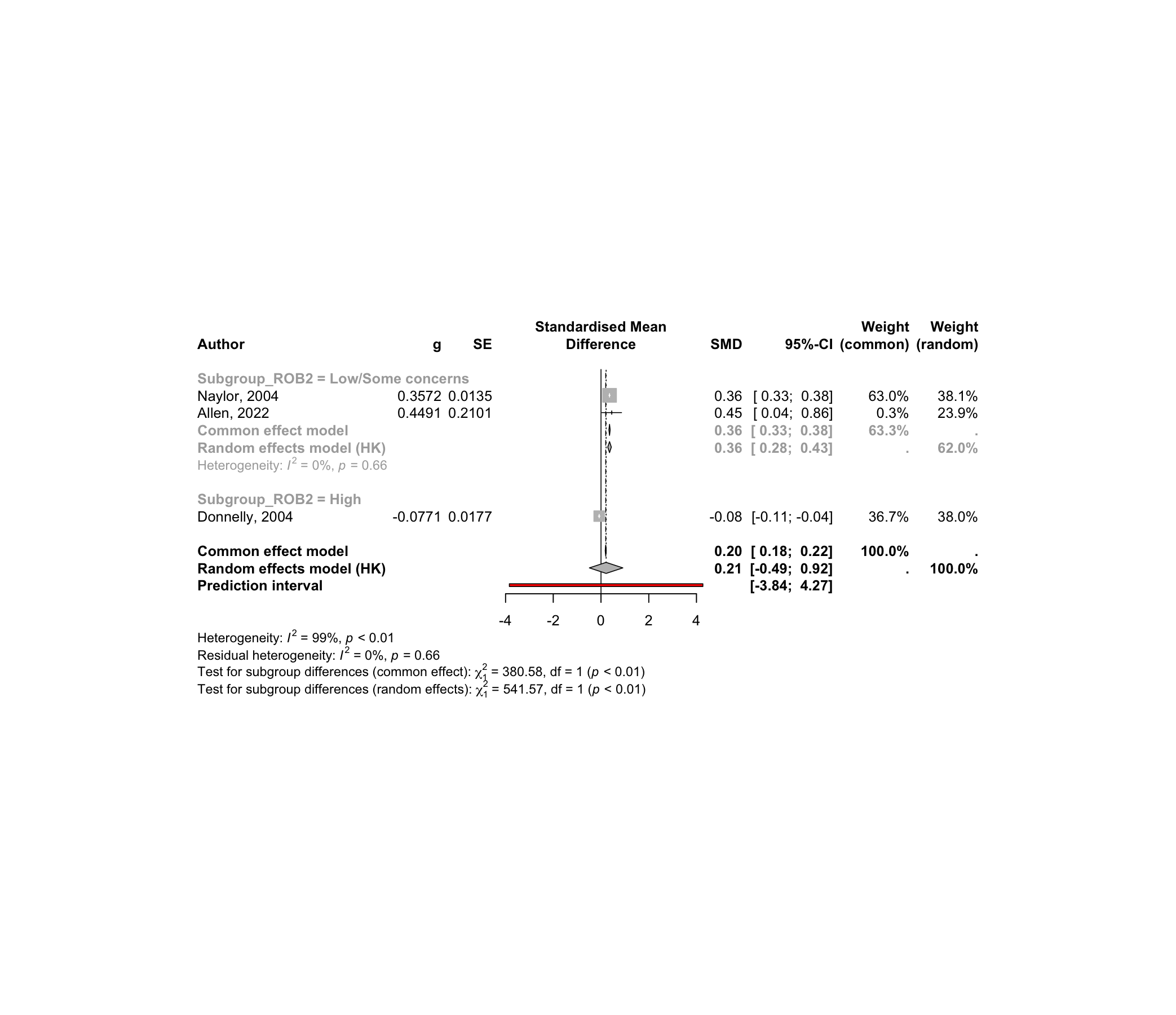
**

### Subgroup analysis per intervention type:


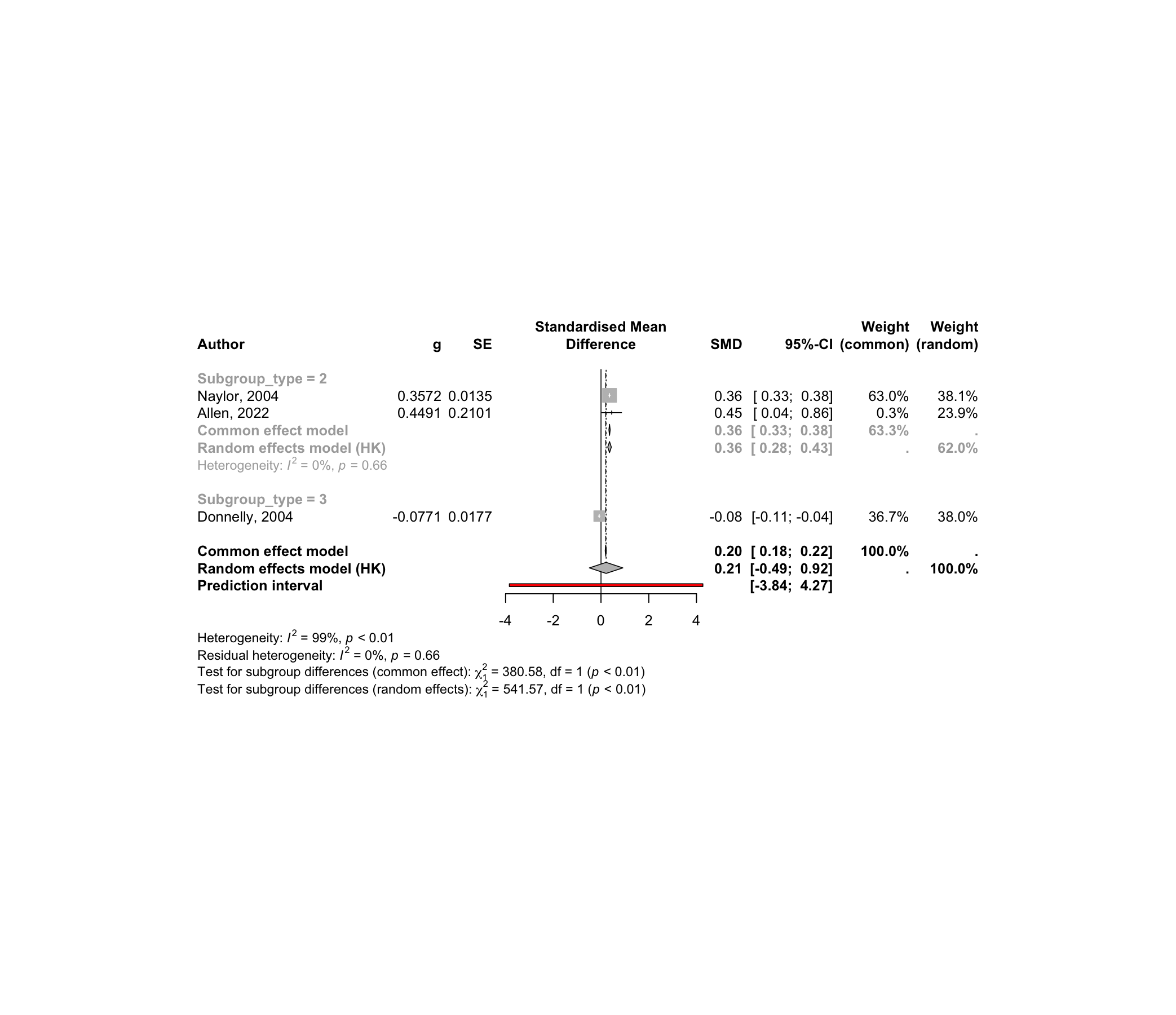


## Physical quality of Life

### Forest plot overall analysis


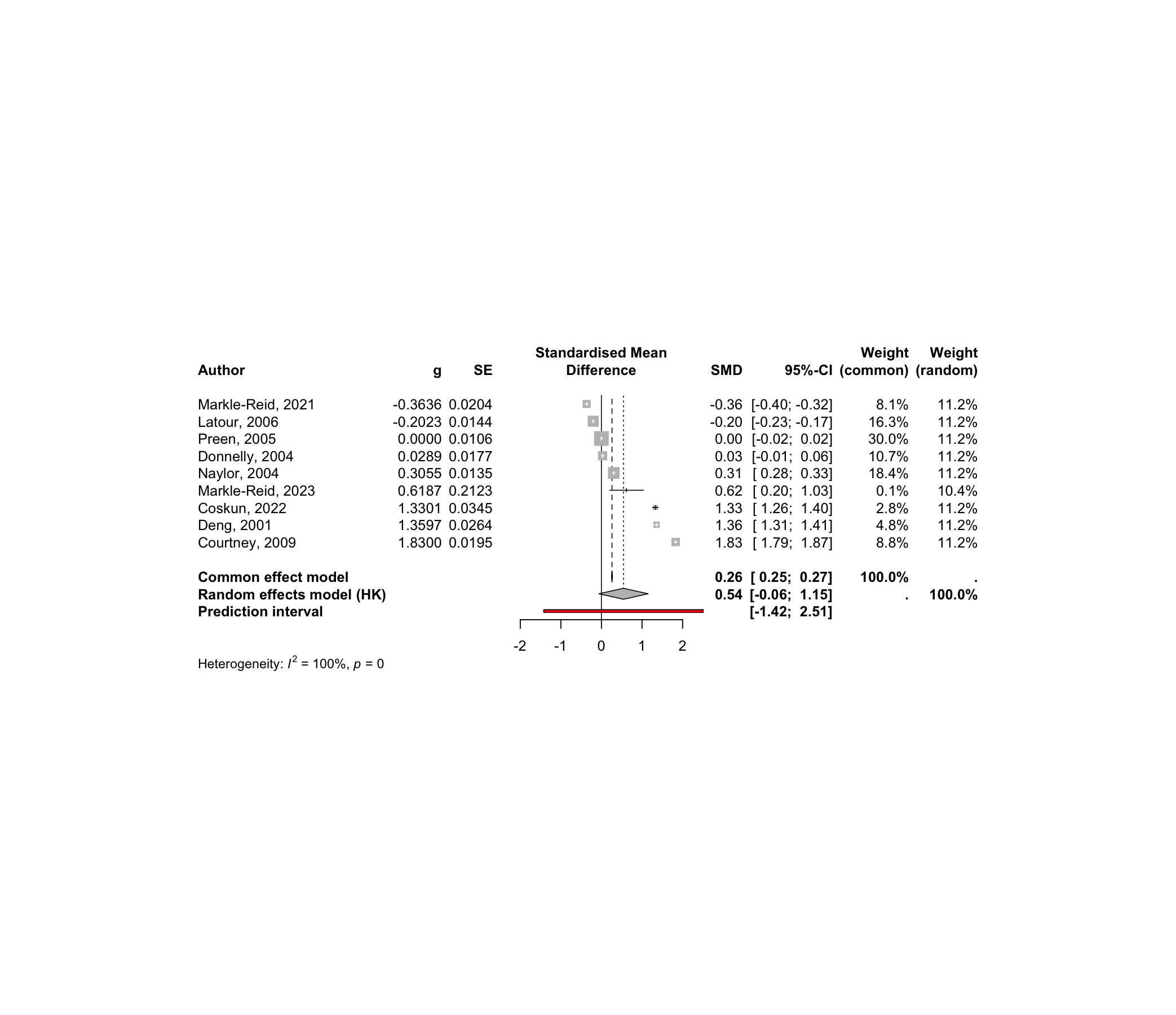


### Funnel plot


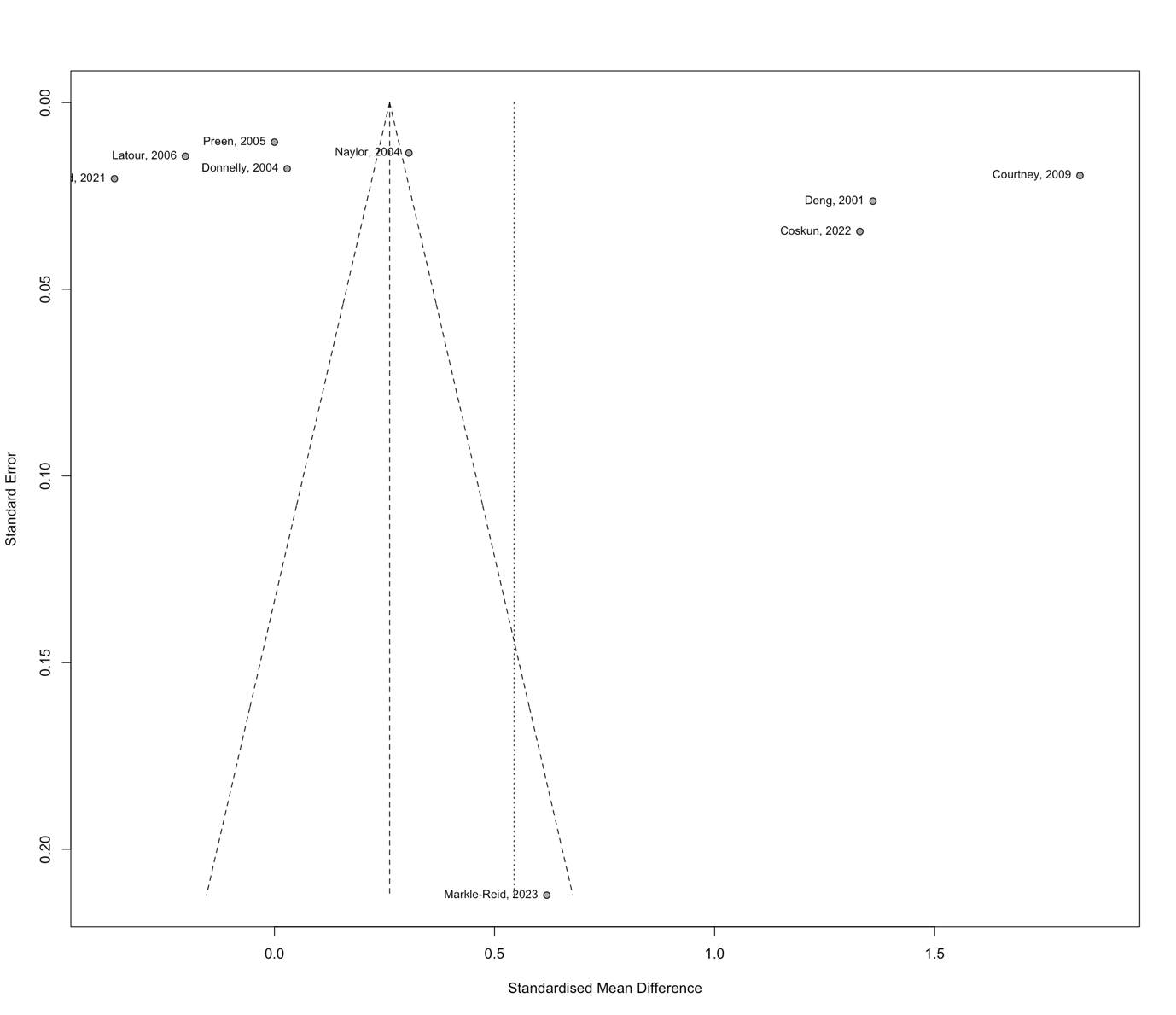


Eggers' test of the intercept

P= 0.285

Eggers' test does not indicate the presence of funnel plot asymmetry.

### Subgroup analysis excluding high-risk-of-bias studies


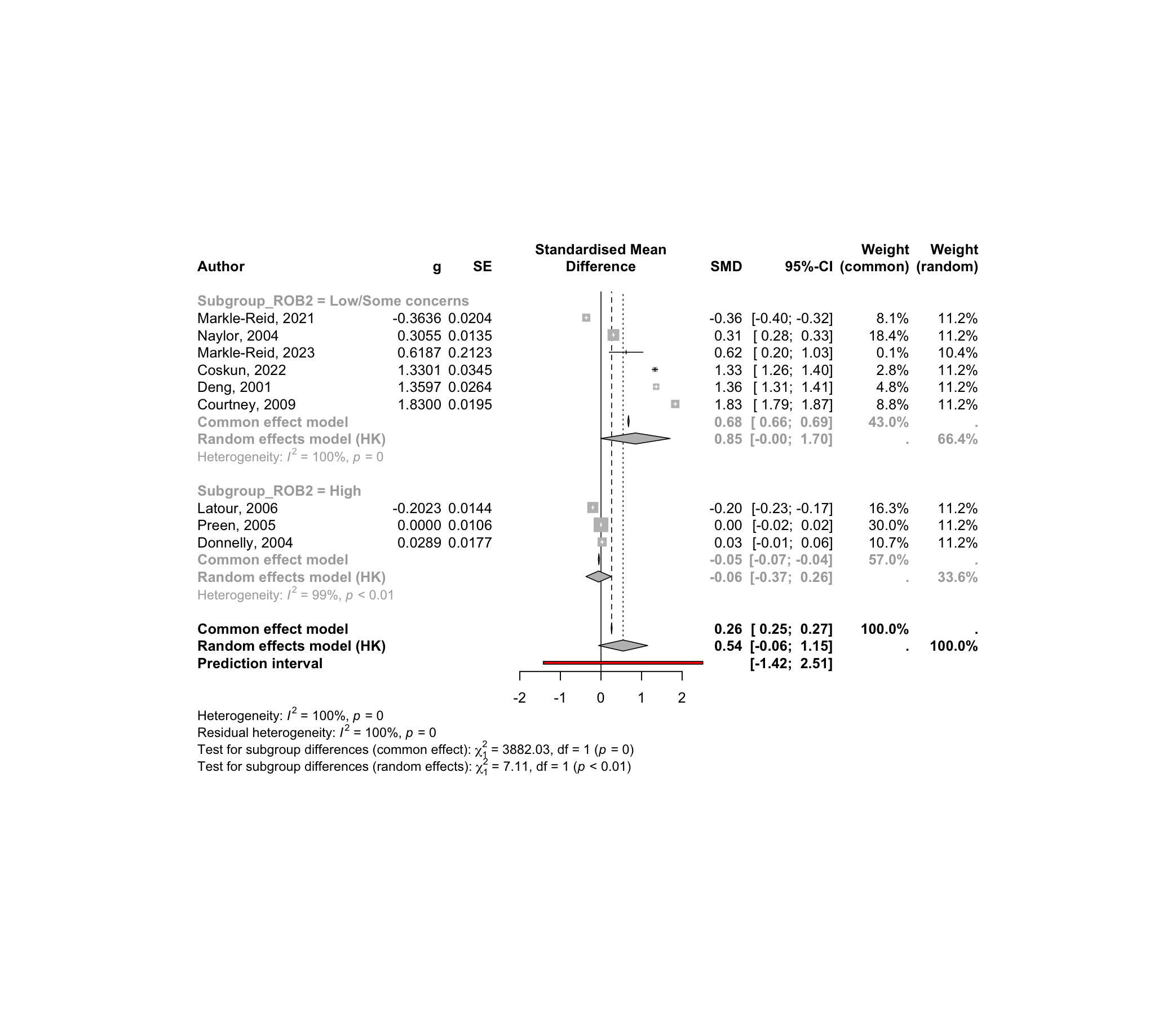


### Subgroup analysis per intervention types


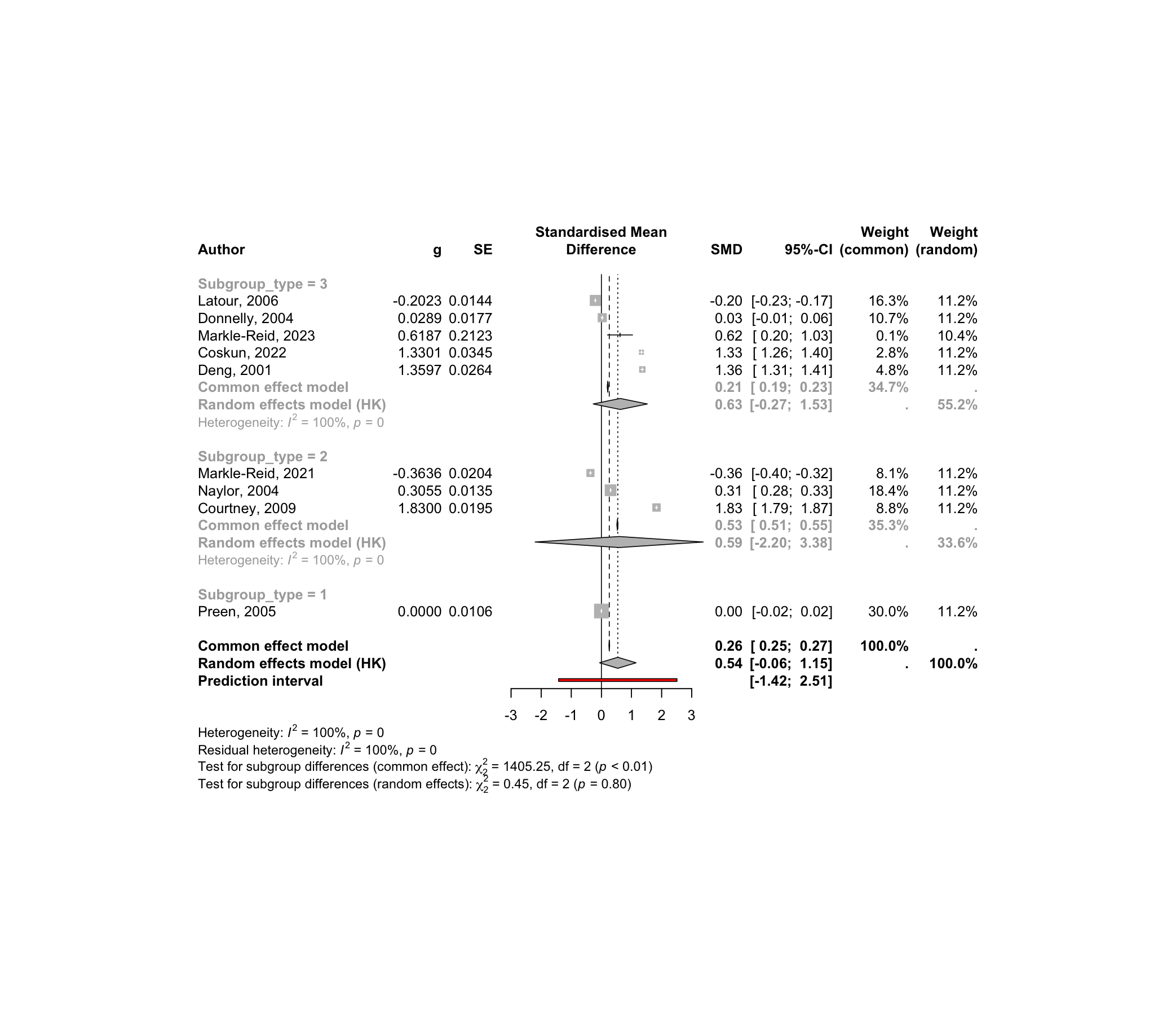


## Mental quality of life

### Forest plot overall analysis


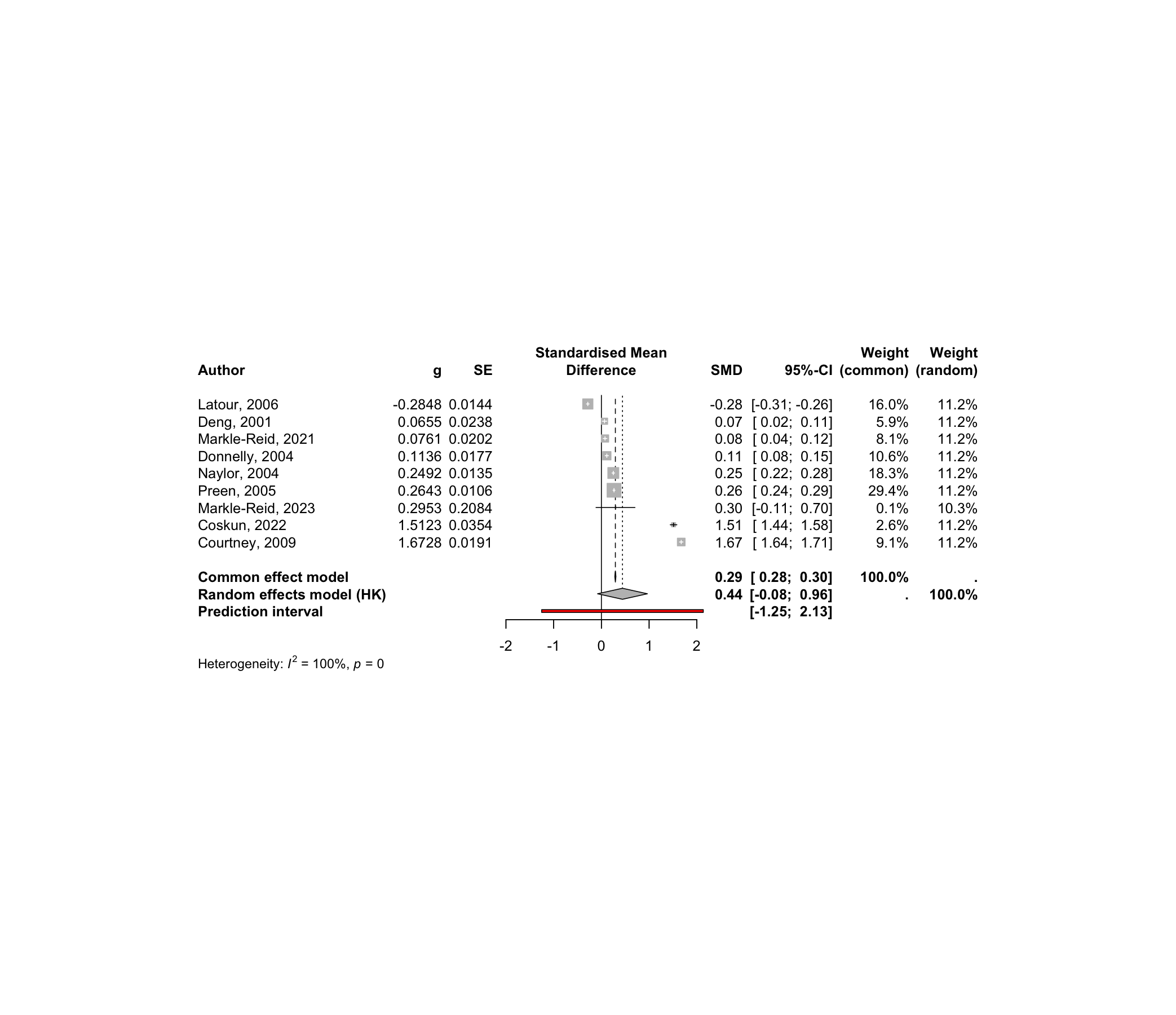


### Funnel plot


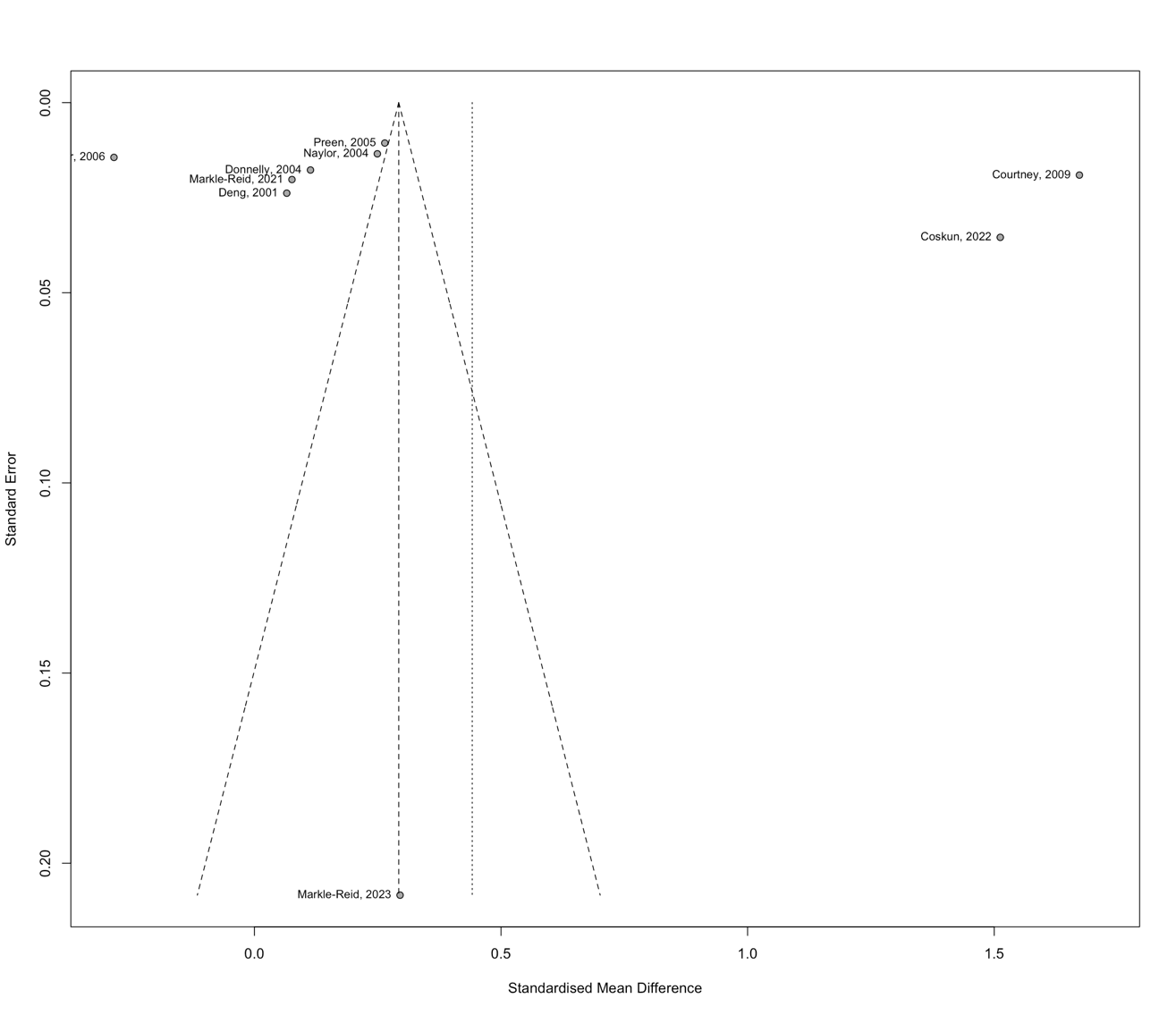


Eggers' test of the intercept

P= 0.497

Eggers' test does not indicate the presence of funnel plot asymmetry.

### Subgroup analysis excluding high-risk-of-bias studies


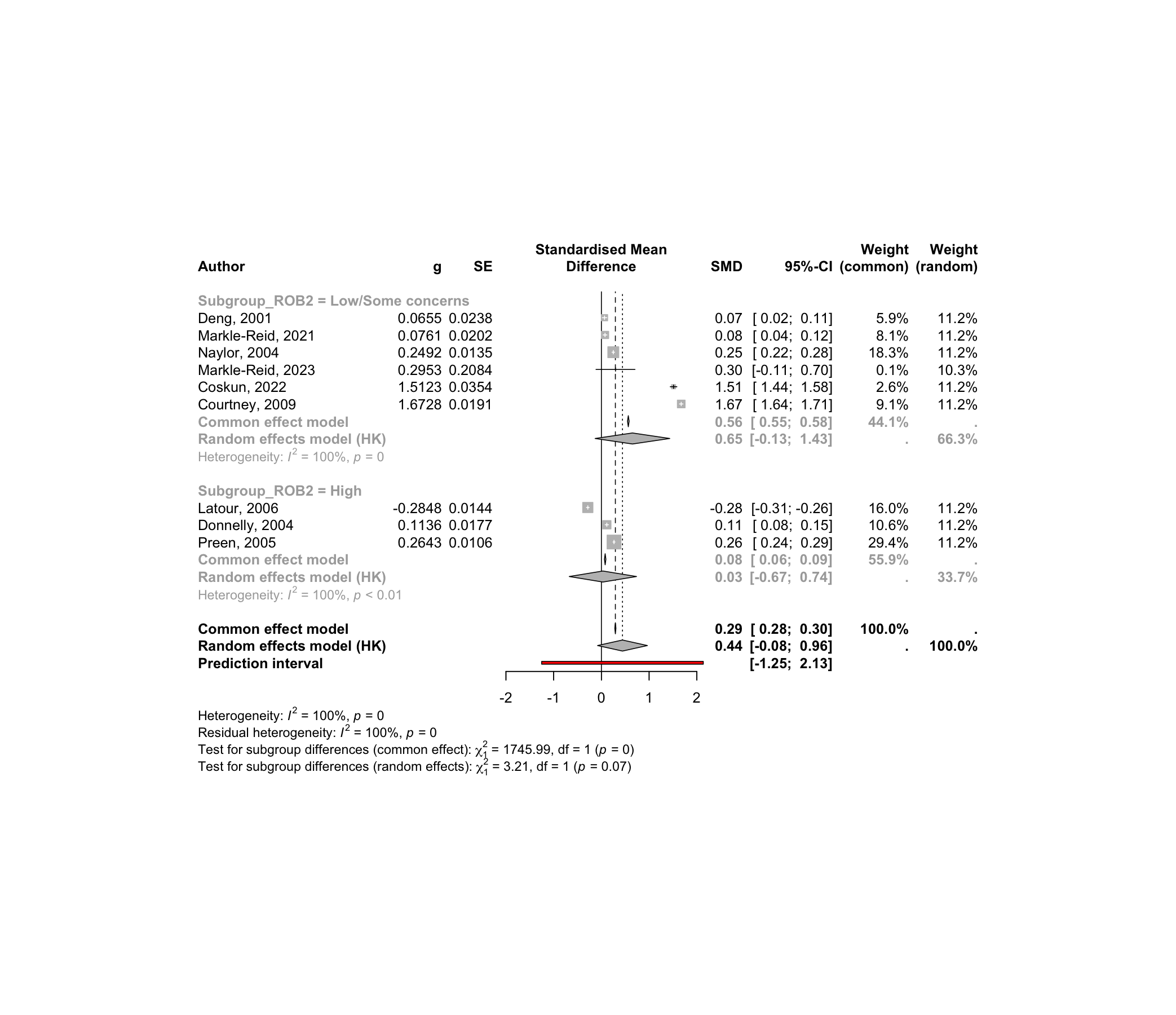


### Subgroup analysis per intervention type


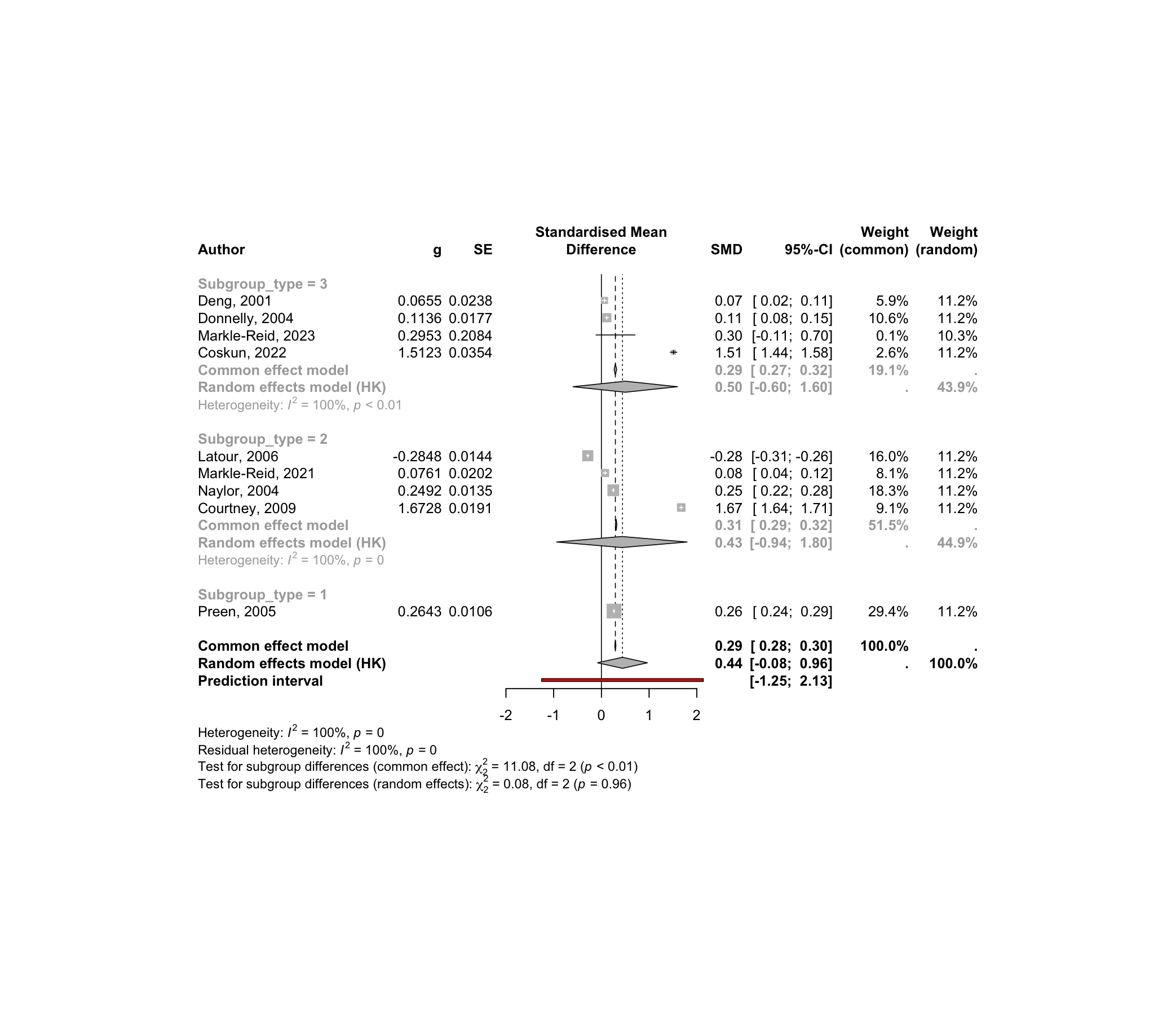


## Depression

### Forest plot overall analysis


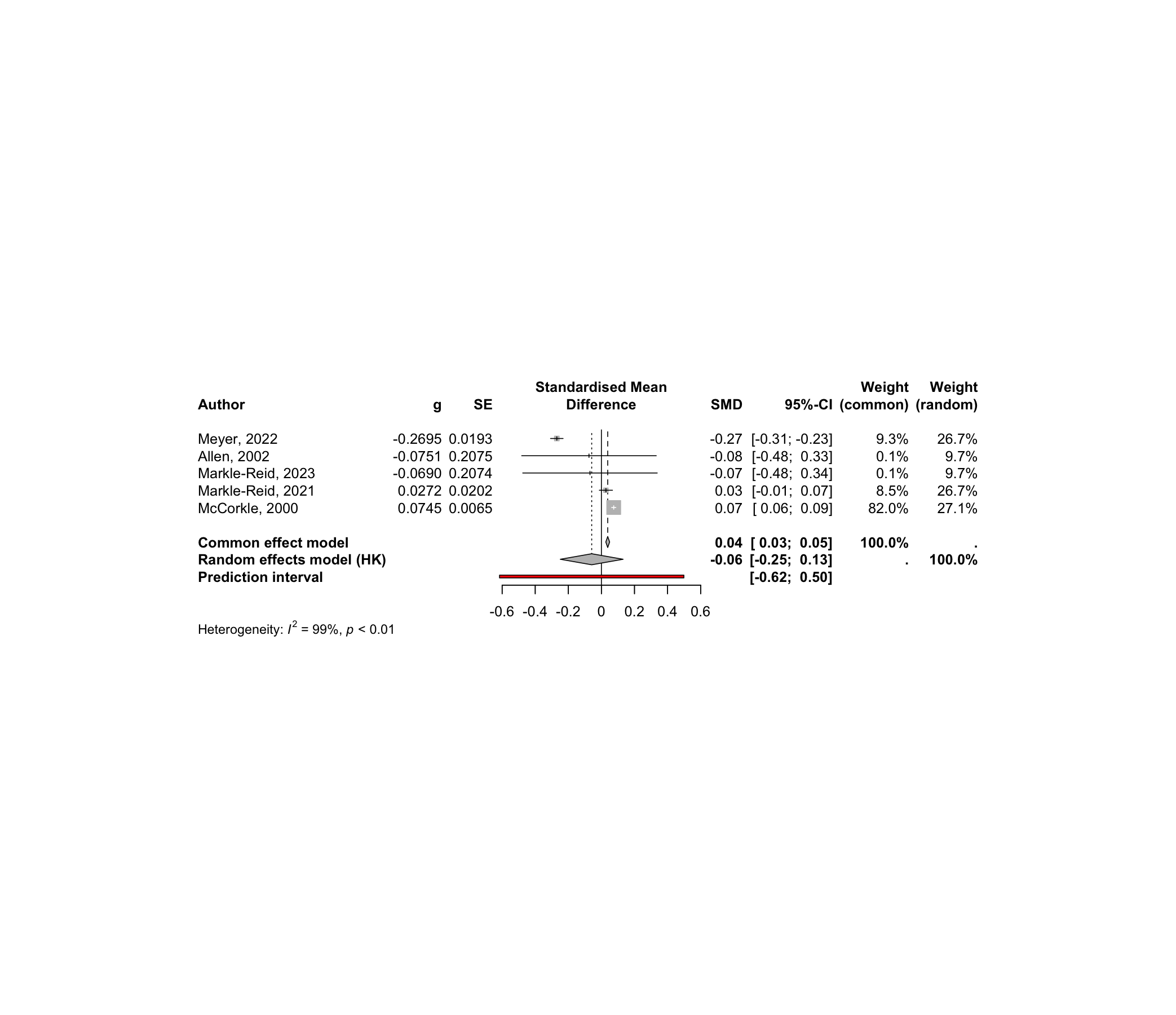


### Funnel plot


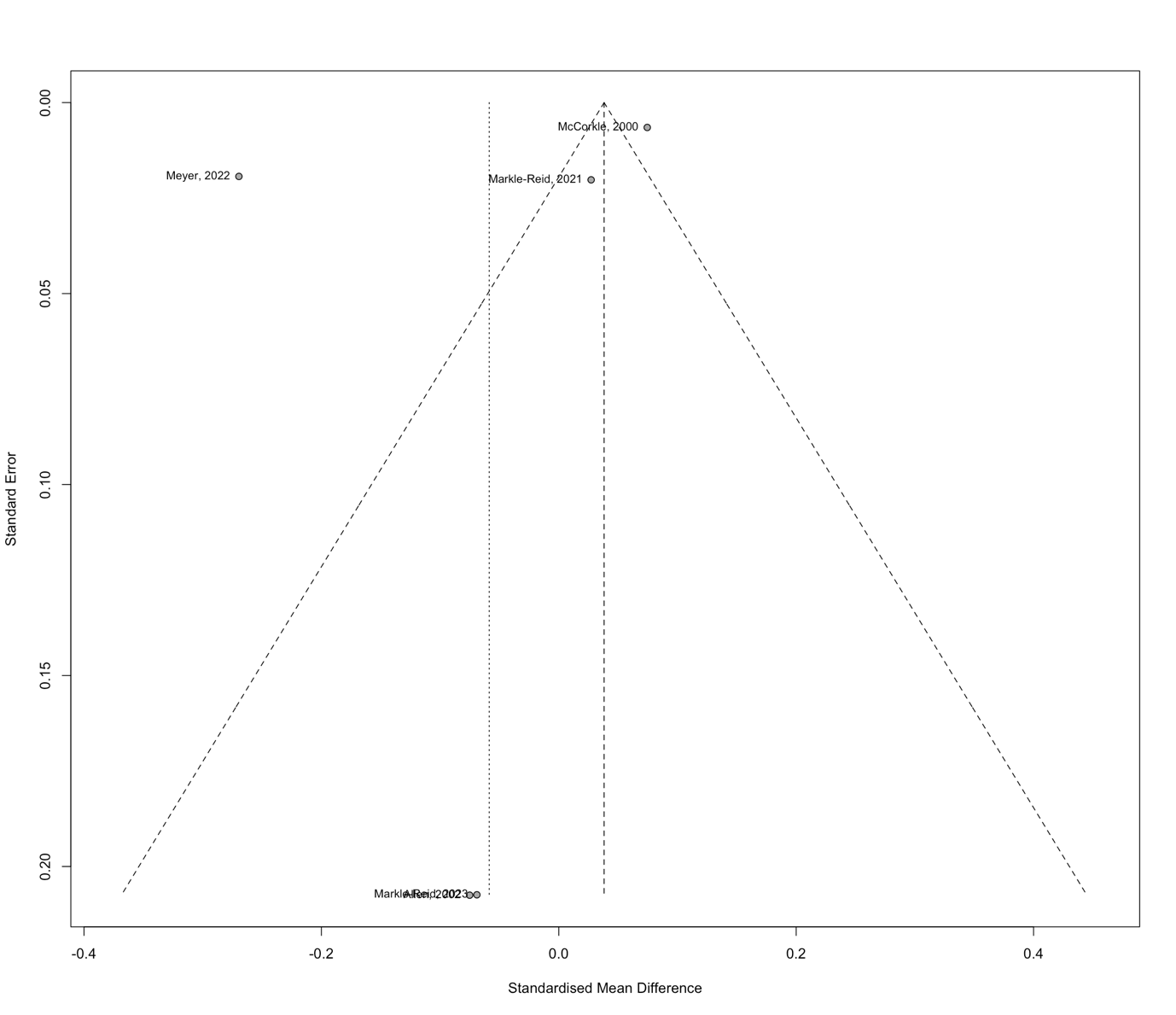


Eggers' test of the intercept

P= 0.457

Eggers' test does not indicate the presence of funnel plot asymmetry.

### Subgroup analysis excluding high-risk-of-bias studies


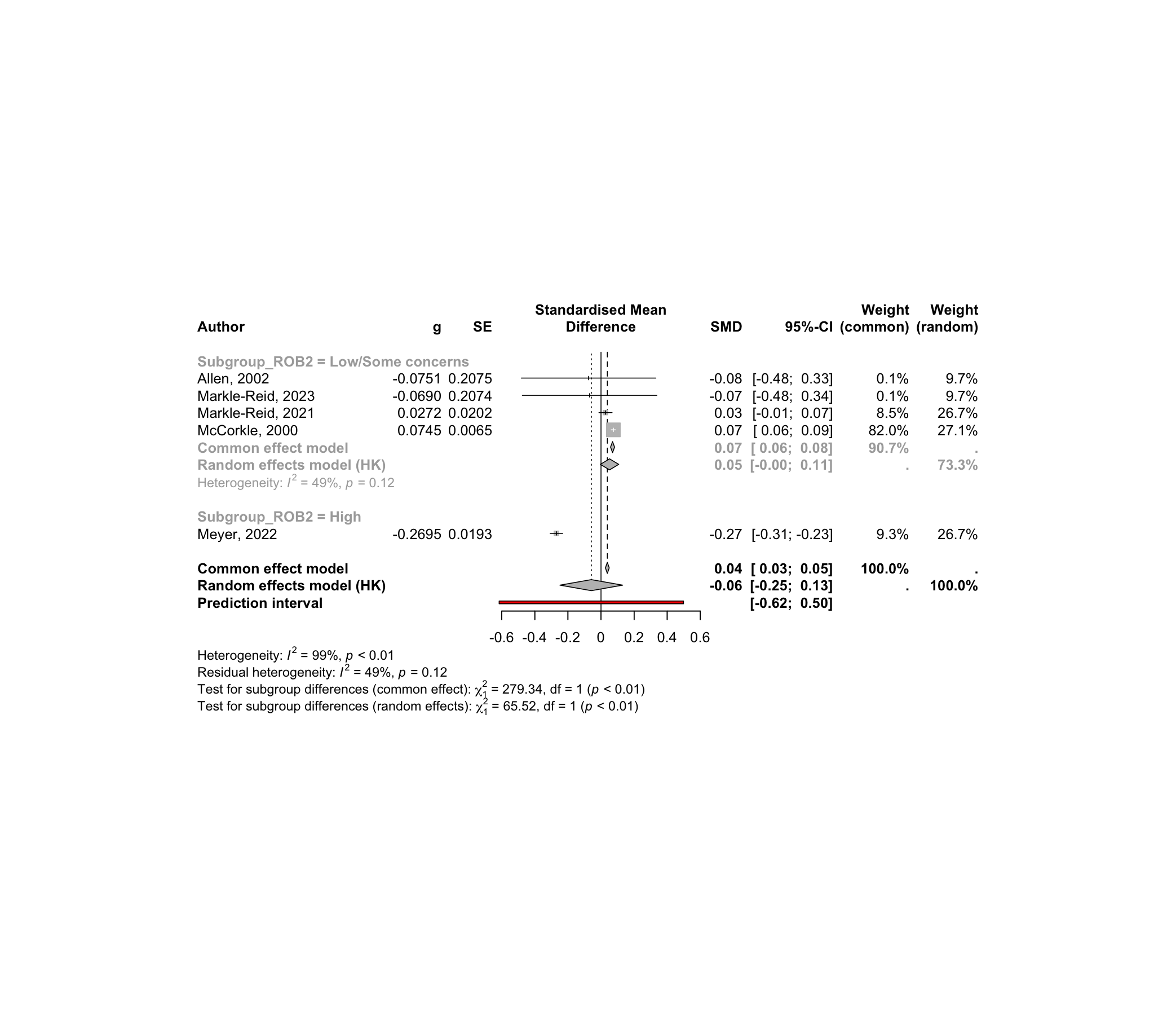


### Subgroup analysis per intervention type

**
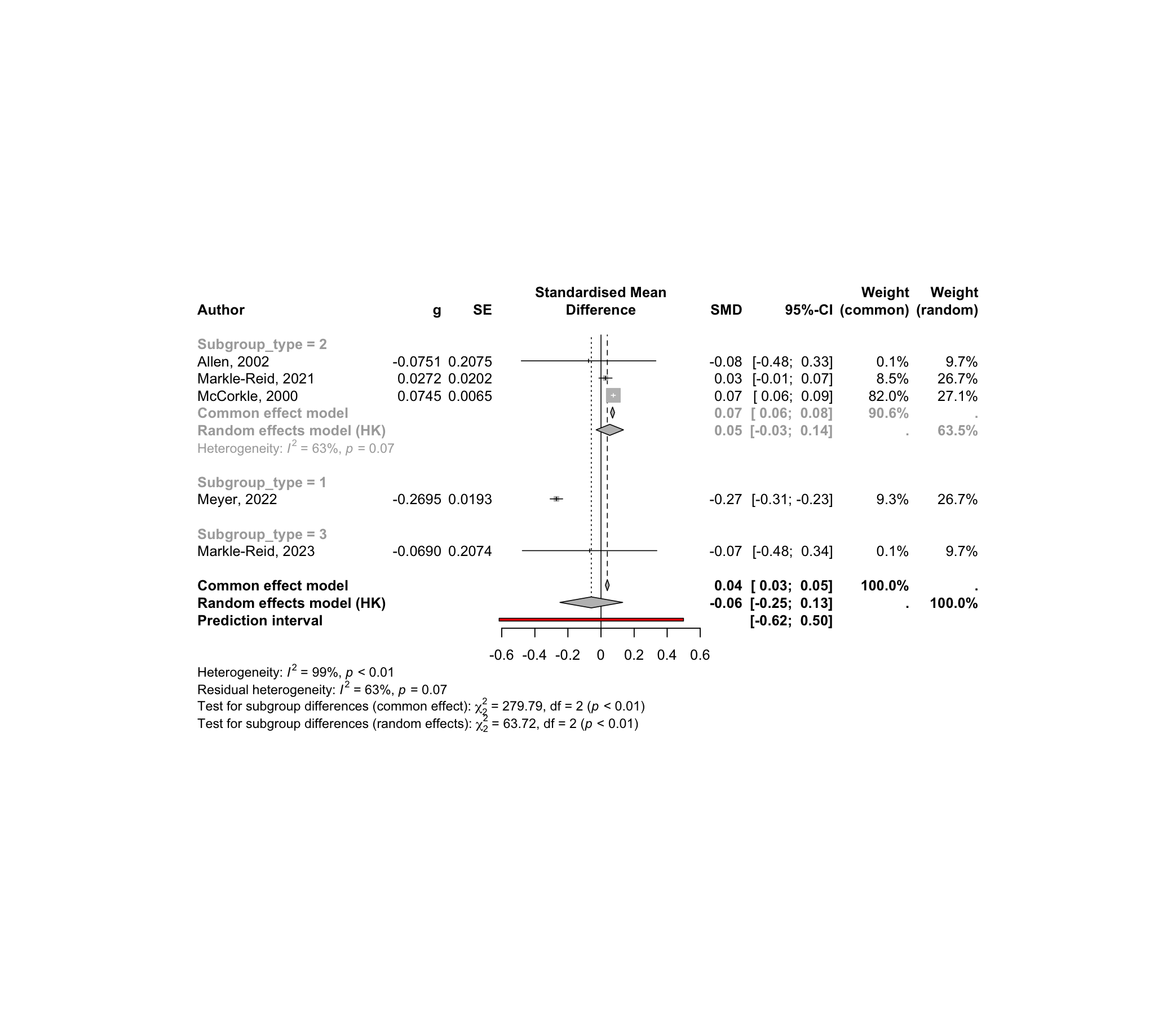
**

## Anxiety

### Forest plot overall analysis


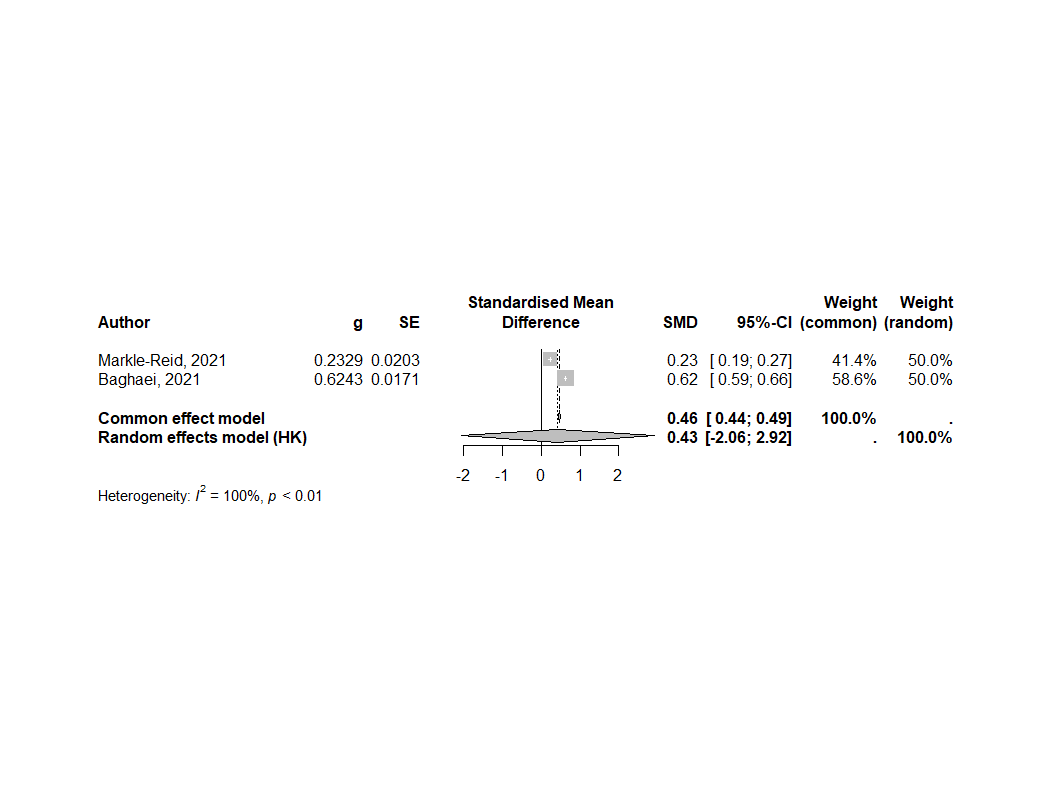


## Physical performance

### Forest plot overall analysis


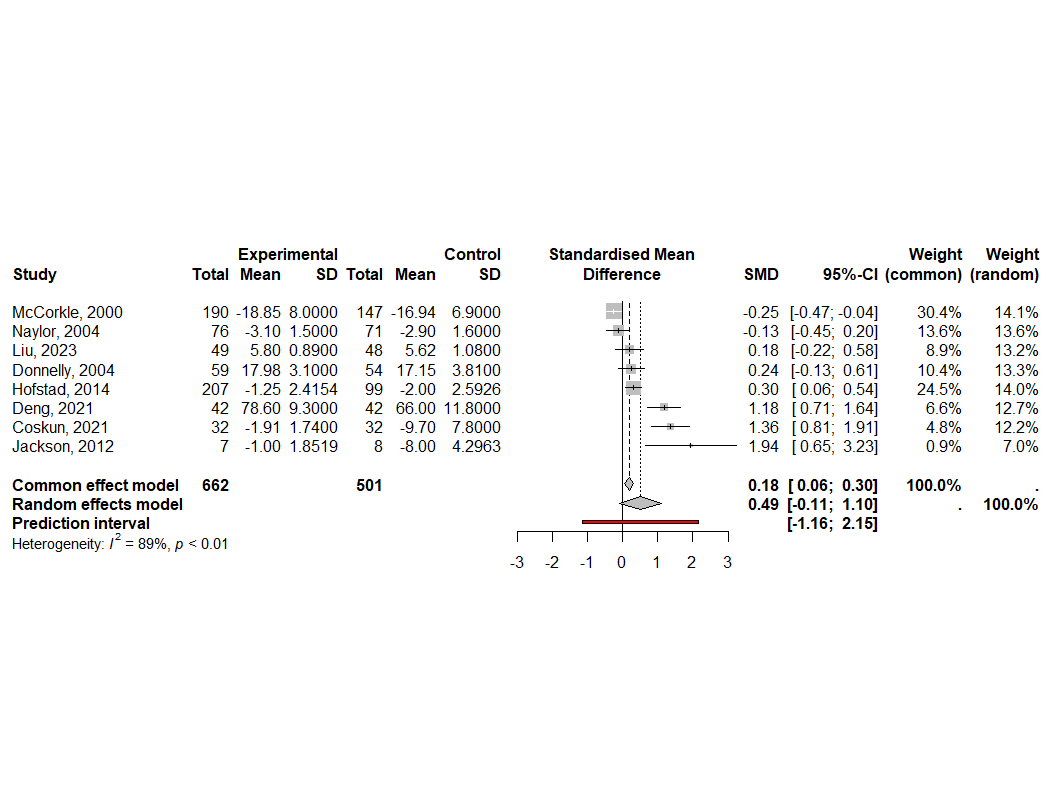


### Funnel plot


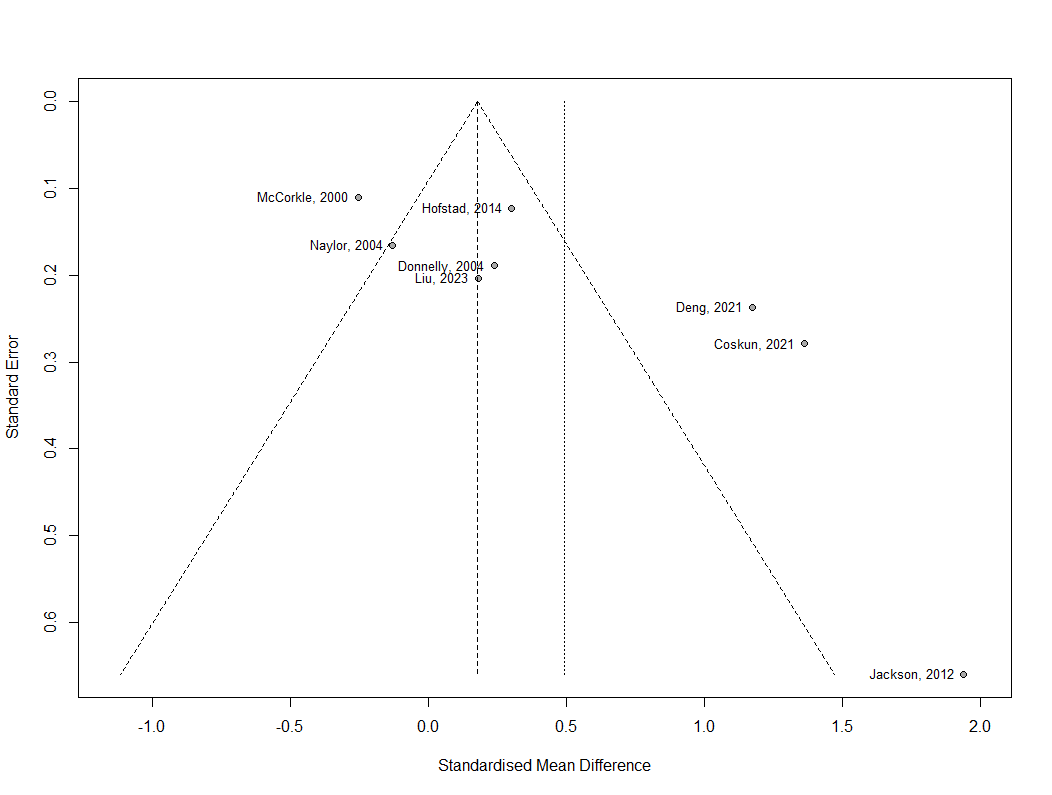


Eggers' test of the intercept

P= 0.029

Eggers' test indicates the presence of funnel plot asymmetry.

### Subgroup analysis excluding high-risk-of-bias studies


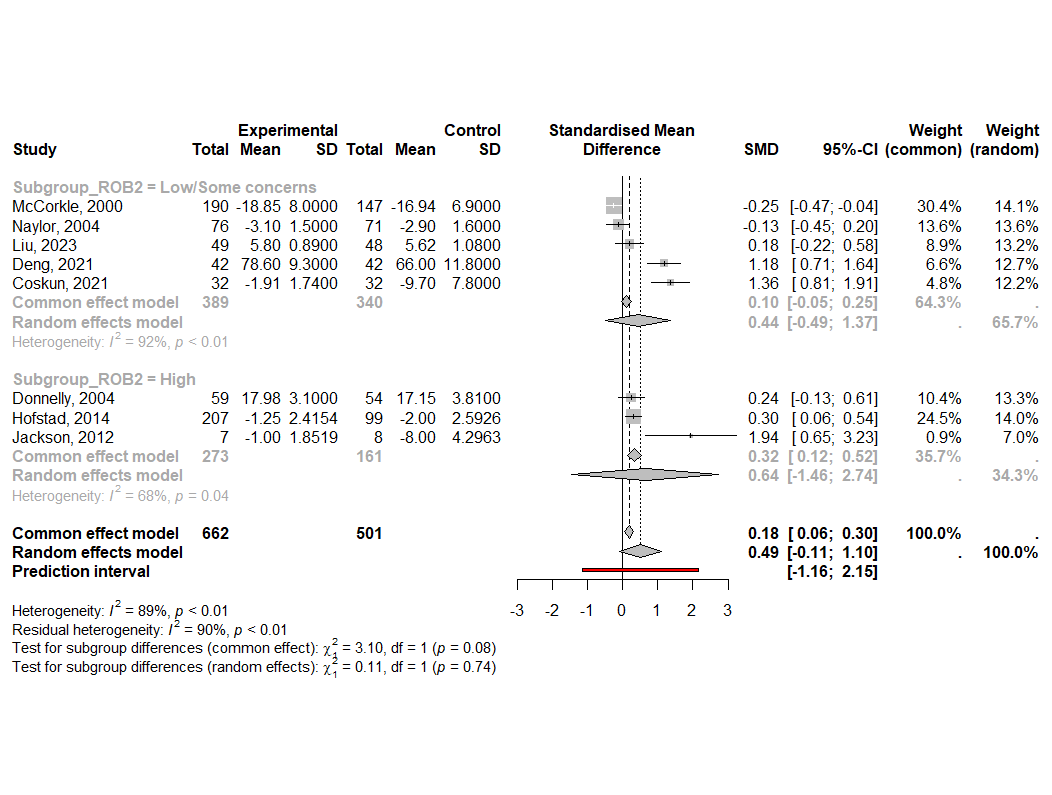


### Subgroup analysis per intervention type


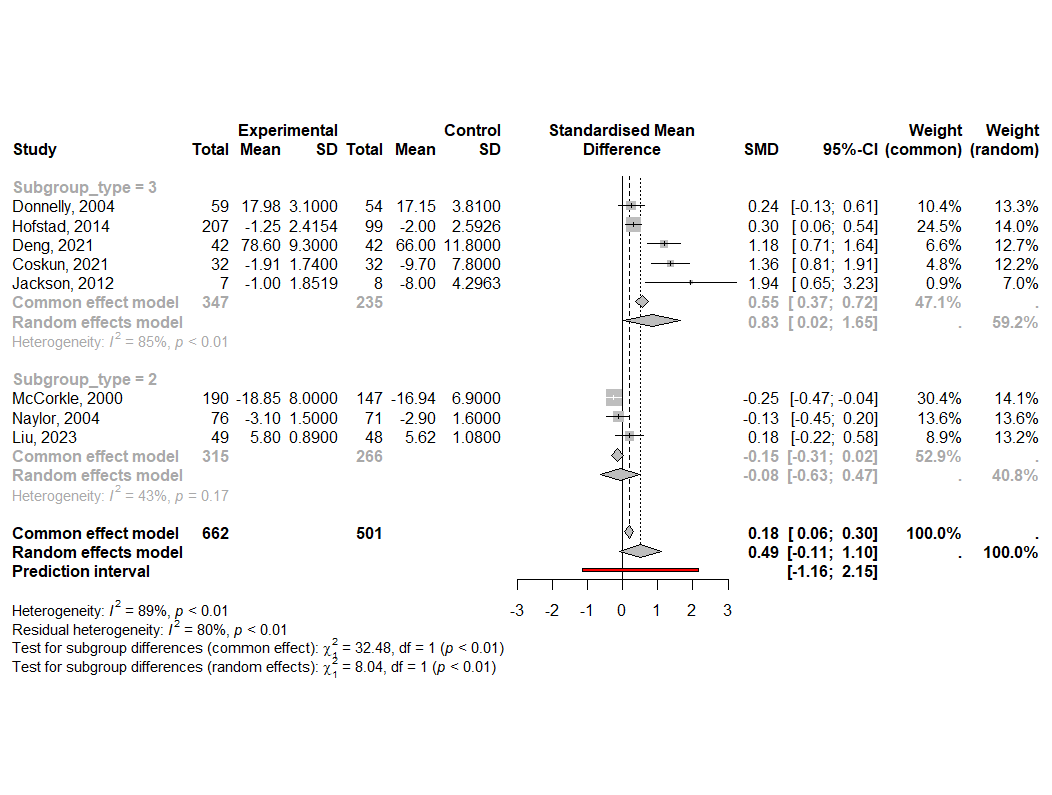


## Physical capacity

### Forest plot overall analysis

**
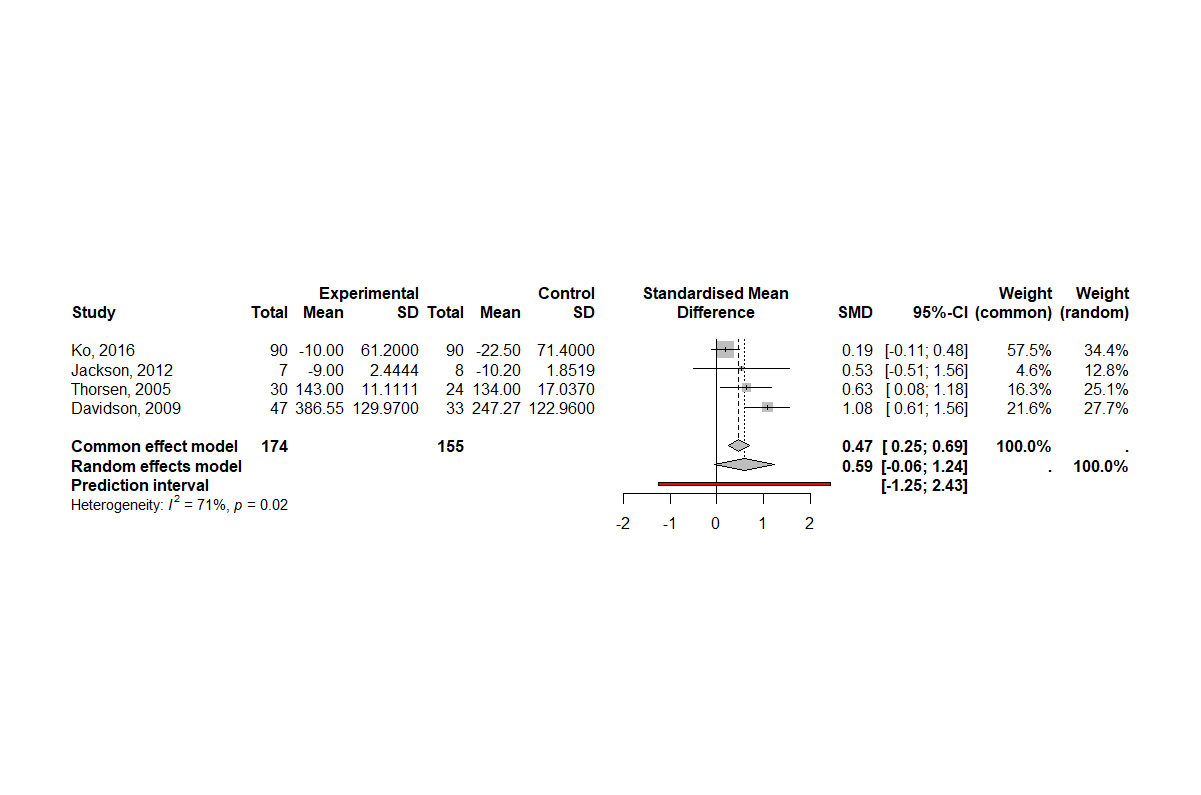
**

### Funnel plot


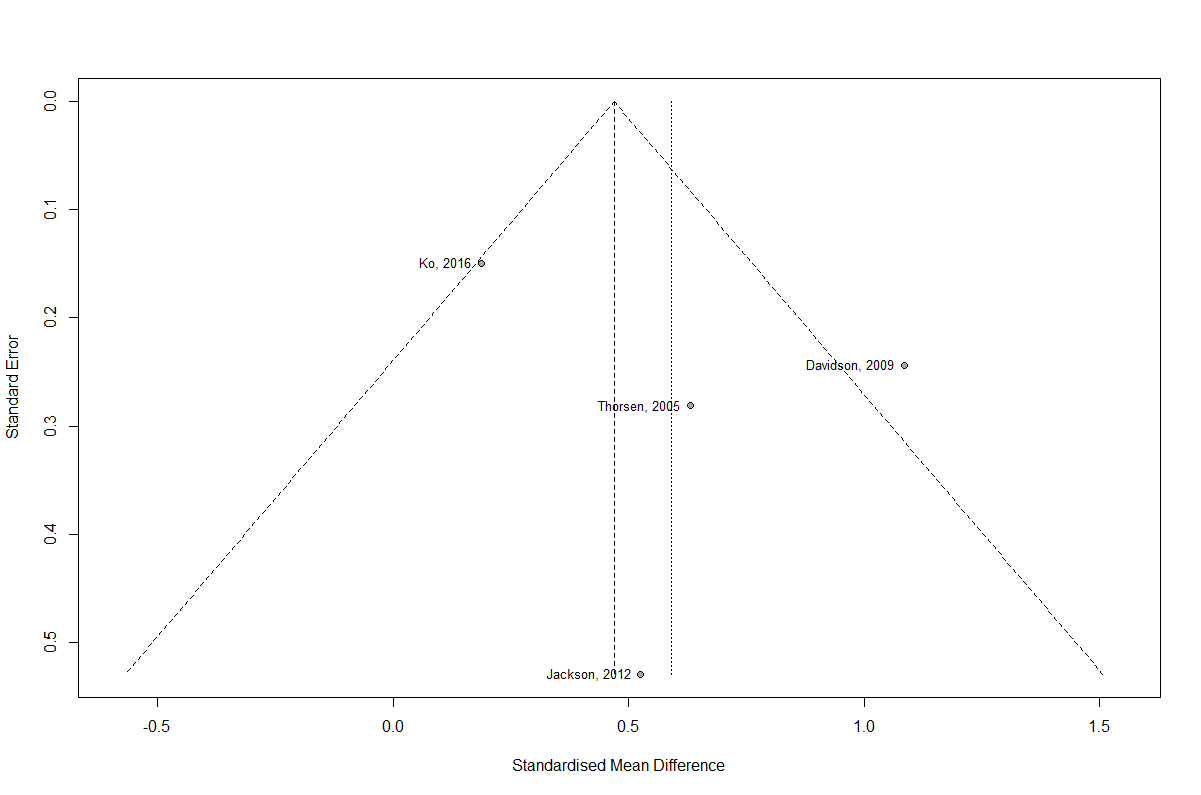


Eggers' test of the intercept

P= 0.472

Eggers' test does not indicate the presence of funnel plot asymmetry.

## Patient satisfaction

### Forest plot overall analysis


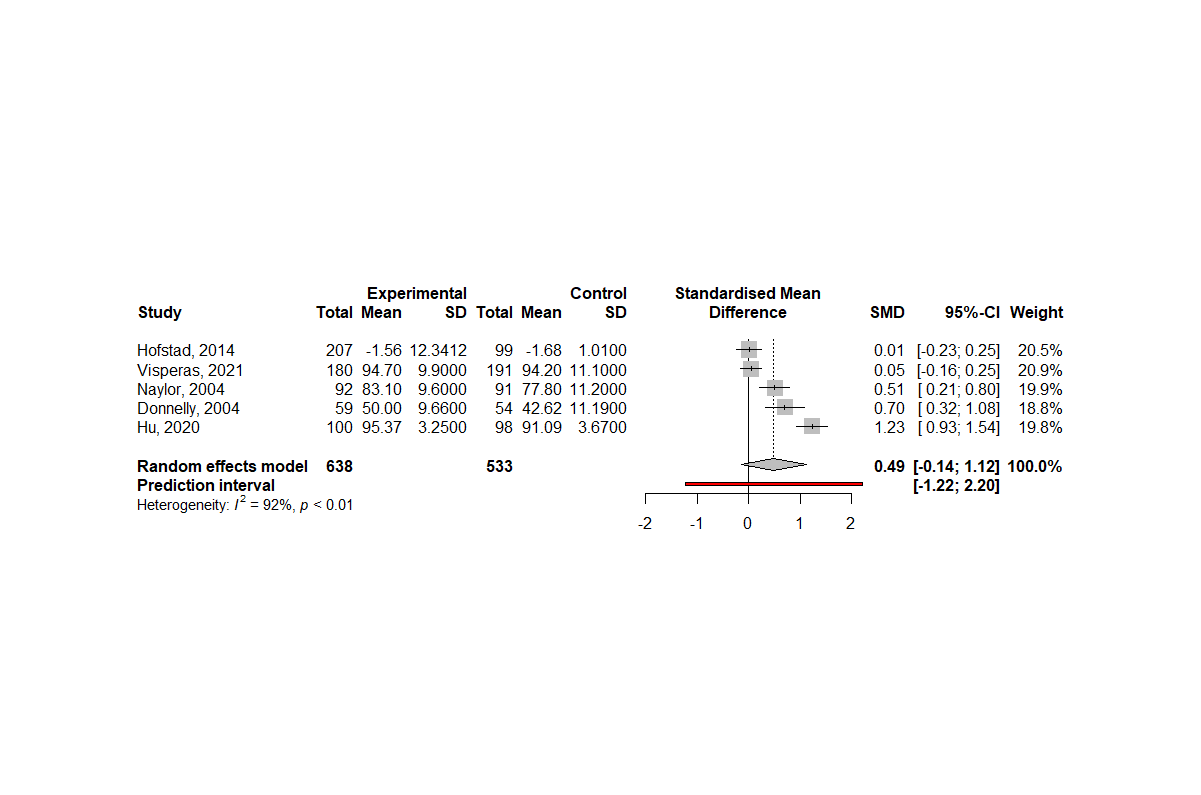


### Funnel plot


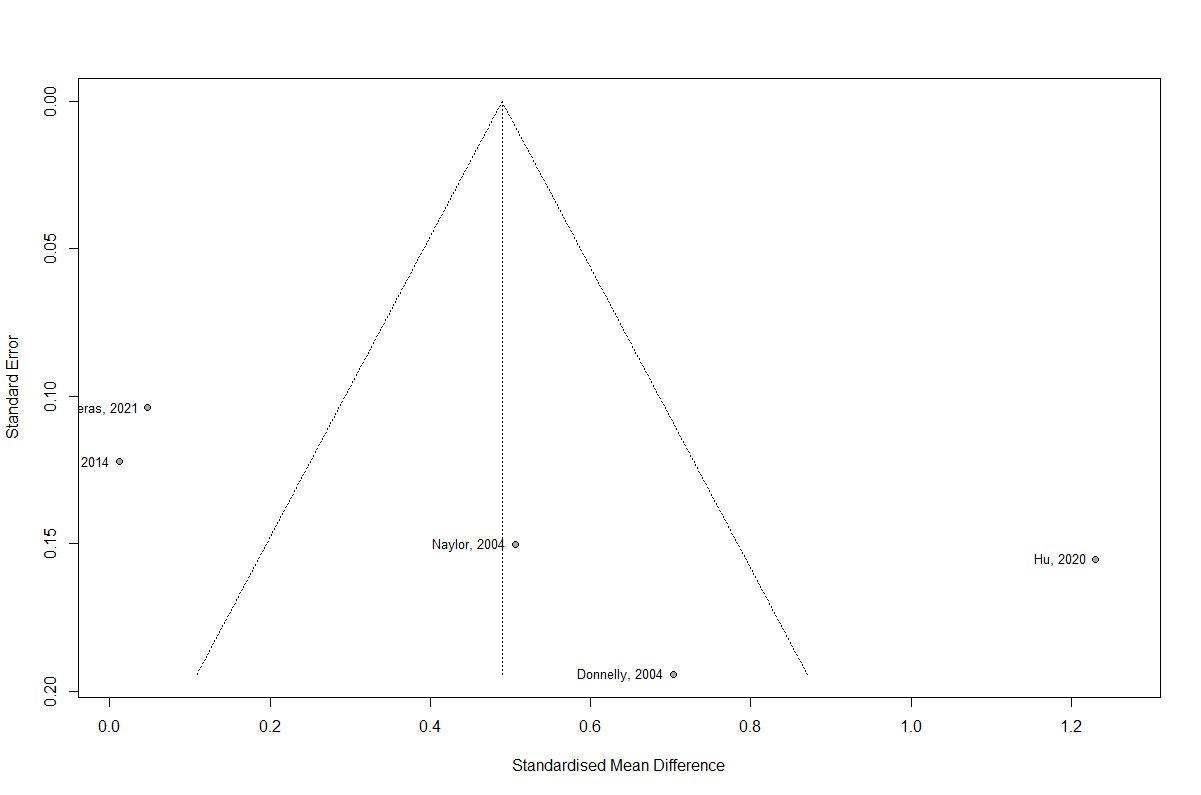


Eggers' test of the intercept

P= 0.140

Eggers' test does not indicate the presence of funnel plot asymmetry.

### Subgroup analysis excluding high-risk-of-bias studies


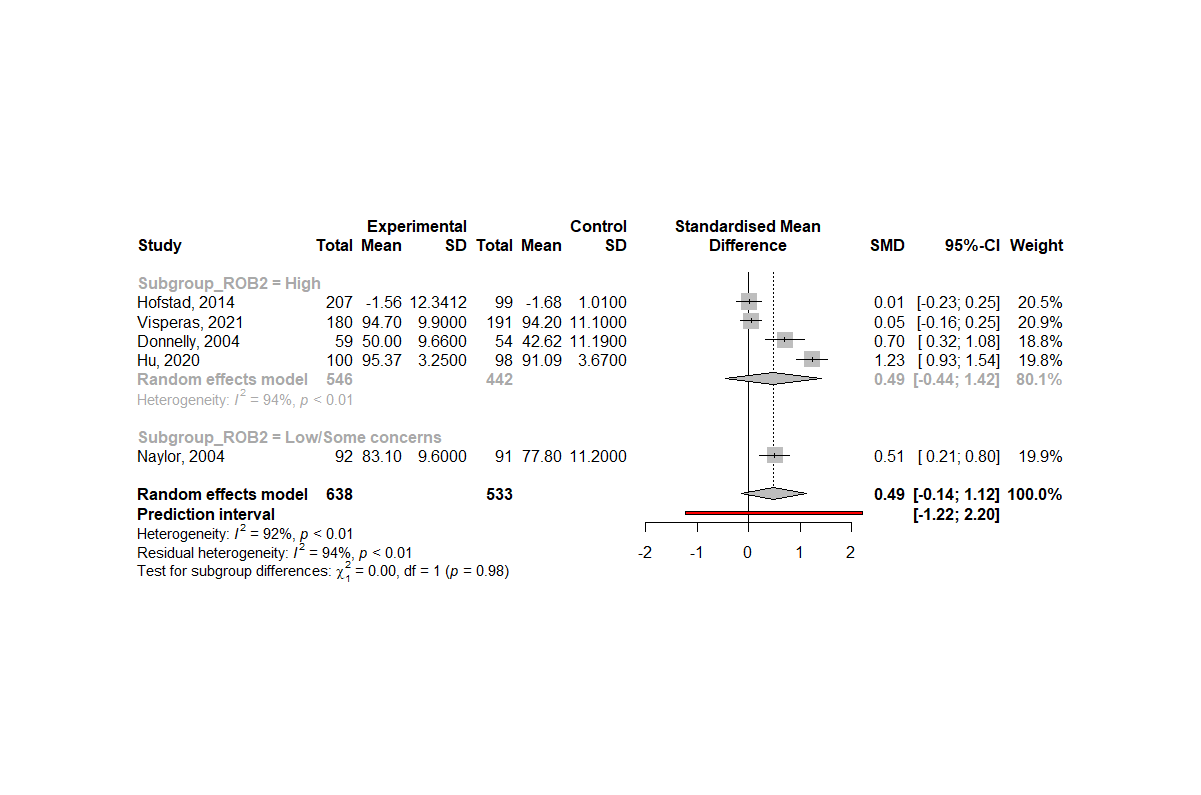


### Subgroup analysis per intervention type


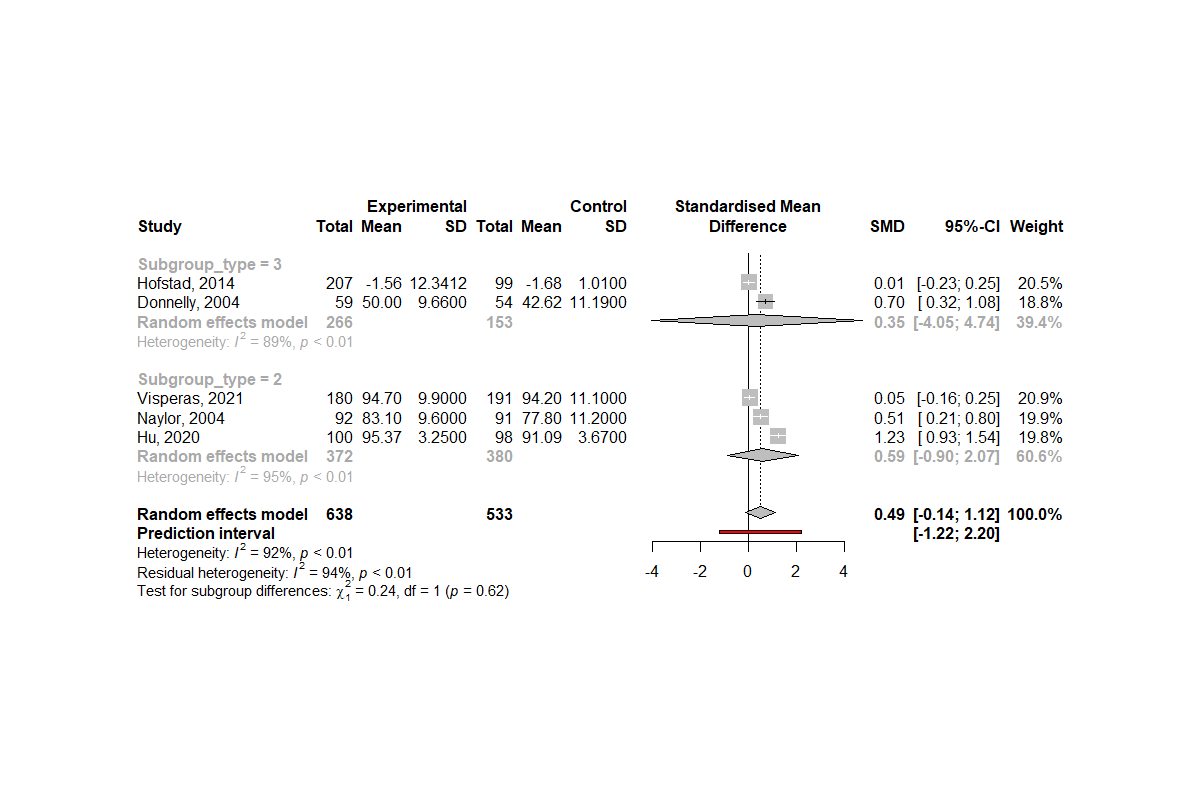


# Appendix 4: Subgroup effects credibility assessment (ICEMAN)

### Readmissions: Subgroup analysis per intervention type

Instrument to assess the Credibility of Effect Modification Analyses (ICEMAN)

in a meta-analysis of randomized controlled trials

*Version 1.1*

| CREDIBILITY ASSESSMENT | | | | |
| --- | --- | --- | --- | --- |
| Essential preliminary considerations to define the possible effect modification of interest | | | |  |
| State a single candidate effect modifier (e.g., age or comorbidity): | | | |  |
| Was the effect modifier measured before or at randomization? [ X ] yes, continue [ ] no, stop here and refer to manual for further instructions | | | |  |
| State a single outcome and time-point (e.g., mortality at 1 year follow-up): | | | |  |
| State a single effect measure (e.g., relative risk or risk difference): | | | |  |
| 1: Is the analysis of effect modification based on comparison within rather than between trials? | | | | |
| [ X ] Completely between | [ ] Mostly between or unclear | [ ] Mostly within | [ ] Completely within | |
| *Subgroup analysis or meta-regression comparing overall effects of each individual trial. This is typical for aggregate data meta-analysis.* | *Subgroup analysis or meta-regression with most information coming from overall effects, but some trials providing within-trial subgroup information* | *Most trials providing within-trial subgroup information; or individual participant data analysis that combines within and between trial information* | *All trials providing within-trial subgroup information or individual participant data; and the analysis separates within from between trial information, e.g., meta-analysis of interactions* | |
| Comment: | | | | |
| 2: For within-trial comparisons, is the effect modification similar from trial to trial? [ X ] Not applicable: no or one within-RCT comparison | | | | |
| [ ] Definitely not similar | [ ] Probably not similar or unclear | [ ] Mostly similar | [ ] Definitely similar | |
| *Effect modification reported for two or more trials and clearly different directions* | *Effect modification not reported for individual trials or too imprecise to tell* | *Effect modification reported for two or more trials, mostly similar in direction, but considerable differences in magnitude* | *Effect modification reported for two or more trials, similar in direction, only some differences in magnitude* | |
| Comment: | | | | |
| 3: For between-trial comparisons, is the number of trials large? [ ] Not applicable: no between RCT comparison | | | | |
| [ ] Very small | [ X ] Rather small or unclear | [ ] Rather large | [ ] Large | |
| *1 or 2 or in smallest subgroup; 5 or less in continuous meta-regression* | *3-4 in smallest subgroup; 6-10 in continuous meta-regression* | *5-9 in smallest subgroup; 11 to 15 in continuous meta-regression* | *10 or more in smallest subgroup; more than 15 in continuous meta-regression* | |
| Comment: | | | | |
| 4: Was the direction of effect modification correctly hypothesized a priori? | | | | |
| [ ] Definitely no | [ ] Probably no or unclear | [ X ] Probably yes | [ ] Definitely yes | |
| *Clearly post-hoc or results inconsistent with hypothesized direction or biologically very implausible* | *Vague hypothesis or hypothesized direction unclear* | *No prior protocol available but unequivocal statement of a priori hypothesis with correct direction of effect modification* | *Prior protocol available and includes correct specification of direction of effect modification, e.g., based on a biologic rationale* | |
| Comment: | | | | |
| 5: Does a test for interaction suggest that chance is an unlikely explanation of the apparent effect modification? (consider irrespective of number of effect modifiers) | | | | |
| [ X ] Chance a very likely explanation | [ ] Chance a likely explanation or unclear | [ ] Chance may not explain | [ ] Chance an unlikely explanation | |
| *Interaction or meta-regression p-value >0.05* | *Interaction or meta-regression p-value ≤0.05 and >0.01, or no test of interaction reported and not computable* | *Interaction or meta-regression p-value ≤0.01 and >0.005* | *Interaction or meta-regression p-value ≤0.005* | |
| Comment: | | | | |
| 6: Did the authors test only a small number of effect modifiers or consider the number in their statistical analysis? | | | | |
| [ ] Definitely no | [ ] Probably no or unclear | [ X ] Probably yes | [ ] Definitely yes | |
| *Explicitly exploratory analysis or large number of effect modifiers tested (e.g., greater than 10) and multiplicity not considered in analysis* | *No mention of number or 4-10 effect modifiers tested and number not considered in analysis* | *No protocol available but unequivocal statement of 3 or fewer effect modifiers tested* | *Protocol available and 3 or fewer effect modifiers tested or number considered in analysis* | |
| Comment: | | | | |
| 7: Did the authors use a random effects model? | | | | |
| [ ] Definitely no | [ ] Probably no or unclear | [ ] Probably yes | [ X ] Definitely yes | |
| *Fixed (or common) effect or fixed effects model explicitly stated* | *Probably fixed effect(s) model* | *Probably random (or mixed) effects* | *Random (or mixed) effects explicitly stated* | |
| Comment: | | | | |
| 8: If the effect modifier is a continuous variable, were arbitrary cut points avoided? [ X ] not applicable: not continuous | | | | |
| [ ] Definitely no | [ ] Probably no or unclear | [ ] Probably yes | [ ] Definitely yes | |
| *Analysis based on exploratory cut point(s), e.g., picking cut point associated with highest interaction p-value* | *Analysis based on cut point(s) of unclear origin* | *Analysis based on pre-specified cut point(s), e.g., suggested by prior RCT* | *Analysis based on the full continuum, e.g., assuming a linear or logarithmic relationship* | |
| Comment: | | | | |
| 9 Optional: Are there any additional considerations that may increase or decrease credibility? (manual section 3.9) [ ] not applicable | | | | |
|  | [ ] Yes, probably decrease | [ ] Yes, probably increase | | |
| Comment:   \| 10: How would you rate the overall credibility of the proposed effect modification?  The overall rating should be driven by the items that decrease credibility. The following provides a sensible strategy:  All responses definitely or probably decrease credibility or unclear 🡪 very low  Two or more responses definitely decrease credibility 🡪 maximum usually low even if all other responses satisfy credibility criteria  One response definitely decreases credibility 🡪 maximum usually moderate even if all other responses satisfy credibility criteria  Two responses probably decrease credibility 🡪 maximum usually moderate even if all other responses satisfy credibility criteria  No response options definitely or probably decrease credibility 🡪 high very likely  Place a mark on the continuous line (or type “x” in editable version) \| \| \| \| \|  \| \| --- \| --- \| --- \| --- \| --- \| --- \| \|  \|  \| \| \| \|  \| \|  \| X \| \| \| \|  \| \|  \|  \| \|  \|  \| \| \| \|  \| \|  \|  \| \| \| \|  \| \|  \| Very low credibility \| Low credibility \| Moderate credibility \| High credibility \|  \| \|  \|  \|  \|  \|  \|  \| \|  \| Minimal to no support for effect modification;  Use overall effect for each subgroup \| Some but insufficient support for effect modification;  Use overall effect for each subgroup but note remaining uncertainty \| Likely effect modification;  Use separate effects for each subgroup but note remaining uncertainty \| Very likely effect modification;  Use separate effects for each subgroup \|  \| \|  \| \| \| \| \| \| | | | | |

### Physical quality of life: Subgroup analysis per risk of bias

CREDIBILITY ASSESSMENT

| Essential preliminary considerations to define the possible effect modification of interest | | | |  |
| --- | --- | --- | --- | --- |
| State a single candidate effect modifier (e.g., age or comorbidity): | | | |  |
| Was the effect modifier measured before or at randomization? [ X ] yes, continue [ ] no, stop here and refer to manual for further instructions | | | |  |
| State a single outcome and time-point (e.g., mortality at 1 year follow-up): | | | |  |
| State a single effect measure (e.g., relative risk or risk difference): | | | |  |
| 1: Is the analysis of effect modification based on comparison within rather than between trials? | | | | |
| [ X ] Completely between | [ ] Mostly between or unclear | [ ] Mostly within | [ ] Completely within | |
| *Subgroup analysis or meta-regression comparing overall effects of each individual trial. This is typical for aggregate data meta-analysis.* | *Subgroup analysis or meta-regression with most information coming from overall effects, but some trials providing within-trial subgroup information* | *Most trials providing within-trial subgroup information; or individual participant data analysis that combines within and between trial information* | *All trials providing within-trial subgroup information or individual participant data; and the analysis separates within from between trial information, e.g., meta-analysis of interactions* | |
| Comment: | | | | |
| 2: For within-trial comparisons, is the effect modification similar from trial to trial? [ X ] Not applicable: no or one within-RCT comparison | | | | |
| [ ] Definitely not similar | [ ] Probably not similar or unclear | [ ] Mostly similar | [ ] Definitely similar | |
| *Effect modification reported for two or more trials and clearly different directions* | *Effect modification not reported for individual trials or too imprecise to tell* | *Effect modification reported for two or more trials, mostly similar in direction, but considerable differences in magnitude* | *Effect modification reported for two or more trials, similar in direction, only some differences in magnitude* | |
| Comment: | | | | |
| 3: For between-trial comparisons, is the number of trials large? [ ] Not applicable: no between RCT comparison | | | | |
| [ X ] Very small | [ ] Rather small or unclear | [ ] Rather large | [ ] Large | |
| *1 or 2 or in smallest subgroup; 5 or less in continuous meta-regression* | *3-4 in smallest subgroup; 6-10 in continuous meta-regression* | *5-9 in smallest subgroup; 11 to 15 in continuous meta-regression* | *10 or more in smallest subgroup; more than 15 in continuous meta-regression* | |
| Comment: | | | | |
| 4: Was the direction of effect modification correctly hypothesized a priori? | | | | |
| [ ] Definitely no | [ ] Probably no or unclear | [ X ] Probably yes | [ ] Definitely yes | |
| *Clearly post-hoc or results inconsistent with hypothesized direction or biologically very implausible* | *Vague hypothesis or hypothesized direction unclear* | *No prior protocol available but unequivocal statement of a priori hypothesis with correct direction of effect modification* | *Prior protocol available and includes correct specification of direction of effect modification, e.g., based on a biologic rationale* | |
| Comment: | | | | |
| 5: Does a test for interaction suggest that chance is an unlikely explanation of the apparent effect modification? (consider irrespective of number of effect modifiers) | | | | |
| [ ] Chance a very likely explanation | [ ] Chance a likely explanation or unclear | [ X ] Chance may not explain | [ ] Chance an unlikely explanation | |
| *Interaction or meta-regression p-value >0.05* | *Interaction or meta-regression p-value ≤0.05 and >0.01, or no test of interaction reported and not computable* | *Interaction or meta-regression p-value ≤0.01 and >0.005* | *Interaction or meta-regression p-value ≤0.005* | |
| Comment: | | | | |
| 6: Did the authors test only a small number of effect modifiers or consider the number in their statistical analysis? | | | | |
| [ ] Definitely no | [ ] Probably no or unclear | [ X ] Probably yes | [ ] Definitely yes | |
| *Explicitly exploratory analysis or large number of effect modifiers tested (e.g., greater than 10) and multiplicity not considered in analysis* | *No mention of number or 4-10 effect modifiers tested and number not considered in analysis* | *No protocol available but unequivocal statement of 3 or fewer effect modifiers tested* | *Protocol available and 3 or fewer effect modifiers tested or number considered in analysis* | |
| Comment: | | | | |
| 7: Did the authors use a random effects model? | | | | |
| [ ] Definitely no | [ ] Probably no or unclear | [ ] Probably yes | [ X ] Definitely yes | |
| *Fixed (or common) effect or fixed effects model explicitly stated* | *Probably fixed effect(s) model* | *Probably random (or mixed) effects* | *Random (or mixed) effects explicitly stated* | |
| Comment: | | | | |
| 8: If the effect modifier is a continuous variable, were arbitrary cut points avoided? [ X ] not applicable: not continuous | | | | |
| [ ] Definitely no | [ ] Probably no or unclear | [ ] Probably yes | [ ] Definitely yes | |
| *Analysis based on exploratory cut point(s), e.g., picking cut point associated with highest interaction p-value* | *Analysis based on cut point(s) of unclear origin* | *Analysis based on pre-specified cut point(s), e.g., suggested by prior RCT* | *Analysis based on the full continuum, e.g., assuming a linear or logarithmic relationship* | |
| Comment: | | | | |
| 9 Optional: Are there any additional considerations that may increase or decrease credibility? (manual section 3.9) [ ] not applicable | | | | |
|  | [ ] Yes, probably decrease | [ ] Yes, probably increase | | |
| Comment:   \| 10: How would you rate the overall credibility of the proposed effect modification?  The overall rating should be driven by the items that decrease credibility. The following provides a sensible strategy:  All responses definitely or probably decrease credibility or unclear 🡪 very low  Two or more responses definitely decrease credibility 🡪 maximum usually low even if all other responses satisfy credibility criteria  One response definitely decreases credibility 🡪 maximum usually moderate even if all other responses satisfy credibility criteria  Two responses probably decrease credibility 🡪 maximum usually moderate even if all other responses satisfy credibility criteria  No response options definitely or probably decrease credibility 🡪 high very likely  Place a mark on the continuous line (or type “x” in editable version) \| \| \| \| \|  \| \| --- \| --- \| --- \| --- \| --- \| --- \| \|  \|  \| \| \| \|  \| \|  \| X \| \| \| \|  \| \|  \|  \| \|  \|  \| \| \| \|  \| \|  \|  \| \| \| \|  \| \|  \| Very low credibility \| Low credibility \| Moderate credibility \| High credibility \|  \| \|  \|  \|  \|  \|  \|  \| \|  \| Minimal to no support for effect modification;  Use overall effect for each subgroup \| Some but insufficient support for effect modification;  Use overall effect for each subgroup but note remaining uncertainty \| Likely effect modification;  Use separate effects for each subgroup but note remaining uncertainty \| Very likely effect modification;  Use separate effects for each subgroup \|  \| \|  \| \| \| \| \| \| | | | | |

### Depression: Subgroup analysis per risk of bias and per intervention type

| CREDIBILITY ASSESSMENT | | | | |
| --- | --- | --- | --- | --- |
| Essential preliminary considerations to define the possible effect modification of interest | | | |  |
| State a single candidate effect modifier (e.g., age or comorbidity): | | | |  |
| Was the effect modifier measured before or at randomization? [ X ] yes, continue [ ] no, stop here and refer to manual for further instructions | | | |  |
| State a single outcome and time-point (e.g., mortality at 1 year follow-up): | | | |  |
| State a single effect measure (e.g., relative risk or risk difference): | | | |  |
| 1: Is the analysis of effect modification based on comparison within rather than between trials? | | | | |
| [ X ] Completely between | [ ] Mostly between or unclear | [ ] Mostly within | [ ] Completely within | |
| *Subgroup analysis or meta-regression comparing overall effects of each individual trial. This is typical for aggregate data meta-analysis.* | *Subgroup analysis or meta-regression with most information coming from overall effects, but some trials providing within-trial subgroup information* | *Most trials providing within-trial subgroup information; or individual participant data analysis that combines within and between trial information* | *All trials providing within-trial subgroup information or individual participant data; and the analysis separates within from between trial information, e.g., meta-analysis of interactions* | |
| Comment: | | | | |
| 2: For within-trial comparisons, is the effect modification similar from trial to trial? [ X ] Not applicable: no or one within-RCT comparison | | | | |
| [ ] Definitely not similar | [ ] Probably not similar or unclear | [ ] Mostly similar | [ ] Definitely similar | |
| *Effect modification reported for two or more trials and clearly different directions* | *Effect modification not reported for individual trials or too imprecise to tell* | *Effect modification reported for two or more trials, mostly similar in direction, but considerable differences in magnitude* | *Effect modification reported for two or more trials, similar in direction, only some differences in magnitude* | |
| Comment: | | | | |
| 3: For between-trial comparisons, is the number of trials large? [ ] Not applicable: no between RCT comparison | | | | |
| [ X ] Very small | [ ] Rather small or unclear | [ ] Rather large | [ ] Large | |
| *1 or 2 or in smallest subgroup; 5 or less in continuous meta-regression* | *3-4 in smallest subgroup; 6-10 in continuous meta-regression* | *5-9 in smallest subgroup; 11 to 15 in continuous meta-regression* | *10 or more in smallest subgroup; more than 15 in continuous meta-regression* | |
| Comment: | | | | |
| 4: Was the direction of effect modification correctly hypothesized a priori? | | | | |
| [ ] Definitely no | [ ] Probably no or unclear | [ X ] Probably yes | [ ] Definitely yes | |
| *Clearly post-hoc or results inconsistent with hypothesized direction or biologically very implausible* | *Vague hypothesis or hypothesized direction unclear* | *No prior protocol available but unequivocal statement of a priori hypothesis with correct direction of effect modification* | *Prior protocol available and includes correct specification of direction of effect modification, e.g., based on a biologic rationale* | |
| Comment: | | | | |
| 5: Does a test for interaction suggest that chance is an unlikely explanation of the apparent effect modification? (consider irrespective of number of effect modifiers) | | | | |
| [ ] Chance a very likely explanation | [ ] Chance a likely explanation or unclear | [ ] Chance may not explain | [ X ] Chance an unlikely explanation | |
| *Interaction or meta-regression p-value >0.05* | *Interaction or meta-regression p-value ≤0.05 and >0.01, or no test of interaction reported and not computable* | *Interaction or meta-regression p-value ≤0.01 and >0.005* | *Interaction or meta-regression p-value ≤0.005* | |
| Comment: | | | | |
| 6: Did the authors test only a small number of effect modifiers or consider the number in their statistical analysis? | | | | |
| [ ] Definitely no | [ ] Probably no or unclear | [ X ] Probably yes | [ ] Definitely yes | |
| *Explicitly exploratory analysis or large number of effect modifiers tested (e.g., greater than 10) and multiplicity not considered in analysis* | *No mention of number or 4-10 effect modifiers tested and number not considered in analysis* | *No protocol available but unequivocal statement of 3 or fewer effect modifiers tested* | *Protocol available and 3 or fewer effect modifiers tested or number considered in analysis* | |
| Comment: | | | | |
| 7: Did the authors use a random effects model? | | | | |
| [ ] Definitely no | [ ] Probably no or unclear | [ ] Probably yes | [ X ] Definitely yes | |
| *Fixed (or common) effect or fixed effects model explicitly stated* | *Probably fixed effect(s) model* | *Probably random (or mixed) effects* | *Random (or mixed) effects explicitly stated* | |
| Comment: | | | | |
| 8: If the effect modifier is a continuous variable, were arbitrary cut points avoided? [ X ] not applicable: not continuous | | | | |
| [ ] Definitely no | [ ] Probably no or unclear | [ ] Probably yes | [ ] Definitely yes | |
| *Analysis based on exploratory cut point(s), e.g., picking cut point associated with highest interaction p-value* | *Analysis based on cut point(s) of unclear origin* | *Analysis based on pre-specified cut point(s), e.g., suggested by prior RCT* | *Analysis based on the full continuum, e.g., assuming a linear or logarithmic relationship* | |
| Comment: | | | | |
| 9 Optional: Are there any additional considerations that may increase or decrease credibility? (manual section 3.9) [ ] not applicable | | | | |
|  | [ ] Yes, probably decrease | [ ] Yes, probably increase | | |
| Comment:   \| 10: How would you rate the overall credibility of the proposed effect modification?  The overall rating should be driven by the items that decrease credibility. The following provides a sensible strategy:  All responses definitely or probably decrease credibility or unclear 🡪 very low  Two or more responses definitely decrease credibility 🡪 maximum usually low even if all other responses satisfy credibility criteria  One response definitely decreases credibility 🡪 maximum usually moderate even if all other responses satisfy credibility criteria  Two responses probably decrease credibility 🡪 maximum usually moderate even if all other responses satisfy credibility criteria  No response options definitely or probably decrease credibility 🡪 high very likely  Place a mark on the continuous line (or type “x” in editable version) \| \| \| \| \|  \| \| --- \| --- \| --- \| --- \| --- \| --- \| \|  \|  \| \| \| \|  \| \|  \| X \| \| \| \|  \| \|  \|  \| \|  \|  \| \| \| \|  \| \|  \|  \| \| \| \|  \| \|  \| Very low credibility \| Low credibility \| Moderate credibility \| High credibility \|  \| \|  \|  \|  \|  \|  \|  \| \|  \| Minimal to no support for effect modification;  Use overall effect for each subgroup \| Some but insufficient support for effect modification;  Use overall effect for each subgroup but note remaining uncertainty \| Likely effect modification;  Use separate effects for each subgroup but note remaining uncertainty \| Very likely effect modification;  Use separate effects for each subgroup \|  \| \|  \| \| \| \| \| \| | | | | |

### Physical performance: Subgroup analysis per intervention type

| CREDIBILITY ASSESSMENT | | | | |
| --- | --- | --- | --- | --- |
| Essential preliminary considerations to define the possible effect modification of interest | | | |  |
| State a single candidate effect modifier (e.g., age or comorbidity): | | | |  |
| Was the effect modifier measured before or at randomization? [ X ] yes, continue [ ] no, stop here and refer to manual for further instructions | | | |  |
| State a single outcome and time-point (e.g., mortality at 1 year follow-up): | | | |  |
| State a single effect measure (e.g., relative risk or risk difference): | | | |  |
| 1: Is the analysis of effect modification based on comparison within rather than between trials? | | | | |
| [ X ] Completely between | [ ] Mostly between or unclear | [ ] Mostly within | [ ] Completely within | |
| *Subgroup analysis or meta-regression comparing overall effects of each individual trial. This is typical for aggregate data meta-analysis.* | *Subgroup analysis or meta-regression with most information coming from overall effects, but some trials providing within-trial subgroup information* | *Most trials providing within-trial subgroup information; or individual participant data analysis that combines within and between trial information* | *All trials providing within-trial subgroup information or individual participant data; and the analysis separates within from between trial information, e.g., meta-analysis of interactions* | |
| Comment: | | | | |
| 2: For within-trial comparisons, is the effect modification similar from trial to trial? [ X ] Not applicable: no or one within-RCT comparison | | | | |
| [ ] Definitely not similar | [ ] Probably not similar or unclear | [ ] Mostly similar | [ ] Definitely similar | |
| *Effect modification reported for two or more trials and clearly different directions* | *Effect modification not reported for individual trials or too imprecise to tell* | *Effect modification reported for two or more trials, mostly similar in direction, but considerable differences in magnitude* | *Effect modification reported for two or more trials, similar in direction, only some differences in magnitude* | |
| Comment: | | | | |
| 3: For between-trial comparisons, is the number of trials large? [ ] Not applicable: no between RCT comparison | | | | |
| [ ] Very small | [ X ] Rather small or unclear | [ ] Rather large | [ ] Large | |
| *1 or 2 or in smallest subgroup; 5 or less in continuous meta-regression* | *3-4 in smallest subgroup; 6-10 in continuous meta-regression* | *5-9 in smallest subgroup; 11 to 15 in continuous meta-regression* | *10 or more in smallest subgroup; more than 15 in continuous meta-regression* | |
| Comment: | | | | |
| 4: Was the direction of effect modification correctly hypothesized a priori? | | | | |
| [ ] Definitely no | [ ] Probably no or unclear | [ X ] Probably yes | [ ] Definitely yes | |
| *Clearly post-hoc or results inconsistent with hypothesized direction or biologically very implausible* | *Vague hypothesis or hypothesized direction unclear* | *No prior protocol available but unequivocal statement of a priori hypothesis with correct direction of effect modification* | *Prior protocol available and includes correct specification of direction of effect modification, e.g., based on a biologic rationale* | |
| Comment: | | | | |
| 5: Does a test for interaction suggest that chance is an unlikely explanation of the apparent effect modification? (consider irrespective of number of effect modifiers) | | | | |
| [ ] Chance a very likely explanation | [ X ] Chance a likely explanation or unclear | [ ] Chance may not explain | [ ] Chance an unlikely explanation | |
| *Interaction or meta-regression p-value >0.05* | *Interaction or meta-regression p-value ≤0.05 and >0.01, or no test of interaction reported and not computable* | *Interaction or meta-regression p-value ≤0.01 and >0.005* | *Interaction or meta-regression p-value ≤0.005* | |
| Comment: | | | | |
| 6: Did the authors test only a small number of effect modifiers or consider the number in their statistical analysis? | | | | |
| [ ] Definitely no | [ ] Probably no or unclear | [ X ] Probably yes | [ ] Definitely yes | |
| *Explicitly exploratory analysis or large number of effect modifiers tested (e.g., greater than 10) and multiplicity not considered in analysis* | *No mention of number or 4-10 effect modifiers tested and number not considered in analysis* | *No protocol available but unequivocal statement of 3 or fewer effect modifiers tested* | *Protocol available and 3 or fewer effect modifiers tested or number considered in analysis* | |
| Comment: | | | | |
| 7: Did the authors use a random effects model? | | | | |
| [ ] Definitely no | [ ] Probably no or unclear | [ ] Probably yes | [ X ] Definitely yes | |
| *Fixed (or common) effect or fixed effects model explicitly stated* | *Probably fixed effect(s) model* | *Probably random (or mixed) effects* | *Random (or mixed) effects explicitly stated* | |
| Comment: | | | | |
| 8: If the effect modifier is a continuous variable, were arbitrary cut points avoided? [ X ] not applicable: not continuous | | | | |
| [ ] Definitely no | [ ] Probably no or unclear | [ ] Probably yes | [ ] Definitely yes | |
| *Analysis based on exploratory cut point(s), e.g., picking cut point associated with highest interaction p-value* | *Analysis based on cut point(s) of unclear origin* | *Analysis based on pre-specified cut point(s), e.g., suggested by prior RCT* | *Analysis based on the full continuum, e.g., assuming a linear or logarithmic relationship* | |
| Comment: | | | | |
| 9 Optional: Are there any additional considerations that may increase or decrease credibility? (manual section 3.9) [ ] not applicable | | | | |
|  | [ ] Yes, probably decrease | [ ] Yes, probably increase | | |
| Comment:   \| 10: How would you rate the overall credibility of the proposed effect modification?  The overall rating should be driven by the items that decrease credibility. The following provides a sensible strategy:  All responses definitely or probably decrease credibility or unclear 🡪 very low  Two or more responses definitely decrease credibility 🡪 maximum usually low even if all other responses satisfy credibility criteria  One response definitely decreases credibility 🡪 maximum usually moderate even if all other responses satisfy credibility criteria  Two responses probably decrease credibility 🡪 maximum usually moderate even if all other responses satisfy credibility criteria  No response options definitely or probably decrease credibility 🡪 high very likely  Place a mark on the continuous line (or type “x” in editable version) \| \| \| \| \|  \| \| --- \| --- \| --- \| --- \| --- \| --- \| \|  \|  \| \| \| \|  \| \|  \| X \| \| \| \|  \| \|  \|  \| \|  \|  \| \| \| \|  \| \|  \|  \| \| \| \|  \| \|  \| Very low credibility \| Low credibility \| Moderate credibility \| High credibility \|  \| \|  \|  \|  \|  \|  \|  \| \|  \| Minimal to no support for effect modification;  Use overall effect for each subgroup \| Some but insufficient support for effect modification;  Use overall effect for each subgroup but note remaining uncertainty \| Likely effect modification;  Use separate effects for each subgroup but note remaining uncertainty \| Very likely effect modification;  Use separate effects for each subgroup \|  \| \|  \| \| \| \| \| \| | | | | |
|  | | | | |

### Health-related quality of life: Subgroup analysis per intervention type

| CREDIBILITY ASSESSMENT | | | | |
| --- | --- | --- | --- | --- |
| Essential preliminary considerations to define the possible effect modification of interest | | | |  |
| State a single candidate effect modifier (e.g., age or comorbidity): | | | |  |
| Was the effect modifier measured before or at randomization? [ X ] yes, continue [ ] no, stop here and refer to manual for further instructions | | | |  |
| State a single outcome and time-point (e.g., mortality at 1 year follow-up): | | | |  |
| State a single effect measure (e.g., relative risk or risk difference): | | | |  |
| 1: Is the analysis of effect modification based on comparison within rather than between trials? | | | | |
| [ X ] Completely between | [ ] Mostly between or unclear | [ ] Mostly within | [ ] Completely within | |
| *Subgroup analysis or meta-regression comparing overall effects of each individual trial. This is typical for aggregate data meta-analysis.* | *Subgroup analysis or meta-regression with most information coming from overall effects, but some trials providing within-trial subgroup information* | *Most trials providing within-trial subgroup information; or individual participant data analysis that combines within and between trial information* | *All trials providing within-trial subgroup information or individual participant data; and the analysis separates within from between trial information, e.g., meta-analysis of interactions* | |
| Comment: | | | | |
| 2: For within-trial comparisons, is the effect modification similar from trial to trial? [ X ] Not applicable: no or one within-RCT comparison | | | | |
| [ ] Definitely not similar | [ ] Probably not similar or unclear | [ ] Mostly similar | [ ] Definitely similar | |
| *Effect modification reported for two or more trials and clearly different directions* | *Effect modification not reported for individual trials or too imprecise to tell* | *Effect modification reported for two or more trials, mostly similar in direction, but considerable differences in magnitude* | *Effect modification reported for two or more trials, similar in direction, only some differences in magnitude* | |
| Comment: | | | | |
| 3: For between-trial comparisons, is the number of trials large? [ ] Not applicable: no between RCT comparison | | | | |
| [ X ] Very small | [ ] Rather small or unclear | [ ] Rather large | [ ] Large | |
| *1 or 2 or in smallest subgroup; 5 or less in continuous meta-regression* | *3-4 in smallest subgroup; 6-10 in continuous meta-regression* | *5-9 in smallest subgroup; 11 to 15 in continuous meta-regression* | *10 or more in smallest subgroup; more than 15 in continuous meta-regression* | |
| Comment: | | | | |
| 4: Was the direction of effect modification correctly hypothesized a priori? | | | | |
| [ ] Definitely no | [ ] Probably no or unclear | [ X ] Probably yes | [ ] Definitely yes | |
| *Clearly post-hoc or results inconsistent with hypothesized direction or biologically very implausible* | *Vague hypothesis or hypothesized direction unclear* | *No prior protocol available but unequivocal statement of a priori hypothesis with correct direction of effect modification* | *Prior protocol available and includes correct specification of direction of effect modification, e.g., based on a biologic rationale* | |
| Comment: | | | | |
| 5: Does a test for interaction suggest that chance is an unlikely explanation of the apparent effect modification? (consider irrespective of number of effect modifiers) | | | | |
| [ ] Chance a very likely explanation | [ ] Chance a likely explanation or unclear | [ ] Chance may not explain | [ X ] Chance an unlikely explanation | |
| *Interaction or meta-regression p-value >0.05* | *Interaction or meta-regression p-value ≤0.05 and >0.01, or no test of interaction reported and not computable* | *Interaction or meta-regression p-value ≤0.01 and >0.005* | *Interaction or meta-regression p-value ≤0.005* | |
| Comment: | | | | |
| 6: Did the authors test only a small number of effect modifiers or consider the number in their statistical analysis? | | | | |
| [ ] Definitely no | [ ] Probably no or unclear | [ X ] Probably yes | [ ] Definitely yes | |
| *Explicitly exploratory analysis or large number of effect modifiers tested (e.g., greater than 10) and multiplicity not considered in analysis* | *No mention of number or 4-10 effect modifiers tested and number not considered in analysis* | *No protocol available but unequivocal statement of 3 or fewer effect modifiers tested* | *Protocol available and 3 or fewer effect modifiers tested or number considered in analysis* | |
| Comment: | | | | |
| 7: Did the authors use a random effects model? | | | | |
| [ ] Definitely no | [ ] Probably no or unclear | [ ] Probably yes | [ X ] Definitely yes | |
| *Fixed (or common) effect or fixed effects model explicitly stated* | *Probably fixed effect(s) model* | *Probably random (or mixed) effects* | *Random (or mixed) effects explicitly stated* | |
| Comment: | | | | |
| 8: If the effect modifier is a continuous variable, were arbitrary cut points avoided? [ ] not applicable: not continuous | | | | |
| [ ] Definitely no | [ ] Probably no or unclear | [ ] Probably yes | [ X ] Definitely yes | |
| *Analysis based on exploratory cut point(s), e.g., picking cut point associated with highest interaction p-value* | *Analysis based on cut point(s) of unclear origin* | *Analysis based on pre-specified cut point(s), e.g., suggested by prior RCT* | *Analysis based on the full continuum, e.g., assuming a linear or logarithmic relationship* | |
| Comment: | | | | |
| 9 Optional: Are there any additional considerations that may increase or decrease credibility? (manual section 3.9) [ ] not applicable | | | | |
|  | [ ] Yes, probably decrease | [ ] Yes, probably increase | | |
| Comment:   \| 10: How would you rate the overall credibility of the proposed effect modification?  The overall rating should be driven by the items that decrease credibility. The following provides a sensible strategy:  All responses definitely or probably decrease credibility or unclear 🡪 very low  Two or more responses definitely decrease credibility 🡪 maximum usually low even if all other responses satisfy credibility criteria  One response definitely decreases credibility 🡪 maximum usually moderate even if all other responses satisfy credibility criteria  Two responses probably decrease credibility 🡪 maximum usually moderate even if all other responses satisfy credibility criteria  No response options definitely or probably decrease credibility 🡪 high very likely  Place a mark on the continuous line (or type “x” in editable version) \| \| \| \| \|  \| \| --- \| --- \| --- \| --- \| --- \| --- \| \|  \|  \| \| \| \|  \| \|  \| X \| \| \| \|  \| \|  \|  \| \|  \|  \| \| \| \|  \| \|  \|  \| \| \| \|  \| \|  \| Very low credibility \| Low credibility \| Moderate credibility \| High credibility \|  \| \|  \|  \|  \|  \|  \|  \| \|  \| Minimal to no support for effect modification;  Use overall effect for each subgroup \| Some but insufficient support for effect modification;  Use overall effect for each subgroup but note remaining uncertainty \| Likely effect modification;  Use separate effects for each subgroup but note remaining uncertainty \| Very likely effect modification;  Use separate effects for each subgroup \|  \| \|  \| \| \| \| \| \| | | | | |

### Health-related quality of life: Subgroup analysis per risk of bias

| CREDIBILITY ASSESSMENT | | | | |
| --- | --- | --- | --- | --- |
| Essential preliminary considerations to define the possible effect modification of interest | | | |  |
| State a single candidate effect modifier (e.g., age or comorbidity): | | | |  |
| Was the effect modifier measured before or at randomization? [ X ] yes, continue [ ] no, stop here and refer to manual for further instructions | | | |  |
| State a single outcome and time-point (e.g., mortality at 1 year follow-up): | | | |  |
| State a single effect measure (e.g., relative risk or risk difference): | | | |  |
| 1: Is the analysis of effect modification based on comparison within rather than between trials? | | | | |
| [ X ] Completely between | [ ] Mostly between or unclear | [ ] Mostly within | [ ] Completely within | |
| *Subgroup analysis or meta-regression comparing overall effects of each individual trial. This is typical for aggregate data meta-analysis.* | *Subgroup analysis or meta-regression with most information coming from overall effects, but some trials providing within-trial subgroup information* | *Most trials providing within-trial subgroup information; or individual participant data analysis that combines within and between trial information* | *All trials providing within-trial subgroup information or individual participant data; and the analysis separates within from between trial information, e.g., meta-analysis of interactions* | |
| Comment: | | | | |
| 2: For within-trial comparisons, is the effect modification similar from trial to trial? [ X ] Not applicable: no or one within-RCT comparison | | | | |
| [ ] Definitely not similar | [ ] Probably not similar or unclear | [ ] Mostly similar | [ ] Definitely similar | |
| *Effect modification reported for two or more trials and clearly different directions* | *Effect modification not reported for individual trials or too imprecise to tell* | *Effect modification reported for two or more trials, mostly similar in direction, but considerable differences in magnitude* | *Effect modification reported for two or more trials, similar in direction, only some differences in magnitude* | |
| Comment: | | | | |
| 3: For between-trial comparisons, is the number of trials large? [ ] Not applicable: no between RCT comparison | | | | |
| [ X ] Very small | [ ] Rather small or unclear | [ ] Rather large | [ ] Large | |
| *1 or 2 or in smallest subgroup; 5 or less in continuous meta-regression* | *3-4 in smallest subgroup; 6-10 in continuous meta-regression* | *5-9 in smallest subgroup; 11 to 15 in continuous meta-regression* | *10 or more in smallest subgroup; more than 15 in continuous meta-regression* | |
| Comment: | | | | |
| 4: Was the direction of effect modification correctly hypothesized a priori? | | | | |
| [ ] Definitely no | [ ] Probably no or unclear | [ X ] Probably yes | [ ] Definitely yes | |
| *Clearly post-hoc or results inconsistent with hypothesized direction or biologically very implausible* | *Vague hypothesis or hypothesized direction unclear* | *No prior protocol available but unequivocal statement of a priori hypothesis with correct direction of effect modification* | *Prior protocol available and includes correct specification of direction of effect modification, e.g., based on a biologic rationale* | |
| Comment: | | | | |
| 5: Does a test for interaction suggest that chance is an unlikely explanation of the apparent effect modification? (consider irrespective of number of effect modifiers) | | | | |
| [ ] Chance a very likely explanation | [ ] Chance a likely explanation or unclear | [ ] Chance may not explain | [ X ] Chance an unlikely explanation | |
| *Interaction or meta-regression p-value >0.05* | *Interaction or meta-regression p-value ≤0.05 and >0.01, or no test of interaction reported and not computable* | *Interaction or meta-regression p-value ≤0.01 and >0.005* | *Interaction or meta-regression p-value ≤0.005* | |
| Comment: | | | | |
| 6: Did the authors test only a small number of effect modifiers or consider the number in their statistical analysis? | | | | |
| [ ] Definitely no | [ ] Probably no or unclear | [ X ] Probably yes | [ ] Definitely yes | |
| *Explicitly exploratory analysis or large number of effect modifiers tested (e.g., greater than 10) and multiplicity not considered in analysis* | *No mention of number or 4-10 effect modifiers tested and number not considered in analysis* | *No protocol available but unequivocal statement of 3 or fewer effect modifiers tested* | *Protocol available and 3 or fewer effect modifiers tested or number considered in analysis* | |
| Comment: | | | | |
| 7: Did the authors use a random effects model? | | | | |
| [ ] Definitely no | [ ] Probably no or unclear | [ ] Probably yes | [ X ] Definitely yes | |
| *Fixed (or common) effect or fixed effects model explicitly stated* | *Probably fixed effect(s) model* | *Probably random (or mixed) effects* | *Random (or mixed) effects explicitly stated* | |
| Comment: | | | | |
| 8: If the effect modifier is a continuous variable, were arbitrary cut points avoided? [ ] not applicable: not continuous | | | | |
| [ ] Definitely no | [ ] Probably no or unclear | [ ] Probably yes | [ X ] Definitely yes | |
| *Analysis based on exploratory cut point(s), e.g., picking cut point associated with highest interaction p-value* | *Analysis based on cut point(s) of unclear origin* | *Analysis based on pre-specified cut point(s), e.g., suggested by prior RCT* | *Analysis based on the full continuum, e.g., assuming a linear or logarithmic relationship* | |
| Comment: | | | | |
| 9 Optional: Are there any additional considerations that may increase or decrease credibility? (manual section 3.9) [ ] not applicable | | | | |
|  | [ ] Yes, probably decrease | [ ] Yes, probably increase | | |
| Comment:   \| 10: How would you rate the overall credibility of the proposed effect modification?  The overall rating should be driven by the items that decrease credibility. The following provides a sensible strategy:  All responses definitely or probably decrease credibility or unclear 🡪 very low  Two or more responses definitely decrease credibility 🡪 maximum usually low even if all other responses satisfy credibility criteria  One response definitely decreases credibility 🡪 maximum usually moderate even if all other responses satisfy credibility criteria  Two responses probably decrease credibility 🡪 maximum usually moderate even if all other responses satisfy credibility criteria  No response options definitely or probably decrease credibility 🡪 high very likely  Place a mark on the continuous line (or type “x” in editable version) \| \| \| \| \|  \| \| --- \| --- \| --- \| --- \| --- \| --- \| \|  \|  \| \| \| \|  \| \|  \| X \| \| \| \|  \| \|  \|  \| \|  \|  \| \| \| \|  \| \|  \|  \| \| \| \|  \| \|  \| Very low credibility \| Low credibility \| Moderate credibility \| High credibility \|  \| \|  \|  \|  \|  \|  \|  \| \|  \| Minimal to no support for effect modification;  Use overall effect for each subgroup \| Some but insufficient support for effect modification;  Use overall effect for each subgroup but note remaining uncertainty \| Likely effect modification;  Use separate effects for each subgroup but note remaining uncertainty \| Very likely effect modification;  Use separate effects for each subgroup \|  \| \|  \| \| \| \| \| \| | | | | |

# Appendix 5: Measurement instruments used in the studies

### Health-related quality of life

- Minnesota Living with Heart Failure Questionnaire(Rector, 1987)
- Stroke-Adapted Sickness Impact Profile (SA-SIP30)(van Straten et al., 1997)
- Chronic Respiratory Disease Questionnaire (CRDQ)(Guyatt et al., 1987)

### Physical and mental quality of life

- Veterans Rand 12-item health survey (VR-12)(Kazis et al., 2006)
- Short Form-36 Item Health Survey (SF-36)(Ware Jr & Sherbourne, 1992)
- Short Form-12 Item Health Survey (SF-12)(Ware et al., 1995)

### Physical performance

- Katz ADL(Katz et al., 1963)
- Barthel index(Mahoney & Barthel, 1965)
- Modified Barthel index(Shah et al., 1989)
- Enforced Social Dependency Scale (ESDS)(Benoliel et al., 1980)
- Functional Activities Questionnaire (FAQ)(Pfeffer et al., 1982)
- Functional Autonomy Measurement System (SMAF)(Hebert et al., 1988)

### Physical capacity

- 6-minute walk test(Steele, 1996)
- Timed up and go test(Podsiadlo & Richardson, 1991)
- Lindmark Motor Capacity(Lindmark, 1988)

# Appendix 6: References of the included studies

Allen, K. R., Hazelett, S., Jarjoura, D., Wickstrom, G. C., Hua, K., Weinhardt, J., & Wright, K. (2002). Effectiveness of a postdischarge care management model for stroke and transient ischemic attack: a randomized trial. *Journal of Stroke and Cerebrovascular Diseases*, *11*(2), 88-98.

Altfeld, S. J., Shier, G. E., Rooney, M., Johnson, T. J., Golden, R. L., Karavolos, K., Avery, E., Nandi, V., & Perry, A. J. (2013). Effects of an enhanced discharge planning intervention for hospitalized older adults: a randomized trial. *The Gerontologist*, *53*(3), 430-440.

Atwood, C. E., Bhutani, M., Ospina, M. B., Rowe, B. H., Leigh, R., Deuchar, L., Faris, P., Michas, M., Mrklas, K. J., & Graham, J. (2022). Optimizing COPD acute care patient outcomes using a standardized transition bundle and care coordinator: a randomized clinical trial. *Chest*, *162*(2), 321-330.

Baghaei, R., Parizad, N., Sharifi, A., & Alinejad, V. (2021). The Effect of Continuous Nursing Care Program on Anxiety Level, Episodes of Chest Pain, and Readmission Rate after Myocardial Infarction: A Randomized Controlled Trial. *International Cardiovascular Research Journal*, *15*(1).

Balaban, R. B., Galbraith, A. A., Burns, M. E., Vialle-Valentin, C. E., Larochelle, M. R., & Ross-Degnan, D. (2015). A patient navigator intervention to reduce hospital readmissions among high-risk safety-net patients: a randomized controlled trial. *Journal of General Internal Medicine*, *30*, 907-915.

Balaban, R. B., Weissman, J. S., Samuel, P. A., & Woolhandler, S. (2008). Redefining and redesigning hospital discharge to enhance patient care: a randomized controlled study. *Journal of General Internal Medicine*, *23*, 1228-1233.

Balaban, R. B., Zhang, F., Vialle-Valentin, C. E., Galbraith, A. A., Burns, M. E., Larochelle, M. R., & Ross-Degnan, D. (2017). Impact of a patient navigator program on hospital-based and outpatient utilization over 180 days in a safety-net health system. *Journal of General Internal Medicine*, *32*, 981-989.

Casas, A., Troosters, T., Garcia-Aymerich, J., Roca, J., Hernández, C., Alonso, A., del Pozo, F., de Toledo, P., Antó, J. M., & Rodríguez-Roisín, R. (2006). Integrated care prevents hospitalisations for exacerbations in COPD patients. *European Respiratory Journal*, *28*(1), 123-130.

Coskun, S., & Duygulu, S. (2022). The effects of Nurse Led Transitional Care Model on elderly patients undergoing open heart surgery: a randomized controlled trial. *European Journal of Cardiovascular Nursing*, *21*(1), 46-55.

Courtney, M., Edwards, H., Chang, A., Parker, A., Finlayson, K., & Hamilton, K. (2009). Fewer emergency readmissions and better quality of life for older adults at risk of hospital readmission: A randomized controlled trial to determine the effectiveness of a 24‐week exercise and telephone follow‐up program. *Journal of the American Geriatrics Society*, *57*(3), 395-402.

Davidson, P. M., Cockburn, J., Newton, P. J., Webster, J. K., Betihavas, V., Howes, L., & Owensbye, D. O. (2010). Can a heart failure-specific cardiac rehabilitation program decrease hospitalizations and improve outcomes in high-risk patients? *European Journal of Preventive Cardiology*, *17*(4), 393-402.

Del Sindaco, D., Pulignano, G., Minardi, G., Apostoli, A., Guerrieri, L., Rotoloni, M., Petri, G., Fabrizi, L., Caroselli, A., & Venusti, R. (2007). Two-year outcome of a prospective, controlled study of a disease management programme for elderly patients with heart failure. *Journal of Cardiovascular Medicine*, *8*(5), 324-329.

Deng, A., Zhang, Y., & Xiong, R. (2021). Effects of a transitional care program for individuals with limbs disabilities living in a rural community: A randomized controlled trial. *Disability and Health Journal*, *14*(1), 100946.

Dhalla, I. A., O’Brien, T., Morra, D., Thorpe, K. E., Wong, B. M., Mehta, R., Frost, D. W., Abrams, H., Ko, F., & Van Rooyen, P. (2014). Effect of a postdischarge virtual ward on readmission or death for high-risk patients: a randomized clinical trial. *Jama*, *312*(13), 1305-1312.

Donnelly, M., Power, M., Russell, M., & Fullerton, K. (2004). Randomized controlled trial of an early discharge rehabilitation service: the Belfast Community Stroke Trial. *Stroke*, *35*(1), 127-133.

Evangelista, A., Camussi, E., Corezzi, M., Gilardetti, M., Fonte, G., Scarmozzino, A., La Valle, G., Angelone, L., Olivero, E., & Ciccone, G. (2023). Routine vs. On-Demand Discharge Planning Strategy in Intermediate-Risk Patients for Complex Discharge: a Cluster-Randomized, Multiple Crossover Trial. *Journal of General Internal Medicine*, *38*(12), 2749-2754.

Evans, R. L., & Hendricks, R. D. (1993). Evaluating hospital discharge planning: a randomized clinical trial. *Medical care*, *31*(4), 358-370.

Finkelstein, A., Zhou, A., Taubman, S., & Doyle, J. (2020). Health care hotspotting—a randomized, controlled trial. *New England Journal of Medicine*, *382*(2), 152-162.

Finlayson, K., Chang, A. M., Courtney, M. D., Edwards, H. E., Parker, A. W., Hamilton, K., Pham, T. D. X., & O’Brien, J. (2018). Transitional care interventions reduce unplanned hospital readmissions in high-risk older adults. *BMC health services research*, *18*, 1-9.

Fjærtoft, H., Indredavik, B., & Lydersen, S. (2003). Stroke unit care combined with early supported discharge: long-term follow-up of a randomized controlled trial. *Stroke*, *34*(11), 2687-2691.

Fjærtoft, H., Rohweder, G., & Indredavik, B. (2011). Stroke unit care combined with early supported discharge improves 5-year outcome: a randomized controlled trial. *Stroke*, *42*(6), 1707-1711.

Garcia-Aymerich, J., Hernandez, C., Alonso, A., Casas, A., Rodriguez-Roisin, R., Anto, J. M., & Roca, J. (2007). Effects of an integrated care intervention on risk factors of COPD readmission. *Respiratory medicine*, *101*(7), 1462-1469.

Hofstad, H., Gjelsvik, B. E., Næss, H., Eide, G. E., & Skouen, J. S. (2014). Early supported discharge after stroke in Bergen (ESD Stroke Bergen): three and six months results of a randomised controlled trial comparing two early supported discharge schemes with treatment as usual. *BMC Neurol*, *14*, 239. <https://doi.org/10.1186/s12883-014-0239-3>

Hu, R., Gu, B., Tan, Q., Xiao, K., Li, X., Cao, X., Song, T., & Jiang, X. (2020). The effects of a transitional care program on discharge readiness, transitional care quality, health services utilization and satisfaction among Chinese kidney transplant recipients: A randomized controlled trial. *International Journal of Nursing Studies*, *110*, 103700.

Hung, C.-C., Kao, H.-F. S., Jimenez, S. Y., Tonapa, S. I., & Lee, B.-O. (2023). Effects of case management in trauma patients in Taiwan: a randomized, longitudinal study. *Journal of Trauma Nursing| JTN*, *30*(4), 213-221.

Jack, B. W., Chetty, V. K., Anthony, D., Greenwald, J. L., Sanchez, G. M., Johnson, A. E., Forsythe, S. R., O'Donnell, J. K., Paasche-Orlow, M. K., & Manasseh, C. (2009). A reengineered hospital discharge program to decrease rehospitalization: a randomized trial. *Annals of internal medicine*, *150*(3), 178-187.

Jackson, J. C., Ely, E. W., Morey, M. C., Anderson, V. M., Denne, L. B., Clune, J., Siebert, C. S., Archer, K. R., Torres, R., & Janz, D. (2012). Cognitive and physical rehabilitation of intensive care unit survivors: results of the RETURN randomized controlled pilot investigation. *Critical care medicine*, *40*(4), 1088-1097.

Jepma, P., Verweij, L., Buurman, B. M., Terbraak, M. S., Daliri, S., Latour, C. H., Ter Riet, G., Karapinar-Çarkit, F., Dekker, J., & Klunder, J. L. (2021). The nurse-coordinated cardiac care bridge transitional care programme: a randomised clinical trial. *Age and ageing*, *50*(6), 2105-2115.

Ko, F. W., Cheung, N., Rainer, T. H., Lum, C., Wong, I., & Hui, D. S. (2017). Comprehensive care programme for patients with chronic obstructive pulmonary disease: a randomised controlled trial. *Thorax*, *72*(2), 122-128.

Lainscak, M., Kadivec, S., Kosnik, M., Benedik, B., Bratkovic, M., Jakhel, T., Marcun, R., Miklosa, P., Stalc, B., & Farkas, J. (2013). Discharge coordinator intervention prevents hospitalizations in patients with COPD: a randomized controlled trial. *Journal of the American Medical Directors Association*, *14*(6), 450. e451-450. e456.

Lanzeta, I., Mar, J., & Arrospide, A. (2016). Cost-utility analysis of an integrated care model for multimorbid patients based on a clinical trial. *Gaceta sanitaria*, *30*, 352-358.

Latour, C. H., de Vos, R., Huyse, F. J., de Jonge, P., Van Gemert, L. A., & Stalman, W. A. (2006). Effectiveness of post-discharge case management in general-medical outpatients: a randomized, controlled trial. *Psychosomatics*, *47*(5), 421-429.

Lim, W. K., Lambert, S. F., & Gray, L. C. (2003). Effectiveness of case management and post‐acute services in older people after hospital discharge. *Medical Journal of Australia*, *178*(6), 262-266.

Linden, A., & Butterworth, S. W. (2014). A comprehensive hospital-based intervention to reduce readmissions for chronically ill patients: a randomized controlled trial. *Am J Manag Care*, *20*(10), 783-792.

Liu, X., Song, L., Xiao, S., & Wang, Y. (2023). Comprehensive Geriatric Assessment, Multidisciplinary Treatment, and Nurse-Guided Transitional Care in Hospitalized Older Adults: A Randomized Controlled Trial. *Research in Gerontological Nursing*, *16*(5), 224-230.

Markle-Reid, M., Fisher, K., Walker, K. M., Beauchamp, M., Cameron, J. I., Dayler, D., Fleck, R., Gafni, A., Ganann, R., & Hajas, K. (2023). The stroke transitional care intervention for older adults with stroke and multimorbidity: a multisite pragmatic randomized controlled trial. *BMC geriatrics*, *23*(1), 687.

Markle-Reid, M., McAiney, C., Fisher, K., Ganann, R., Gauthier, A. P., Heald-Taylor, G., McElhaney, J. E., McMillan, F., Petrie, P., & Ploeg, J. (2021). Effectiveness of a nurse-led hospital-to-home transitional care intervention for older adults with multimorbidity and depressive symptoms: a pragmatic randomized controlled trial. *PLoS One*, *16*(7), e0254573.

McCorkle, R., Nuamah, I., Strumpf, N., Adler, D. C., Cooley, M. E., Jepson, C., Lusk, E. J., & Torosian, M. (2000). A specialized home care intervention improves survival among older post-surgical cancer patients. *Journal of the American Geriatrics Society*, *48*(12), 1707-1713. <https://doi.org/10.1111/j.1532-5415.2000.tb03886.x>

Meyer, A. M., Bartram, M. P., Antczak, P., Becker, I., Benzing, T., & Polidori, M. C. (2022). A Tailored Discharge Program Improves Frailty and Mood in Patients Undergoing Usual Rehabilitative Care: A Randomized Controlled Trial. *Journal of the American Medical Directors Association*, *23*(12), 1962. e1961-1962. e1913.

Naylor, M. D., Brooten, D. A., Campbell, R. L., Maislin, G., McCauley, K. M., & Schwartz, J. S. (2004). Transitional care of older adults hospitalized with heart failure: a randomized, controlled trial. *Journal of the American Geriatrics Society*, *52*(5), 675-684.

Preen, D. B., Bailey, B. E., Wright, A., Kendall, P., Phillips, M., Hung, J., Hendriks, R., Mather, A., & Williams, E. (2005). Effects of a multidisciplinary, post-discharge continuance of care intervention on quality of life, discharge satisfaction, and hospital length of stay: a randomized controlled trial. *International Journal for Quality in Health Care*, *17*(1), 43-51.

Rich, M. W., Vinson, J. M., Sperry, J. C., Shah, A. S., Spinner, L. R., Chung, M. K., & Da Vila-Roman, V. (1993). Prevention of readmission in elderly patients with congestive heart failure: results of a prospective, randomized pilot study. *Journal of General Internal Medicine*, *8*, 585-590.

Santana, M. J., Holroyd-Leduc, J., Southern, D. A., Flemons, W. W., O’Beirne, M., Hill, M. D., Forster, A. J., White, D. E., & Ghali, W. A. (2017). A randomised controlled trial assessing the efficacy of an electronic discharge communication tool for preventing death or hospital readmission. *BMJ Quality & Safety*, *26*(12), 993-1003.

Schnipper, J. L., Samal, L., Nolido, N., Yoon, C., Dalal, A. K., Magny‐Normilus, C., Bitton, A., Thompson, R., Labonville, S., & Crevensten, G. (2021). The effects of a multifaceted intervention to improve care transitions within an accountable care organization: results of a stepped‐wedge cluster‐randomized trial. *Journal of Hospital Medicine*, *16*(1), 15-22.

Thorsén, A.-M., Widés Holmqvist, L., de Pedro-Cuesta, J., & von Koch, L. (2005). A randomized controlled trial of early supported discharge and continued rehabilitation at home after stroke: five-year follow-up of patient outcome. *Stroke*, *36*(2), 297-303.

Thygesen, L. C., Fokdal, S., Gjørup, T., Taylor, R. S., Zwisler, A.-D., & Group, P. o. E. R. R. (2015). Can municipality-based post-discharge follow-up visits including a general practitioner reduce early readmission among the fragile elderly (65+ years old)? A randomized controlled trial. *Scandinavian journal of primary health care*, *33*(2), 65-73.

Van Spall, H. G., Lee, S. F., Xie, F., Oz, U. E., Perez, R., Mitoff, P. R., Maingi, M., Tjandrawidjaja, M. C., Heffernan, M., & Zia, M. I. (2019). Effect of patient-centered transitional care services on clinical outcomes in patients hospitalized for heart failure: the PACT-HF randomized clinical trial. *Jama*, *321*(8), 753-761.

Visperas, A. T., Greene, K. A., Krebs, V. E., Klika, A. K., Piuzzi, N. S., & Higuera-Rueda, C. A. (2021). A web-based interactive patient-provider software platform does not increase patient satisfaction or decrease hospital resource utilization in total knee and hip arthroplasty patients in a single large hospital system. *The Journal of Arthroplasty*, *36*(7), 2290-2296. e2291.

Zimmerman, W. D., Grenier, R. E., Palka, S. V., Monacci, K. J., Lantzy, A. K., Leutbecker, J. A., Geng, X., & Denny, M. C. (2021). Transitions of care coordination intervention identifies barriers to discharge in hospitalized stroke patients. *Frontiers in Neurology*, *12*, 573294.

# References

Benoliel, J. Q., McCorkle, R., & Young, K. (1980). Development of a social dependency scale. *Research in Nursing & Health*, *3*(1), 3-10.

Guyatt, G. H., Berman, L. B., Townsend, M., Pugsley, S. O., & Chambers, L. W. (1987). A measure of quality of life for clinical trials in chronic lung disease. *Thorax*, *42*(10), 773-778.

Hebert, R., Carrier, R., & Bilodeau, A. (1988). The Functional Autonomy Measurement System (SMAF): description and validation of an instrument for the measurement of handicaps. *Age and ageing*, *17*(5), 293-302.

Husereau, D., Drummond, M., Augustovski, F., de Bekker-Grob, E., Briggs, A. H., Carswell, C., Caulley, L., Chaiyakunapruk, N., Greenberg, D., & Loder, E. (2022). Consolidated Health Economic Evaluation Reporting Standards 2022 (CHEERS 2022) statement: updated reporting guidance for health economic evaluations. *MDM Policy & Practice*, *7*(1), 23814683211061097.

Katz, S., Ford, A. B., Moskowitz, R. W., Jackson, B. A., & Jaffe, M. W. (1963). Studies of illness in the aged: the index of ADL: a standardized measure of biological and psychosocial function. *Jama*, *185*(12), 914-919.

Kazis, L. E., Miller, D. R., Skinner, K. M., Lee, A., Ren, X. S., Clark, J. A., Rogers, W. H., Spiro III, A., Selim, A., & Linzer, M. (2006). Applications of methodologies of the Veterans Health Study in the VA healthcare system: conclusions and summary. *The Journal of ambulatory care management*, *29*(2), 182-188.

Lindmark, B. (1988). Evaluation of functional capacity after stroke with special emphasis on motor function and activities of daily living. *Scandinavian journal of rehabilitation medicine. Supplement*, *21*, 1-40.

Mahoney, F. I., & Barthel, D. W. (1965). Functional evaluation: the Barthel Index: a simple index of independence useful in scoring improvement in the rehabilitation of the chronically ill. *Maryland state medical journal*.

Pfeffer, R. I., Kurosaki, T. T., Harrah Jr, C., Chance, J. M., & Filos, S. (1982). Measurement of functional activities in older adults in the community. *Journal of gerontology*, *37*(3), 323-329.

Podsiadlo, D., & Richardson, S. (1991). The timed “Up & Go”: a test of basic functional mobility for frail elderly persons. *Journal of the American Geriatrics Society*, *39*(2), 142-148.

Rector, T. (1987). Patient's self-assessment of their congestive heart failure: content, reliability, validity of a new measure, the Minnesota Living with Heart Failure questionnaire. *Heart failure*, *3*, 198-209.

Shah, S., Vanclay, F., & Cooper, B. (1989). Improving the sensitivity of the Barthel Index for stroke rehabilitation. *Journal of clinical epidemiology*, *42*(8), 703-709.

Steele, B. (1996). Timed walking tests of exercise capacity in chronic cardiopulmonary illness. *Journal of Cardiopulmonary Rehabilitation and Prevention*, *16*(1), 25-33.

van Straten, A., de Haan, R. J., Limburg, M., Schuling, J., Bossuyt, P. M., & van den Bos, G. A. (1997). A stroke-adapted 30-item version of the Sickness Impact Profile to assess quality of life (SA-SIP30). *Stroke*, *28*(11), 2155-2161. <https://doi.org/10.1161/01.str.28.11.2155>

Ware, J. E., Keller, S. D., & Kosinski, M. (1995). *SF-12: How to score the SF-12 physical and mental health summary scales*. Health Institute, New England Medical Center.

Ware Jr, J. E., & Sherbourne, C. D. (1992). The MOS 36-item short-form health survey (SF-36): I. Conceptual framework and item selection. *Medical care*, 473-483.
